# Five-Year Outcome of Camrelizumab Plus Chemotherapy in Recurrent or Metastatic Nasopharyngeal Carcinoma: A Secondary Analysis of the CAPTAIN-1st Randomized Clinical Trial

**DOI:** 10.1001/jamaoncol.2025.6245

**Published:** 2026-01-29

**Authors:** Yan Huang, Dongchen Sun, Huaqiang Zhou, Ting Zhou, Song Qu, Jingao Li, Chaosu Hu, Mingjun Xu, Weidong Li, Liangfang Shen, Hui Wu, Jinyi Lang, Guangyuan Hu, Zhanxiong Luo, Zhichao Fu, Shenhong Qu, Weineng Feng, Xiaozhong Chen, Shaojun Lin, Bo Xie, Xiaojiang Li, Yan Sun, Zhixiong Lin, Qin Lin, Feng Lei, Jianting Long, Jinsheng Hong, Xiaoming Huang, Lingzhi Zeng, Peiguo Wang, Xiaohui He, Shen Zhao, Gang Chen, Yaxiong Zhang, Yuanyuan Zhao, Wenfeng Fang, Chuanpei Huang, Xiaotong Li, Shaodong Hong, Li Zhang, Yunpeng Yang

**Affiliations:** 1 Sun Yat-sen University Cancer Center, State Key Laboratory of Oncology in South China, Guangdong Key Laboratory of Nasopharyngeal Carcinoma Diagnosis and Therapy, Guangdong Provincial Clinical Research Center for Cancer, Collaborative Innovation Center for Cancer Medicine, Guangzhou, China; 2 Guangxi Medical University Affiliated Tumor Hospital, Nanning, China; 3 Jiangxi Cancer Hospital, The Second Affiliated Hospital of Nanchang Medical School, Nanchang, China.; 4 Fudan University Shanghai Cancer Center, Shanghai, China; 5 First Affiliated Hospital of Gannan Medical University, Ganzhou, China; 6 Guangzhou Medical University Affiliated Oncology Hospital, Guangzhou, China; 7 Xiangya Hospital Central South University, Changsha, China; 8 Affiliated Cancer Hospital of Zhengzhou University, Zhengzhou, China; 9 Sichuan Cancer Hospital & Institute, Sichuan Cancer Center, School of Medicine, University of Electronic Science and Technology of China, Chengdu, China; 10 Tongji Hospital of Tongji Medical College, Huazhong University of Science and Technology, Wuhan, China; 11 Liuzhou People’s Hospital, Liuzhou, China; 12 900th Hospital of The Joint Logistics Team, PLA, Fuzhou, China; 13 Guangxi Zhuang Autonomous Region People’s Hospital, Nanning, China; 14 The First People’s Hospital of Foshan, Foshan, China; 15 Zhejiang Cancer Hospital, Hangzhou, China; 16 Fujian Medical University Cancer Hospital & Fujian Cancer Hospital, Fuzhou, China; 17 General Hospital of Southern Theatre Command, Guangzhou, China; 18 Yunnan Cancer Hospital, Kunming, China; 19 Beijing Cancer Hospital, Beijing, China; 20 Shantou University Medical College Affiliated Cancer Hospital, Shantou, China; 21 The First Affiliated Hospital of Xiamen University, Xiamen, China; 22 Zhongshan People’s Hospital, Zhongshan, China; 23 The First Affiliated Hospital of Sun Yat-sen University, Guangzhou, China; 24 The First Affiliated Hospital of Fujian Medical University/Key Laboratory of Radiation Biology, Fuzhou, China; 25 Sun Yat-sen Memorial Hospital of Sun Yat-sen University, Guangzhou, China; 26 Jiujiang No.1 People’s Hospital of Nanchang University, Jiujiang, China; 27 Tianjin Medical University Cancer Institute and Hospital, Tianjin, China; 28 Cancer Hospital Chinese Academy of Medical Sciences, Beijing, China; 29 Jiangsu Hengrui Pharmaceuticals Co, Ltd, Shanghai, China

## Abstract

**IMPORTANCE:**

Programmed cell death 1 protein (PD-1) or programmed cell death 1 ligand 1 inhibitors plus chemotherapy is the current standard first-line treatment of recurrent or metastatic nasopharyngeal carcinoma (RM-NPC). However, the long-term survival benefits at the 5-year benchmark remain uncertain.

**OBJECTIVE:**

To determine whether adding camrelizumab to chemotherapy significantly improved 5-year overall survival (OS) as first-line treatment for RM-NPC, compared with chemotherapy alone.

**DESIGN, SETTING, AND PARTICIPANTS:**

The CAPTAIN-1st trial was a randomized, double-blind, phase 3 trial conducted at 28 hospitals in China. Between November 13, 2018, and November 29, 2019, patients with treatment-naive RM-NPC were enrolled. This secondary analysis of the CAPTAIN-1st trial was prespecified. Data analysis was conducted on June 1, 2025.

**INTERVENTIONS:**

Patients were randomized (1:1) to receive camrelizumab or placebo in combination with gemcitabine and cisplatin for 4 to 6 cycles, followed by maintenance therapy with camrelizumab or placebo until disease progression, unacceptable toxic effects, or completion of 2 years of treatment.

**MAIN OUTCOME:**

The primary end point, progression-free survival per independent review committee, has been reported previously. Herein, the secondary end point of OS is reported as prespecified in the protocol.

**RESULTS:**

Among 263 randomized patients (134 in randomized to camrelizumab, 129 to placebo), baseline characteristics were generally balanced between groups, except for age. The mean (SD) age was 49 (11.25) years, and 218 patients (82.9%) were male individuals, 45 (17.1%) were female individuals. With a median survival follow-up of 63.5 (95% CI, 61.2-64.6) months for the camrelizumab group and 63.0 (95% CI, 60.8-64.6) months for the placebo group, 85 (63.4%) and 95 (73.6%) deaths occurred, respectively. Median OS was 34.5 months (95% CI, 29.4-45.7) with camrelizumab vs 26.6 months (95% CI, 19.8-33.5) with placebo (hazard ratio [HR], 0.74; 95% CI, 0.55-0.99; 2-sided *P* = .047). After adjusting for age imbalance, the HR was 0.65 (95% CI, 0.48-0.89; *P* = .01). The 5-year OS rates were 37.8% vs 24.2%, reflecting an absolute difference of 13.6% (95% CI, 2.4%-24.8%; *P* = .02) in favor of camrelizumab. The OS benefits were generally consistent across subgroups. In the camrelizumab group, patients who achieved rapid clearance of Epstein-Barr virus (EBV) DNA had significantly longer OS compared with those without EBV DNA clearance (HR, 0.32; 95% CI, 0.18-0.58; *P* < .001).

**CONCLUSIONS AND RELEVANCE:**

In this secondary analysis of a randomized clinical trial, the addition of camrelizumab to chemotherapy produced statistically significant and clinically meaningful 5-year OS benefits compared with chemotherapy alone in the first-line treatment of RM-NPC. These findings provided the first 5-year evidence supporting the benefit of PD-1–based chemoimmunotherapy in this setting.

**TRIAL REGISTRATION:**

ClinicalTrials.gov Identifier: NCT03707509

## Key Points

**Question:**

Will an immune checkpoint inhibitor plus chemotherapy improve 5-year overall survival as first-line treatment of patients with recurrent or metastatic nasopharyngeal carcinoma (RM-NPC)?

**Findings:**

In this secondary analysis of a randomized clinical trial including 263 randomized patients, the addition of camrelizumab to chemotherapy was associated with a statistically significant and clinically meaningful improvement in 5-year overall survival compared with chemotherapy alone. Rapid clearance of plasma Epstein-Barr virus DNA emerged as a valuable prognostic biomarker of long-term survival.

**Meaning:**

These findings provide the first 5-year evidence to inform clinical practice on programmed cell death 1 protein–based chemoimmunotherapy in RM-NPC, supporting camrelizumab plus chemotherapy as the standard first-line treatment and establishing a new benchmark for long-term survival in this population.

## Teaser

This secondary analysis of a randomized clinical trial examines the long-term survival benefits of adding camrelizumab vs placebo to chemotherapy for patients with recurrent or metastatic nasopharyngeal carcinoma in China.

## Introduction

Nasopharyngeal carcinoma (NPC) is a distinct epithelial malignant disease of the head and neck, with the highest prevalence in East and Southeast Asia.^1^ In these endemic regions, Epstein-Barr virus (EBV) infection is predominantly associated with the development of NPC.^1^ Globally, NPC accounts for approximately 120 000 new cases and 73 000 deaths each year.^2^ About 10% of patients present with synchronous metastases at diagnosis, and a further 20% to 30% develop distant relapse after chemoradiotherapy.^1,3,4^

For patients with recurrent or metastatic NPC (RM-NPC), 5 randomized phase 3 trials (CAPTAIN-1st, JUPITER-02, RATIONALE-309, AK105-304, and KL167-III-08) have consistently shown that programmed cell death 1 protein (PD-1) or programmed cell death 1 ligand 1 (PD-L1) inhibitors plus chemotherapy significantly improved progression-free survival (PFS) compared with placebo plus chemotherapy.^5,6,7,8,9^ Consequently, PD-1/PD-L1–based chemoimmunotherapy has been established as the standard first-line treatment for RM-NPC.^10,11,12^ However, only the JUPITER-02 and RATIONALE-309 studies have reported overall survival (OS) outcomes, with follow-up limited to 3 years.^13,14^ Given the long-term survival plateaus observed with immune checkpoint inhibitors in other malignant diseases,^15,16,17^ extended follow-up is essential to fully assess benefits. Whether immunotherapy confers durable benefits at the 5-year landmark in RM-NPC remains uncertain.

The CAPTAIN-1st trial^5^ was the first published, placebo-controlled, phase 3 trial to evaluate the PD-1 inhibitor camrelizumab in combination with chemotherapy in treatment-naive patients with RM-NPC, to our knowledge. At the prespecified cutoff on December 31, 2020, the study met its primary end point, demonstrating a statistically significant improvement in PFS with camrelizumab plus chemotherapy compared with placebo plus chemotherapy (hazard ratio [HR], 0.51; 95% CI, 0.37-0.69). OS data were immature at that time. Herein, we present the final 5-year survival outcomes from CAPTAIN-1st, representing the longest follow-up to date for randomized phase 3 trials of a PD-1 inhibitor plus chemotherapy in the first-line treatment of RM-NPC.

## Methods

### Study Design and Patients

The CAPTAIN-1st study was a randomized, double-blind, placebo-controlled, phase 3 trial conducted at 28 hospitals in China. The full protocol has been provided in a prior report and is available in Supplement 1.^5^ The protocol and all amendments were approved by the ethics committee at each participating site. The study was conducted in accordance with the Declaration of Helsinki and the Good Clinical Practice guidelines. All patients provided written informed consent. This trial is registered with ClinicalTrials.gov (NCT03707509).

Eligible patients were aged 18 to 75 years with pathologically confirmed nasopharyngeal carcinoma, either primary metastatic disease or recurrence not amenable to local treatment. Patients had not received previous systemic therapy for recurrent or metastatic disease; those who relapsed at least 6 months after induction, adjuvant, or concurrent chemoradiotherapy were eligible. Additional criteria included an Eastern Cooperative Oncology Group (ECOG) performance status of 0 or 1 and at least 1 measurable lesion per Response Evaluation Criteria in Solid Tumors (RECIST) version 1.1. At enrollment, disease staging was performed according to the *American Joint Committee on Cancer (AJCC) Tumor Node Metastases Staging System, Eighth Edition,* for nasopharyngeal carcinoma.

### Randomization and Treatment

Eligible patients were randomly assigned (1:1) to receive either camrelizumab plus chemotherapy (camrelizumab group) or placebo plus chemotherapy (placebo group). Randomization was stratified according to the presence of liver metastases (yes vs no), previous radical concurrent chemoradiotherapy (yes vs no), and ECOG performance status (0 vs 1). All investigators, patients, and funders were masked to treatment allocation. Camrelizumab and matching placebo were identical in packaging, labeling, appearance, and schedule of administration to preserve masking. The study remained blinded until completion of the prespecified primary PFS analysis, at which point treatment allocation was unmasked. Consequently, the current OS analysis was conducted in an open-label setting.

Patients intravenously received camrelizumab, 200 mg, or matching placebo intravenously on day 1, gemcitabine, 1000 mg/m^2^, on days 1 and 8, and cisplatin, 80 mg/m^2^, on day 1 of each 3-week cycle for 4 to 6 cycles, followed by maintenance camrelizumab or placebo every 3 weeks until radiographic progression, unacceptable toxic effects, new anticancer treatment, investigator decision, withdrawal of consent, or completion of 2 years of treatment. Crossover to camrelizumab at progression was not allowed in the placebo group. Survival status was assessed every 3 months after the last treatment dose.

### Study Assessments

Tumor response was assessed by an independent review committee and by investigators using computed tomographic (CT) imaging or magnetic resonance imaging (MRI) according to RECIST 1.1. Patients with disease progression who continued to derive clinical benefits, at the investigator’s discretion, could continue study treatment after sponsor consultation and renewed informed consent. Specifically, treatment beyond progression could be permitted only if the patient’s performance status had not markedly declined, there was no significant worsening of tumor-related symptoms, and no rapid progression or progression at critical anatomical sites (eg, spinal cord compression) requiring urgent intervention. For patients whose condition could not be clearly judged, the investigator was advised not to continue study medication beyond progression. Plasma EBV DNA level was measured at each hospital by real-time quantitative polymerase chain reaction at baseline and every 3 cycles thereafter, with copy numbers above the detection limit defined as positive results.

### End Points

The primary end point was independent review committee–assessed PFS, defined as the time from randomization to disease progression per independent review committee or death from any cause.^5^ A key secondary end point was OS, defined as time from randomization to death from any cause. Results of the interim (cutoff date June 15, 2020) and final (cutoff date December 31, 2020) analyses of PFS have been reported previously.^5^ We present the 5-year updated OS outcomes.

### Statistical Analysis

Efficacy analysis was conducted in all randomized patients who received at least 1 dose of study treatment (intention-to-treat population). The sample size estimation has been described previously,^5^ and no hierarchical testing or multiplicity adjustment for OS was prespecified. The median follow-up time was calculated by the reverse Kaplan-Meier method.^18^ This method considered the time until the last follow-up for censored or alive patients, and the median follow-up time was the point at which 50% of the patients have been censored or alive. Median OS was estimated by Kaplan-Meier method, with 95% CIs estimated by the Brookmeyer-Crowley method. Stratified log-rank tests were used to compare OS between groups, and HRs with 95% CIs were estimated by stratified Cox proportional hazards models. The stratification factors were identical to those used for randomization. Five-year OS rates and 95% CIs were estimated by Kaplan-Meier method with Greenwood formula. The proportional hazards assumption was tested using the Grambsch-Therneau test and examination of Schoenfeld residuals based on the stratified Cox model. Between-groups differences at the 5-year landmark were assessed with a Z test based on Greenwood standard errors. Subgroup analyses were performed using unstratified Cox proportional hazard models to assess the consistency of treatment effects across subgroups. A post hoc analysis was performed to evaluate the association of dynamic changes in plasma EBV DNA levels and OS. Baseline characteristics between 5-year or longer survivors and less than 5-year survivors were compared separately within the camrelizumab and placebo groups. Categorical variables were compared using the Pearson χ^2^ test. All tests were 2-sided with a nominal type I error (α) of 5%, without adjustment for multiplicity. All statistical analyses were performed in R statistical software (version 4.3.3; R Foundation, Inc). Data analysis was conducted at June 1, 2025.

## Results

### Patients and Treatment

**Figure 1.**
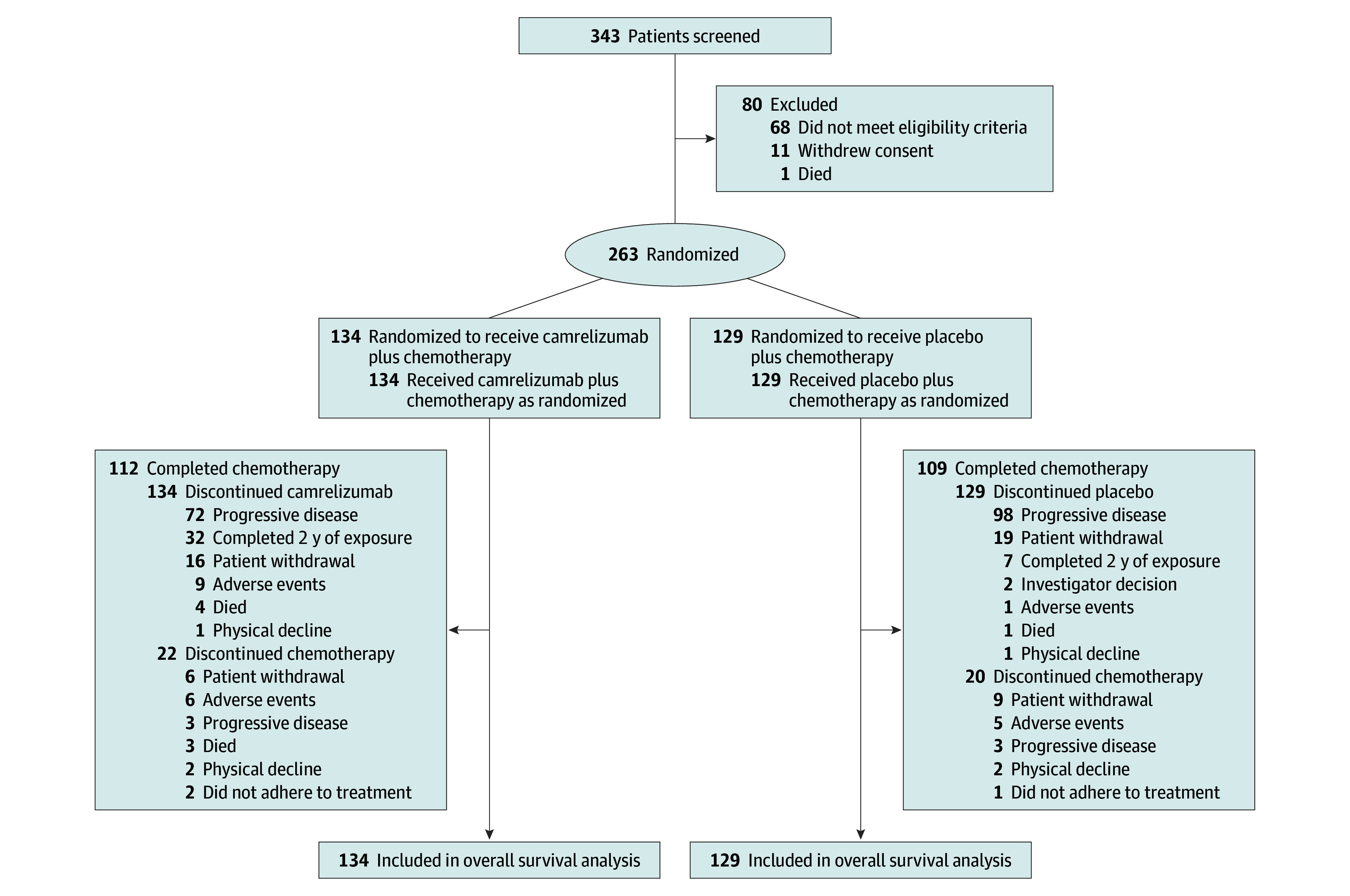
Trial Profile at the Final Analysis All patients who received at least 1 dose of camrelizumab/placebo, gemcitabine, or cisplatin were included in the analysis at data cutoff December 5, 2024.

Between November 13, 2018, and November 29, 2019, 343 patients were screened, and 263 eligible patients were randomly assigned to receive either camrelizumab (n = 134) or placebo (n = 129) in combination with gemcitabine and cisplatin (Figure 1; eTable 1 in Supplement 2). All enrolled patients received at least 1 dose of the study treatment and were included in the intention-to-treat population. Baseline characteristics were generally balanced between groups, except that 75 patients (56%) in the camrelizumab group were aged 50 years or older, compared with 56 (43%) in the placebo group (Table). All patients were Asian (self reported at study enrollment), and 216 (82%) had nonkeratinizing undifferentiated histologic findings. Overall, 169 patients (64%) had received concurrent chemoradiotherapy, and all patients had distant metastases.

**Table.** Baseline Demographics and Disease Characteristics in Intent-to-Treat Population

Characteristic	No. (%)	
	Camrelizumab group (n = 134)	Placebo group (n = 129)
Age, y		
Median (IQR)	52 (40-58)	49 (40-56)
<50	59 (44)	73 (57)
≥50	75 (56)	56 (43)
Sex		
Female	21 (16)	24 (19)
Male	113 (84)	105 (81)
Ethnicity		
Asian	134 (100)	129 (100)
ECOG performance status		
0	47 (35)	44 (34)
1	87 (65)	85 (66)
Baseline plasma EBV DNA level		
Positive	95 (71)	86 (67)
Negative	39 (29)	43 (33)
WHO classification		
Keratinizing	1 (<1)	1 (<1)
Nonkeratinizing differentiated	21 (16)	21 (16)
Nonkeratinizing undifferentiated	110 (82)	106 (82)
Other	2 (1)	1 (<1)
Baseline metastatic site		
Liver	70 (52)	66 (51)
Lung	66 (49)	61 (47)
Concurrent chemoradiotherapy history		
Yes	86 (64)	83 (64)
No	48 (36)	46 (36)
No. of metastatic organs		
1	44 (33)	48 (37)
2	56 (42)	42 (33)
≥3	34 (25)	39 (30)
Disease status		
Primary metastatic	47 (35)	42 (33)
Recurrent with distal metastases	87 (65)	87 (67)

Abbreviations: ECOG, Eastern Cooperative Oncology Group; EBV, Epstein-Barr virus; WHO, World Health Organization.

At the data cutoff on December 5, 2024, the median follow-up was 63.5 (95% CI, 61.2-64.6) months in the camrelizumab group and 63.0 (95% CI, 60.8-64.6) months in the placebo group. The median (IQR) treatment duration was 12.8 (6.9-22.8) months with camrelizumab and 8.2 (5.9-12.6) months with placebo, with median (IQR) treatment cycles of 16.0 (9.0-30.0) and 10.0 (8.0–17.0), respectively (eTable 2 in Supplement 2). A total of 92 patients (69%) in the camrelizumab group and 85 patients (66%) in the placebo group received the planned 6 cycles of chemotherapy, whereas 27 (20%) and 29 patients (22%) received 4 or fewer cycles, respectively. Subsequent anticancer therapies after study treatment were administered to 82 patients (61.2%) in the camrelizumab group and 97 patients (75.2%) in the placebo group (eTable 3-4 in Supplement 2). The most common therapies were systemic chemotherapy (68 [50.7%] vs 90 [69.8%]), followed by PD-1 or PD-L1 inhibitors (40 [29.9%] vs 42 [32.6%]). Due to the time gap between study initiation and approval of postline anti–PD-1/PD-L1 therapy, some patients in the placebo group did not receive such agents in the later stages. The distribution of other poststudy therapies was generally comparable between groups.

### OS

**Figure 2.**
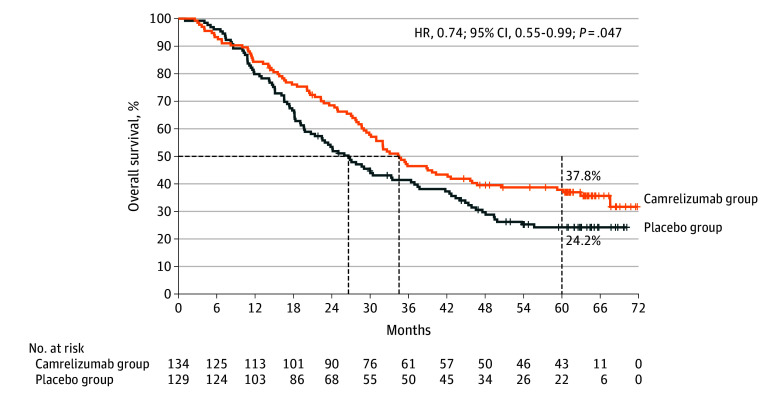
Overall Survival (OS) in the Intention-to-Treat Population Kaplan-Meier curves for OS at data cutoff at December 5, 2024. The hazard ratio (HR) was stratified by liver metastases, previous radical chemoradiotherapy, and Eastern Cooperative Oncology Group performance status. The dashed lines indicate median OS (50%) and 60-month OS.

In the post hoc analysis, death events occurred in 85 patients (63.4%) in the camrelizumab group and 95 patients (73.6%) in the placebo group. Patients were censored due to being alive (40 [29.9%] vs 22 [17.1%]) or loss to follow-up (9 [6.7%] vs 12 [9.3%]), respectively. Median OS was 34.5 (95% CI, 29.4-45.7) months with camrelizumab and 26.6 (95% CI, 19.8-33.5) months with placebo (stratified HR, 0.74; 95% CI, 0.55-0.99; *P* = .047; Figure 2). According to the Grambsch-Therneau test and examination of Schoenfeld residuals (eFigure 1 in Supplement 2), the proportional hazards assumption was not violated. The 5-year OS rates were 37.8% (95% CI, 29.6%-46.1%) in the camrelizumab group vs 24.2% (95% CI, 17.0%-32.2%) in the placebo group, reflecting an absolute benefit of 13.6% (95% CI, 2.4%-24.8%; *P* = .02).

**Figure 3.**
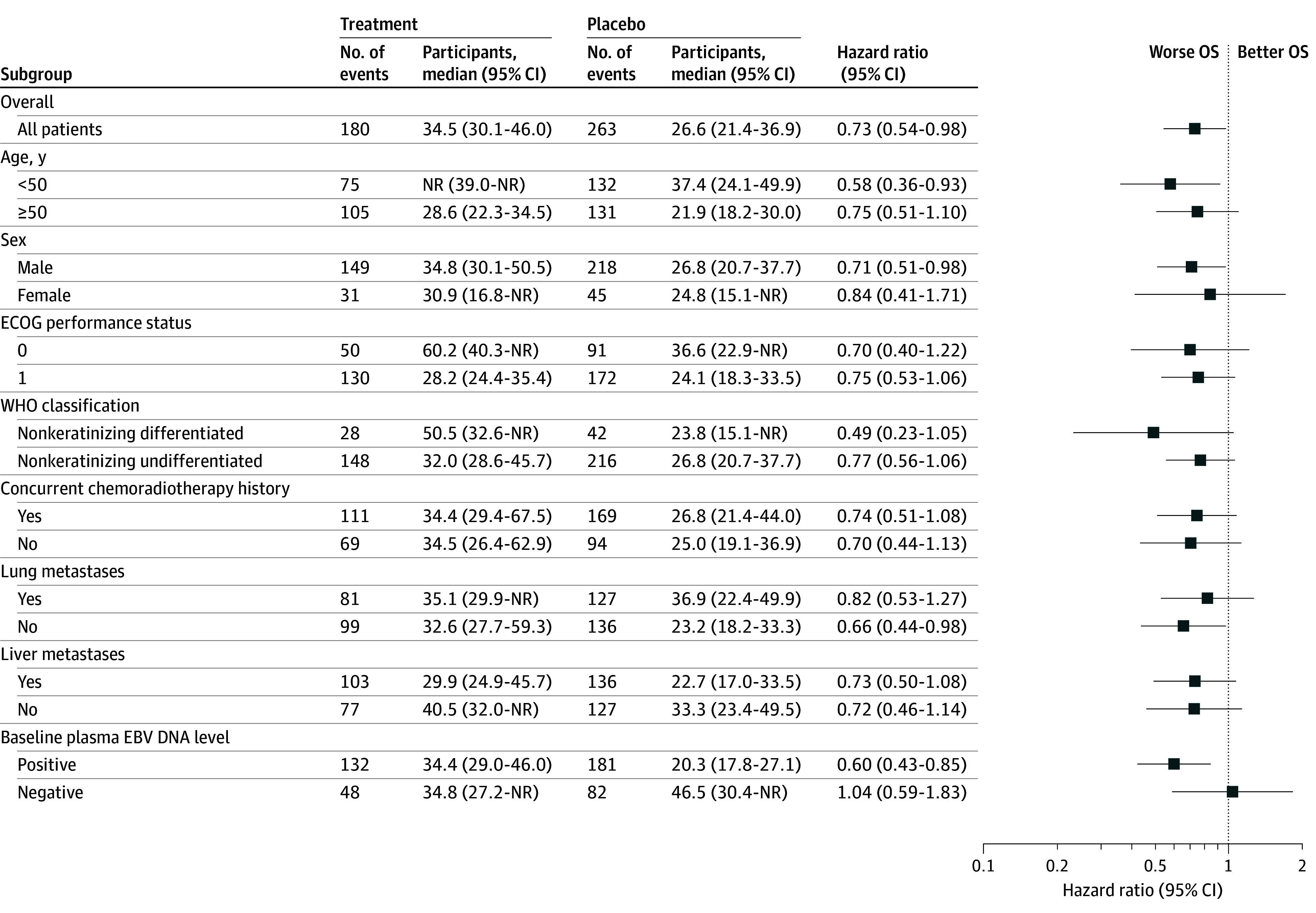
Subgroup Analysis of Overall Survival (OS) Hazard ratios (HRs) and 95% CIs were estimated with unstratified Cox proportional hazards models. EBV indicates Epstein-Barr virus; ECOG, Eastern Cooperative Oncology Group; NR, not reached; WHO, World Health Organization.

Subgroup analyses showed generally consistent treatment effects favoring camrelizumab plus chemotherapy across most subgroups, except for patients with negative baseline EBV DNA (HR, 1.04; 95% CI, 0.59-1.83) (Figure 3). Furthermore, age (≤50 vs >50 years) was a strong prognostic factor in both treatment groups (eFigure 2 in Supplement 2). Considering the age imbalances between the 2 treatment groups, we conducted a multivariable Cox regression model with age as a covariate, yielding the adjusted HR of 0.65 (95% CI, 0.48-0.89; *P* = .006).

### EBV DNA Assessments

**Figure 4.**
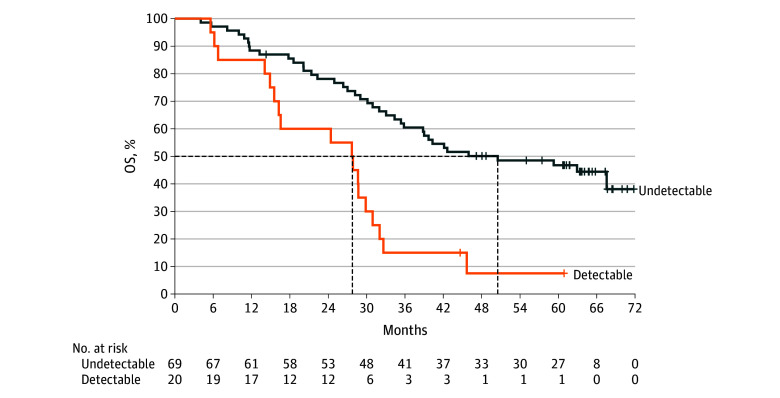
Overall Survival (OS) by Plasma Epstein-Barr Virus (EBV) DNA Clearance in the Camrelizumab Group Kaplan-Meier curves for OS in the camrelizumab group comparing patients who achieved plasma EBV DNA clearance in the first 3 treatment cycles vs those with persistently positive EBV DNA. The dashed lines indicate median OS and the corresponding time point.

Dynamic changes in plasma EBV DNA were monitored for their association with OS (eFigure 3 in Supplement 2). Among patients with positive EBV DNA at baseline, a reduction in EBV copy number was observed in 88 of 89 patients (98.9%) in the camrelizumab group and 78 of 80 patients (97.5%) in the placebo group. Rapid EBV DNA clearance, defined as a change from positive at baseline to negative within the first 3 treatment cycles, occurred in 69 of 89 patients (77.5%) with camrelizumab and 53 of 80 patients (66.3%) with placebo. In the camrelizumab group, patients who achieved rapid clearance had a median OS of 50.5 (95% CI, 35.4-not reached [NR]) months and a 5-year OS rate of 46.8% (95% CI, 34.5%-58.1%), compared with 27.8 (95% CI, 14.9-31.0) months and 7.5% (95% CI, 0.6%-26.6%) among those without rapid clearance (HR, 0.32; 95% CI, 0.18-0.58; *P* < .001; Figure 4). Similar prognostic associations were observed in the placebo group (eFigure 4 in Supplement 2). Importantly, irrespective of clearance status, camrelizumab conferred significant OS benefits over placebo (eFigure 5 in Supplement 2).

### Five-Year Survivors

Baseline characteristics of patients who survived 5 years or longer in the camrelizumab group (n = 43 [32.1%]) and placebo group (n = 22 [17.1%]) were compared with those who survived fewer than 5 years, showing significant differences in age and EBV DNA clearance in the camrelizumab group, and in ECOG performance status and number of metastatic organs in the placebo group (eTable 5 in Supplement 2). Notably, 63 of 65 (96.9%) 5-year survivors had either negative baseline EBV DNA or achieved clearance within the first 3 treatment cycles. Among the 43 (32.1%) 5-year survivors in the camrelizumab group, 25 patients (58.1%) completed 2 years of camrelizumab, 24 patients (55.8%) received subsequent anticancer therapy, and 14 patients (32.6%) were alive without progression or subsequent treatment. The median (IQR) treatment duration for camrelizumab was 23.6 (16.6-23.9) months, corresponding to a median (IQR) of 32.0 (21.5-34.0) cycles. In the placebo group, 4 of 22 survivors (18.2%) completed 2 years of placebo, 19 survivors (86.4%) received subsequent therapy, and 3 survivors (13.6%) were alive without progression or subsequent therapy. The median (IQR) treatment duration was 13.5 (9.0-23.0) months, corresponding to 18.5 (12.0-31.3) cycles.

### Patients Completing 2 Years of Camrelizumab

Among patients randomly assigned to camrelizumab plus chemotherapy, 32 (23.9%) completed the planned 2 years of camrelizumab, 76 (56.7%) discontinued due to disease progression or death, and 26 (19.4%) discontinued for other reasons (adverse event, patient withdrawal, physical decline, or investigator decision). Median OS was not reached (95% CI, NR-NR) in patients completing 2 years of camrelizumab, with a 5-year OS rate of 87.1% (95% CI, 69.2%-95.0%), compared with 28.2 (95% CI, 22.3-32.0) months and 18.9% (95% CI, 10.8%-28.6%) in those discontinuing due to progression or death, and 27.0 (95% CI, 16.8-46.7) months and 30.8% (95% CI, 14.6%-38.5%) in those for other discontinuations (eFigure 6 in Supplement 2).

## Discussion

This 5-year analysis of the CAPTAIN-1st randomized clinical trial demonstrated that camrelizumab plus chemotherapy provided statistically significant and clinically meaningful OS benefits compared with chemotherapy alone in the first-line treatment of recurrent or metastatic nasopharyngeal carcinoma (RM-NPC). The addition of camrelizumab to gemcitabine and cisplatin reduced the risk of death by 26% and increased the absolute 5-year survival rate by 13.6%. To our knowledge, this is the first randomized, phase 3 study to show a 5-year OS benefit with a PD-1 inhibitor combined with chemotherapy in this setting.

Five-year survival has been widely recognized as a benchmark of cancer treatment effectiveness for more than 6 decades.^19^ In the era of immunotherapy, long-term survival is particularly meaningful because survival plateaus have been observed in several malignant diseases, including advanced melanoma and non–small cell lung cancer, indicating durable benefits in a subset of patients.^15,16,17^ For RM-NPC, PD-1/PD-L1 inhibitors combined with chemotherapy have been established as the first-line standard of care, but 5-year survival outcomes had not been reported.^13,14^ In this report from the CAPTAIN-1st randomized clinical trial, with 63.4 months of follow-up and 68% OS maturity, camrelizumab plus chemotherapy significantly improved 5-year OS (HR, 0.74; 95% CI, 0.55-0.99), with further strengthening after adjustment for age imbalance (HR, 0.65; 95% CI, 0.48-0.89). A survival plateau was observed in the camrelizumab group, with a 5-year OS rate of 37.8%, suggesting that about one-third of patients achieved sustained long-term survival. These findings provide pivotal 5-year evidence supporting PD-1–based chemoimmunotherapy in RM-NPC.

Dynamic changes in plasma EBV DNA are closely associated with clinical outcomes after chemotherapy and radiotherapy.^20,21,22^ Increasing evidence also support their prognostic value in immunotherapy.^5,23,24^ However, whether EBV DNA kinetics are associated with long-term outcomes with PD-1/PD-L1–based chemoimmunotherapy remained uncertain. Our extended follow-up of the CAPTAIN-1st trial now provides the first 5-year evidence that rapid plasma EBV DNA clearance is a robust prognostic biomarker for both the camrelizumab and placebo groups. In both treatment groups, patients who achieved rapid EBV DNA clearance had a median OS nearly doubled vs those without rapid clearance, and their 5-year OS rate was approximately 6-fold higher. Notably, almost all 5-year survivors had either baseline-negative EBV DNA or achieved rapid clearance. Given these findings, longitudinal monitoring of plasma EBV DNA should be incorporated into clinical practice to guide individualized treatment. For patients who do not achieve rapid clearance, alternative strategies, such as antibody-drug conjugates or novel immune checkpoint inhibitors, might be warranted.^25,26,27^ In this context, 2 ongoing umbrella trials in locoregionally-advanced NPC, EP-STAR, and RIBBON-UM, are prospectively evaluating EBV DNA during induction chemotherapy to identify patients at high risk of relapse and guide real-time treatment adaptation.^28,29^

Several characteristics were enriched among 5-year survivors, such as being younger than 50 years, ECOG performance status of 0, negative baseline plasma EBV DNA, and rapid EBV DNA clearance. Nevertheless, camrelizumab plus chemotherapy showed generally consistent OS benefits over chemotherapy alone across most subgroups, including age, ECOG status, and presence of liver metastases. In addition, benefits of camrelizumab were observed irrespective of EBV DNA clearance status. The exception was the subgroup with negative baseline plasma EBV DNA (HR, 1.04; 95% CI, 0.59-1.83). This result should be interpreted with caution, because a greater proportion of patients in the placebo group received subsequent anticancer treatment vs the camrelizumab group (87/129 [67.4%] vs 62/134 [46.2%]).

### Limitations

This study had several limitations. First, all participants were enrolled from NPC-endemic East-Asian regions, where nonkeratinizing carcinoma predominates. Therefore, the benefits of camrelizumab plus chemotherapy in nonendemic regions warrant further validation. Second, patients who completed 2 years of camrelizumab demonstrated markedly better survival than those who discontinued treatment. However, this finding may be confounded by survivorship bias because patients who lived longer were naturally more likely to receive extended treatment. Given the potential bias, these results should be interpreted with caution, and the association between longer treatment duration and improved survival does not imply causality. Finally, although OS was a key secondary end point, no multiplicity adjustment was performed for OS analysis. Nonetheless, the OS results still provided meaningful evidence of the long-term survival benefits of camrelizumab plus chemotherapy as first-line treatment of RM-NPC.

## Conclusions

The randomized, phase 3 CAPTAIN-1st study provides the first 5-year evidence, to our knowledge, that the addition of a PD-1 inhibitor (camrelizumab) to gemcitabine and cisplatin confered significant survival benefits in the first-line treatment of RM-NPC. These results reinforce camrelizumab plus chemotherapy as the standard of care and establish a new benchmark for long-term survival in this population.

## Notes

Supplement 1. Trial Protocol

Supplement 2. eTable 1. Patient disposition by site eTable 2. Summary of study drug exposure at data cutoff (Dec 5, 2024) eTable 3. Subsequent anti-cancer therapies in intent-to-treat population after the study treatment eTable 4. Subsequent anti-cancer therapies in EBV DNA negative population after the study treatment eTable 5. Baseline demographics and disease characteristics of ≥ 5-year and < 5-year survivors eFigure 1. The examination of Schoenfeld residuals from stratified Cox proportional regression model for overall survival eFigure 2. Overall survival by age in the camrelizumab and placebo groups eFigure 3. Dynamic changes in plasma EBV DNA copy numbers during two years of treatment eFigure 4. Overall survival by plasma EBV DNA clearance in the placebo group eFigure 5. Overall survival by plasma EBV DNA clearance between camrelizumab and placebo groups eFigure 6. Overall survival by completion or discontinuation of 2 years of camrelizumab treatment

Supplement 3. Data Sharing Statement
