# Supplementary material for: Five-Year Outcome of Camrelizumab Plus Chemotherapy in Recurrent or Metastatic Nasopharyngeal Carcinoma: A Secondary Analysis of the CAPTAIN-1st Randomized Clinical Trial
Source: JAMA Oncol. 2026 Jan 29;12(3):295–302. doi: 10.1001/jamaoncol.2025.6245 (PMC12856745; doi:10.1001/jamaoncol.2025.6245)
Supplement: Supplement 1. — Trial Protocol [file jamaoncol-e256245-s001.pdf]

A randomized, double-blind, placebo-controlled, multi-center, Phase III clinical study of camrelizumab combined with cisplatin and gemcitabine versus placebo combined with cisplatin and gemcitabine in the treatment of locally advanced relapsed or metastatic nasopharyngeal carcinoma

Protocol number: SHR-1210-III-308

Trial phasing: Phase III

Compound number: SHR-1210

Compound name: Camrelizumab

Protocol head: Yang Qing

Clinical trial group leader organization: Sun Yat-sen University Cancer Center

Principal investigator: Zhang Li

Version number: 4.0

Version date: September 16, 2020

Sponsor: Jiangsu Hengrui Medicine Co., Ltd.

No. 7, Kunlunshan Road, Lianyungang Economic and Technological Development Zone, Jiangsu Province 222047

Confidentiality Statement

The information contained in this protocol is confidential and only for use by clinical investigators, and shall not be disclosed unless required by currently effective laws or regulations. Its copyright is owned by Jiangsu Hengrui Medicine Co., Ltd. or its subsidiaries. Except for those who have entered into a confidentiality agreement with Jiangsu Hengrui Medicine Co., Ltd. or its subsidiaries, the protocol shall not be copied or distributed to any persons not participating in the clinical study.

Protocol Signature Page for the Sponsor

We have read and confirmed the clinical trial protocol (study number: SHR-1210- III-308, Version number: 4.0, Version date: September 16, 2020). We agree to fulfill relevant responsibilities in accordance with Chinese laws, Declaration of Helsinki, Chinese Good Clinical Practice (GCP) and this study protocol.

Sponsor: _Jiangsu Hengrui Medicine Co., Ltd._________

Yang Qing		
Study director (print form)	Study director (signature)	Signing date (mm/dd/yyyy)

Protocol Signature Page for the Principal Investigator (Group Leader Organization)

I will earnestly fulfill the investigator's responsibilities in accordance with the Chinese GCP provisions, and personally participate in or directly guide this clinical study. I have received the Investigator's Brochure for the study drug in this clinical trial; I am aware and have read the preclinical study findings of the investigational drug and the clinical trial's study protocol. I agree to fulfill relevant responsibilities in accordance with Chinese laws, Declaration of Helsinki, Chinese GCP and the study protocol. Unless measures must be taken to protect the subjects' safety, rights and interests, I will only make revisions to the protocol after notifying and obtaining consent from the sponsor, and implement revisions after obtaining consent from the Ethics Committee. I will be responsible for making clinically relevant medical decisions, ensure that subjects can access timely and appropriate treatment when experiencing adverse events during the study period, and record and report these adverse events as required by relevant national provisions. I undertake to truthfully, accurately, completely and timely record data. I will accept the monitoring and audits by monitors or auditors dispatched by the sponsor and inspection by drug supervision and administration departments, and ensure the quality of the clinical trial. I undertake to keep the personal information of subjects and relevant matters confidential. I agree to publicize my full name and occupation to the sponsor, agree to publicize clinical study-related expenditures as required, and agree to prohibit commercial and economic behaviors related to the trial. I agree that the study results can be used for drug registration and publicly published. I will provide a resume of the principal investigator before the start of the study, and submit it to the Ethics Committee and to the drug supervision and administration departments for filing.

Study organization:____________________________________

______________________	______________________	______________________
Principal investigator (print form)	Principal investigator (signature)	Signing date (mm/dd/yyyy)

Protocol Signature Page for the Principal Investigator (Participating Organizations)

I will earnestly fulfill the investigator's responsibilities in accordance with the Chinese GCP provisions, and personally participate in or directly guide this clinical study. I have received the Investigator's Brochure for the study drug in this clinical trial; I am aware and have read the preclinical study findings of the investigational drug and the clinical trial's study protocol. I agree to fulfill relevant responsibilities in accordance with Chinese laws, Declaration of Helsinki, Chinese GCP and the study protocol. Unless measures must be taken to protect the subjects' safety, rights and interests, I will only make revisions to the protocol after notifying and obtaining consent from the sponsor, and implement revisions after obtaining consent from the Ethics Committee. I will be responsible for making clinically relevant medical decisions, ensure that subjects can access timely and appropriate treatment when experiencing adverse events during the study period, and record and report these adverse events as required by relevant national provisions. I undertake to truthfully, accurately, completely and timely record data. I will accept the monitoring and audits by monitors or auditors dispatched by the sponsor and inspection by drug supervision and administration departments, and ensure the quality of the clinical trial. I undertake to keep the personal information of subjects and relevant matters confidential. I agree to publicize my full name and occupation to the sponsor, agree to publicize clinical study-related expenditures as required, and agree to prohibit commercial and economic behaviors related to the trial. I agree that the study results can be used for drug registration and publicly published. I will provide a resume of the principal investigator before the start of the study, and submit it to the Ethics Committee and to the drug supervision and administration departments for filing.

Study organization:____________________________________

________________________	________________________	________________________
Principal investigator (print form)	Principal investigator (signature)	Signing date (mm/dd/yyyy)

Table of Contents

Table of Contents....1
Directory of Tables....4
Directory of Figures....4
Protocol Synopsis....5
Trial Flowchart....13
Abbreviations....18
1 Introduction: Study Background and Scientific Rationale....20
1.1 Study Background....20
1.1.1 Nasopharyngeal Carcinoma....20
1.1.2 Camrelizumab....20
1.2 Scientific Basis....23
1.2.1 Basis of Study....23
1.2.2 Rationale for Design of the Dosing Regimen....24
1.3 Potential Risks and Benefits....24
1.3.1 Known Potential Risks....24
1.3.2 Known Possible Benefits....25
2 Study Objectives and Study Endpoints....25
2.1 Study Objectives....25
2.1.1 Primary Study Objective....25
2.1.2 Secondary study objectives....25
2.1.3 Exploratory study objective....25
2.2 Study Endpoints....25
2.2.1 Primary study endpoint....25
2.2.2 Secondary study endpoints....26
2.2.3 Exploratory study endpoint....26
3 Study Design....26
3.1 Overall Design....26
3.2 Methods to Reduce Bias....29
3.2.1 Enrollment/Randomization/Blinding Steps....29
3.2.2 Blind Evaluation....29
3.2.3 Unblinding....29
4 Subject Selection and Withdrawal....30
4.1 Inclusion Criteria....30
4.2 Exclusion Criteria....31
4.3 Randomization Criteria....33
4.4 Lifestyle Requirements....33
4.4.1 Contraception....33
4.5 Subjects' Withdrawal from the Study or Termination of Study Treatment....33
4.5.1 Criteria for Withdrawal from the Study....33
4.5.2 Criteria for Termination of Study Treatment....33
4.5.3 Steps for Withdrawal from or Termination of Treatment....34

4.6 Early Termination or Suspension of the Study 34
4.7 Definition of End of Trial 35
5 Immunogenicity Studies 35
5.1 Collection and Processing of Blood Samples 35
5.1.1 Time of Collection of Blood Samples 35
5.1.2 Processing and Preservation of Blood Samples 35
5.2 Mailing of Clinical Samples 35
6 Study Medication 35
6.1 Overview of Test Drug and Medication for the Control Group 35
6.1.1 Method of Drug Acquisition 35
6.1.2 Dosage Form, Appearance and Package of Drugs 35
6.1.3 Drug Preservation and Stability 37
6.1.4 Drug Preparation 37
6.1.5 Drug Usage 37
6.1.6 Dose Adjustment and Delay 39
6.2 Drug Management, Distribution and Recovery 43
6.2.1 Destruction of Study Drug 43
6.3 Accompanying Treatments 43
6.3.1 Allowed Concomitant Treatments 43
6.3.2 Banned Concomitant Treatments 44
6.3.3 Supportive Care 44
6.3.4 Hematopoietic Growth Factors 47
7 Study Steps 48
7.1 Screening 48
7.2 Trial Period 49
7.3 Withdrawal from Study Treatment Visit 50
7.4 Safety Follow-up 51
7.5 Unscheduled Visit 52
7.6 Survival Follow-up 52
7.7 Tumor Progression Follow-up 52
8 Evaluation 52
8.1 Effectiveness Evaluation 52
8.2 Safety Evaluation 54
8.2.1 Pregnancy Test 54
8.2.2 Adverse Events 54
8.2.3 Laboratory Safety Evaluation 54
8.2.4 Vital Signs and Physical Examination 54
8.2.5 12-lead ECG 54
8.3 Independent Data Monitoring Committee 54
9 Reporting of Adverse Events 55
9.1 Adverse Event (AE) 55
9.1.1 Definition of Adverse Event 55
9.1.2 Criteria for Judgment of AE Severity 55
9.1.3 Judgment of Relationship between AE and Test Drug 56

9.2 Serious Adverse Event (SAE) 56
9.2.1 Definition of SAE 56
9.2.2 Hospitalization 56
9.2.3 Progressive Disease 57
9.2.4 Abnormal Liver Enzymes 57
9.2.5 SAE Reporting System 59
9.2.6 AE/SAE Follow-up 59
9.3 Pregnancy 60
9.4 AE of Special Interest 60
10 Clinical Monitoring 60
11 Data Analysis/Statistical Method 61
11.1 Sample Size 61
11.2 Analysis Data Sets 61
11.3 Statistical Analysis 62
11.3.1 Basic Method 62
11.3.2 Primary Effectiveness Endpoint Analysis 62
11.3.3 Secondary Effectiveness Endpoint Analysis 62
11.3.4 Processing of Missing Data 62
11.3.5 Safety Analysis 62
11.3.6 Interim Analysis 63
11.3.7 Subgroup Analysis 63
11.3.8 Exploratory Analysis 64
12 Data Management Method 64
12.1 Data Recording 64
12.1.1 eCRF Filling 64
12.1.2 eCRF Review 64
12.2 Data Monitoring 64
12.3 Data Management 64
12.3.1 Establishment of EDC Database 64
12.3.2 Data Entry and Verification 65
12.3.3 Data Review and Database Locking 65
12.3.4 Data Archiving 65
13 Source Data and Original Documents 65
14 Quality Assurance and Quality Control 65
15 Regulatory Ethics, Informed Consent and Subject Protection 66
15.1 Regulatory Considerations 66
15.2 Ethical Norms 66
15.3 Independent Ethics Committee 67
15.4 Informed Consent 67
15.4.1 Informed Consent Form and Other Written Information Required for Subjects 67
15.4.2 Informed Consent Process and Record 67
15.5 Confidentiality of Subject Information 67
15.5.1 Use of Samples, Specimens or Data for Research Purposes 68
15.5.2 Future Use of Preserved Specimens 68

16 Publication of Study Results....68
17 Key Functions and Roles....68
18 Clinical Study Progress....68
19 References....70
Annex I ECOG Performance Status Scoring Criteria....71
Annex II Calculation of Creatinine Clearance....72
Annex III AJCC Cancer Staging System: 8th Edition (Excerpt) *....73
Annex IV Response Evaluation Criteria in Solid Tumors....75
Annex V Chinese Medicines Prohibited in the Trial Period....85

Directory of Tables

Table 1 Treatment Grouping of Subjects....26
Table 2 Method of Administration....38
Table 3 Criteria for Confirmation of PD....39
Table 4 Criteria for Dose Adjustment of Camrelizumab/Placebo....39
Table 5 Dose Adjustment Levels for Chemical Drugs....41
Table 6 Dose Adjustment of Chemical Drugs Based on the Chemotherapy-related Hematological Toxicities of the Previous Cycle....41
Table 7 Dose Adjustment of Chemical Drugs Based on the Chemotherapy-related Non-hematological Toxicities of the Previous Cycle....42
Table 8 Recommendations for the Treatment ofInfusion Reactions....46

Directory of Figures

Figure 1 Study Design Diagram....28

Protocol Synopsis

**Study title**	A randomized, double-blind, placebo-controlled, multi-center, Phase III clinical study of camrelizumab combined with cisplatin and gemcitabine versus placebo combined with cisplatin and gemcitabine in the treatment of locally advanced relapsed or metastatic nasopharyngeal carcinoma
**Protocol number**	SHR-1210-III-308
**Version number**	4.0
**Sponsor**	Jiangsu Hengrui Medicine Co., Ltd.
**Principal investigator**	Professor Zhang Li
**Participating sites**	More than 30 sites including Sun Yat-sen University Cancer Center
**Study objectives**	**Primary study objective**<br>• To compare the progression-free survival (PFS) (evaluated by the Independent Review Committee [IRC]) of camrelizumab combined with cisplatin and gemcitabine versus placebo combined with cisplatin and gemcitabine in the treatment of locally advanced relapsed or metastatic nasopharyngeal carcinoma<br>**Secondary study objectives**<br>• To compare the PFS (evaluated by the investigator), overall survival (OS), objective response rate (ORR) and disease control rate (DCR) of camrelizumab combined with cisplatin and gemcitabine versus placebo combined with cisplatin and gemcitabine in the treatment of locally advanced relapsed or metastatic nasopharyngeal carcinoma, and evaluate the duration of response (DoR) in the two groups<br>• To evaluate the safety of camrelizumab combined with cisplatin and gemcitabine versus placebo combined with cisplatin and gemcitabine in the treatment of locally advanced relapsed or metastatic nasopharyngeal carcinoma<br>**Exploratory study objective**<br>• To test the proportion of occurrence of camrelizumab antibodies
**Study endpoints**	**Primary study endpoint**<br>• IRC-assessed PFS;<br>**Secondary study endpoints**<br>• Investigator-assessed PFS;<br>• ORR;<br>• DCR;<br>• DoR;<br>• OS;<br>• Safety: adverse event (AE), laboratory tests, vital signs, ECG, physical examination.<br>**Exploratory study endpoint**<br>• To test of the proportion of occurrence of camrelizumab antibodies

**Study subjects**	Patients with unresectable locally advanced/relapsed or distantly metastatic nasopharyngeal carcinoma who have not previously received systemic anti-tumor treatment
**Study design**	The study uses a randomized, double-blind, placebo-controlled, multi-center study design, and chooses patients with unresectable locally advanced/relapsed or distantly metastatic nasopharyngeal carcinoma who have not previously received systemic anti-tumor treatment. The study plans to enroll 250 cases. Eligible subjects will be 1:1 randomized into test group or control group. Stratification factors include liver metastasis (yes vs. no), whether the subject has received radical concurrent radiochemotherapy (yes vs. no) and ECOG score (0 vs. 1).<br>The test group receives camrelizumab (200 mg, D1), cisplatin (80 mg/m^2, D1) and gemcitabine (1000 mg/m^2, D1, D8). With 3 weeks as 1 dosing cycle, the combination chemotherapy is given for 4-6 cycles, because of no less than 3 cycles in subjects with intolerable toxicity.<br>The control group receives placebo (D1), cisplatin (80 mg/m^2, D1) and gemcitabine (1000 mg/m^2, D1, D8). With 3 weeks as 1 dosing cycle, the combination chemotherapy is given for 4-6 cycles, because of no less than 3 cycles in subjects with intolerable toxicity..<br>After the end of chemotherapy, subjects are given camrelizumab (200 mg, D1)/placebo (D1) for maintenance monotherapy until progressive disease, intolerable toxicity, withdrawal of informed consent or the investigator judges that the subject needs to withdraw from the study treatment. The longest medication duration of camrelizumab/placebo is 2 years.<br>Subjects can be unblinded after PD is confirmed by the IRC, except for the following three types of subjects who need to remain blind:<br>1. Subjects who are receiving study drug treatment and have not had IRC-confirmed disease progression;<br>2. Subjects who have stopped treatment and are still undergoing tumor progression follow-up, and have not had IRC-confirmed disease progression;;<br>3. Subjects who are lost to follow-up.<br>The screening period of the study is 28 days. Subjects who complete the screening examinations and assessments and meet the inclusion/exclusion criteria enter the treatment period to receive treatment according to the dosing frequency specified by the protocol, and complete relevant examinations and assessments before each administration. Imaging tumor evaluation is performed every 6 weeks (±7 days). Then imaging examination is performed every 12 weeks (±7 days) since 16 months after the first dose. All subjects complete safety examinations and imaging assessments when withdrawing from the treatment, and then enter the safety follow-up period for a total of 90 days. After withdrawing from the treatment, subjects start survival follow-up once every 3 months since the last dose. Subjects removed for non-PD reasons should receive tumor progression follow-up, and imaging assessment should be continued at the frequency of every 6 weeks (±7 days), and every 12 weeks (±7 days) since 16 months after the first dose until PD, initiation of a new anti-tumor treatment, withdrawal of informed consent, loss to follow-up or death.

**Study drug**	Recombinant humanized anti-PD-1 monoclonal antibody camrelizumab for injection;<br>Camrelizumab placebo;<br>Gemcitabine;<br>Cisplatin.

**Mode of administration**	**Test group:** every 3 weeks as 1 dosing cycle.<br>Camrelizumab: 200 mg/time, intravenous infusion over 30 min (no less than 20 min and no more than 60 min), administered on Day 1 of each cycle;<br>Cisplatin: 80 mg/m^2, administered on Day 1 of each cycle;<br>Gemcitabine: 1000 mg/m^2, administered on Days 1 and 8 of each cycle;<br>The combination chemotherapy can be given for 4-6 cycles, because of no less than 3 cycles in subjects with intolerable toxicity., and after withdrawal, subjects can receive maintenance treatment with camrelizumab. With every 3 weeks is 1 dosing cycle, the drug is administered on Day 1 of each cycle.<br>**Control group:** every 3 weeks as 1 dosing cycle.<br>Camrelizumab placebo: intravenous infusion over 30 min (no less than 20 min and no more than 60 min), administered on Day 1 of each cycle;<br>Cisplatin: 80 mg/m^2, administered on Day 1 of each cycle;<br>Gemcitabine: 1000 mg/m^2, administered on Days 1 and 8 of each cycle;<br>The combination chemotherapy can be given for 4-6 cycles, because of no less than 3 cycles in subjects with intolerable toxicity, and after withdrawal, subjects can receive maintenance treatment with camrelizumab placebo. With every 3 weeks is 1 dosing cycle, the drug is administered on Day 1 of each cycle.
**Inclusion criteria**	**Subjects must meet all of the following inclusion criteria to be eligible for the study.**<br>1. Aged ≥ 18 years and ≤ 75 years, male or female;<br>2. Patients pathologically diagnosed with nasopharyngeal carcinoma;<br>3. Patients newly diagnosed with advanced nasopharyngeal carcinoma at Stage IVb defined by the American Journal of Critical Care (AJCC) staging system (the 8th edition), or relapsed nasopharyngeal carcinoma not suitable for local treatment. Local treatment mainly refers to anti-tumor treatment-related measures, including surgery, radiofrequency ablation, transcatheter arterial chemoembolization (TACE), radiotherapy (except for radiotherapy at local appropriate doses for the relief of symptoms that does not affect blood pictures in patients with bone metastases);<br>4. Patients who have not received chemotherapy for relapsed or metastatic nasopharyngeal carcinoma (except for patients whose disease progressed more than 6 months after the receipt of neoadjuvant chemotherapy, adjuvant chemotherapy, or radical concurrent chemoradiotherapy);<br>5. The Eastern Cooperative Oncology Group (ECOG) score: 0~1 point (see Annex 1);<br>6. Expected survival ≥12 weeks;<br>7. Patients who have at least one measurable lesion according to Response Evaluation Criteria in Solid Tumors (RECIST 1.1), and the lesion should not have received radiotherapy or other local treatment;<br>8. The function of vital organs meet the following requirements (it is not allowed to use any blood component, cell growth factors, white blood cell-increasing drugs, platelet-increasing drugs, and drugs to correct

	anemia within 14 days prior to the first dose of the study drug);<br>a. Absolute neutrophil count (ANC) ≥ 1.5 × 10^9/L;<br>b. Platelet count ≥ 100 × 10^9/L;<br>c. Hemoglobin ≥ 9 g/dL;<br>d. Serum albumin ≥ 2.8 g/dL;<br>e. Total bilirubin ≤ 1.5 × ULN, ALT, AST and/or AKP ≤ 2.5 × ULN; if there is liver metastasis, ALT and/or AST ≤ 5 × ULN; if there is liver metastasis or bone metastasis, AKP ≤ 5 × ULN ;<br>f. Serum creatinine ≤1.5 × ULN or creatinine clearance >60 mL/min (Cockcroft-Gault, see annex II);<br>g. Activated partial thromboplastin time (APTT) and international normalized ratio (INR) ≤ 1.5 × ULN (patients on stable doses of anticoagulant therapy such as low molecular weight heparin or warfarin whose INR is within the expected therapeutic range of anticoagulants can be screened);<br>9. Female subjects with childbearing potential and male subjects whose partners are women of childbearing age should take one medically recognized contraceptive measure (IUD, birth control pills or condoms) during the study treatment, period, at least 3 months after the last dose of camrelizumab/placebo, and at least 6 months after the last use of chemotherapy;<br>10. Subjects who are voluntary to join the study, sign the informed consent form, have good compliance and cooperate with follow-up.
**Exclusion criteria**	Patients in line with any of the following conditions may not enter the study.<br>1. Locally advanced patients, who can receive radical treatment such as surgery, radical radiotherapy or radical radiochemotherapy, will not be screened;<br>2. Patients with a past history of allergy to any component of camrelizumab, gemcitabine, cisplatin and other platinum drugs;<br>3. Patients with clinically symptomatic central nervous system metastases such as cerebral edema and requiring hormone intervention, or progression of brain metastases. Patients who have previously received brain or meningeal metastasis treatment, if they are stable both as shown by MRI and clinically (do not require > 10 mg/day prednisone or equivalent dose of hormone therapy), can be included;<br>4. Patients previously or concurrently suffering from other malignant tumors (except for malignant tumors which have been cured with cancer-free survival of more than 5 years, such as cutaneous basal cell carcinoma, cervical carcinoma in situ, papillary thyroid carcinoma, etc.);<br>5. Patients with uncontrolled cardiac clinical symptoms or diseases, such as: (1) NYHA grade II or higher heart failure(2) unstable angina (3) myocardial infarction that has occurred in the past 1 year (4) supraventricular or ventricular arrhythmia with clinical significance and requiring clinical intervention;<br>6. Patients who have received any of the following treatments:

	a. Patients who have previously received anti-PD-1, anti-PD-1 antibody or anti-CTLA-4 antibody treatment;<br>b. Patients who have used any investigational drugs within 4 weeks prior to the first dose of the study drug;<br>c. Patients who are concurrently enrolled into another clinical study, unless the study is observational (non-interventional) clinical study or interventional clinical study follow-up;<br>d. Patients who received the last dose of anti-cancer treatment (including chemotherapy, radiotherapy, targeted therapy, etc.) within ≤ 4 weeks prior to the first dose of the study drug;<br>e. Subjects who need to receive systematic treatment with corticosteroids (>10 mg prednisone equivalent dose/day) or other immunosuppressive agents, except for the use of corticosteroids for local inflammation and prevention of allergies and nausea and vomiting. Other special cases require communication with the sponsor. In the absence of active autoimmune disease, inhaled or topical steroids and adrenal cortical hormone replacement therapy with at a dose >10 mg/day therapeutically effective dose of prednisone are allowed;<br>f. Patients who have received inoculation of tumor vaccines or have received live vaccines within 4 weeks prior to the first dose of the study drug;<br>g. Patients who have undergone major surgery or had a severe trauma within 4 weeks prior to the first dose of the study drug;<br>7. Patients whose toxicity due to previous anti-tumor treatment has not recovered to CTC AE ≤ grade 1 (except for hair loss, and sequelae of previous platinum treatment-related neurotoxicity) or the level prescribed by the inclusion/exclusion criteria;<br>8. Patients who have developed severe infection (CTC AE > grade 2) within 4 weeks prior to the first dose of the study drug, such as severe pneumonia, bacteremia and complication of infection that require hospitalization; patients whose baseline chest imaging findings suggest active pulmonary inflammation, or patients with symptoms and signs of infection within 2 weeks prior to the first dose of the study drug or need to be treated with oral or intravenous antibiotics (excluding prophylactic use of antibiotics);<br>9. Patients with active autoimmune diseases or a history of autoimmune diseases (e.g., interstitial pneumonia, colitis, hepatitis, pituitary inflammation, vasculitis, nephritis, hyperthyroidism and hypothyroidism, including but not limited to these diseases or syndromes); but excluding autoimmune-mediated hypothyroidism using a stable dose of thyroid replacement hormone therapy; type I diabetes using a stable dose of insulin; patients with vitiligo or cured childhood asthma/allergy that does not require any intervention after adulthood;<br>10. Patients with a history of immunodeficiency, including HIV positive, or

	other acquired or congenital immunodeficiency diseases, or a history of organ transplantation and a history of allogeneic bone marrow transplantation;<br>11. Patients with a history of interstitial lung disease (excluding radiation pneumonitis without hormone therapy),history of non-infectious pneumonia;<br>12. Patients with active tuberculosis infection found by medical history or CT examination, or patients with a history of active tuberculosis infection within 1 year prior to enrollment, or patients with a history of active tuberculosis infection more than 1 year ago who have not received standardized treatment;<br>13. Subjects who have active hepatitis B (HBV DNA ≥ 2000 IU/mL or 10^4 copies/mL), hepatitis C (positive hepatitis C antibody, and HCV-RNA is higher than the lower limit of detection of the analytical method);<br>14. Subjects known to have a history of psychotropic substance abuse, alcohol abuse or drug abuse;<br>15. Women during pregnancy or lactation;<br>16. At the discretion of the investigator, subjects who have other factors that may lead to forced termination of the study halfway, such as other serious illnesses (including mental illness) requiring concomitant treatment, severe laboratory test abnormalities, family or social factors, or conditions that may affect subject safety or test data collection.
**Immunogenicity study**	Time for collection of blood samples: C1D1, C2D1, C4D1, C6D1, C9D1, once before each administration of camrelizumab/placebo, once before administration every 4 cycles thereafter, when the subject withdraws from the study treatment, and once each at 30 days, 60 days and 90 days after the last dose (according to visit arrangement, if applicable).
**Criteria for withdrawal from the study**	The reasons for subjects' withdrawal from the study may include:<br>1. The subject withdraws the informed consent, and rejects further follow-up;<br>2. The subject is lost to follow-up;<br>3. The subject is dead;<br>4. The sponsor terminates the study.
**Criteria for termination of study treatment**	The criteria for subjects' termination of the study treatment are as follows:<br>1. The subject withdraws informed consent and refuses to continue receiving the study drug treatment;<br>2. As judged by the investigator, the subject's clinical symptoms deteriorate/performance status declines;<br>3. The subject is judged as progressive disease according to RECIST 1.1 criteria. Subjects meeting the criteria for clinically stable (see Section 0) can continue the medication until confirmed PD or the investigator judges that the subject can no longer clinically benefit;<br>4. Camrelizumab/placebo has been cumulatively used for up to 2 years (without imaging progression);<br>5. The toxicity is intolerable, including the occurrence of any clinical AE,

	laboratory test abnormalities or other medical conditions. If a subject cannot tolerate camrelizumab or chemotherapy toxicity, continued use of the drug with intolerable toxicity should be terminated;<br>6. There are other situations where the investigator thinks it necessary to terminate the study drug treatment;<br>7. The subject is pregnant;<br>8. The subject is lost to follow-up;<br>9. The subject is dead;<br>10. The sponsor terminates the study.
**Determination of sample size**	The study is a parallel controlled study. The primary study endpoint is IRC-assessed PFS. The parameters for calculation of sample size are as follows:<br>1. Enrollment duration=12 months, follow-up duration=24 months (the overall duration is 36 months)<br>2. 1:1 randomized<br>3. α=0.025 (one-sided)<br>4. Hazard ratio (HR, test group/control group)=0.63 (the median PFS in the control group is 7 months)<br>An interim analysis is planned for PFS. The Hwang-Shih-DeCani (gamma=-4) O'Brien-Fleming spending function is used to allocate α. The interim analysis of PFS will be performed when 121 (60%) events are collected. The final analysis of PFS will be performed when 201 events are collected. Under the premise of ensuring that the overall type I error does not exceed one-sided 0.025, a power of about 90% can be obtained. Given a dropout rate of 10% in the course of study, eventually 250 subjects need to be enrolled (125 cases in each group).
**Data analysis/statistical method**	For time-dependent event indicators (e.g., PFS, OS, etc.), the Kaplan-Meier method will be used to estimate the survival function in the two groups and plot the survival curves; meanwhile, the log-rank test will be used to compare between the groups. Besides, the Cox regression model will be used to estimate the HR between the two groups and its 95% confidence interval (95% CI).<br>For dichotomous variables, Cochran-Mantel-Haenszel can be used to estimate the between-group difference and its 95% confidence interval (95% CI).<br>For safety analysis, descriptive statistics will be used for summary.
**Study time**	Expected time of enrollment of the first subject: November 2018<br>Expected time of enrollment of the last subject: November 2019<br>Expected time of end of the study: 2 years after the last enrolled subject's first dose

Trial Flowchart

Study Flowchart

	**Screening period**		**Treatment period**			**Withdrawal from study treatment visit [24] (+3 days)**	**Safety follow-up [25]**		**Survival follow-up (±7 days) [26]**
			**Combination chemotherapy**		**Maintenance treatment [27]**				
**Visit window**	**D-28~D-1**	**D-7~D-1**	**D1±3 days**	**D8±3 days**	**Every 3 weeks D1±3 days**		**30 days after the last treatment (±7 days)**	**60 and 90 days after the last treatment (±7 days)**	
Signing of informed consent [1]	√								
Checks of inclusion and exclusion criteria		√							
Demographic data	√								
Medical history [2]	√								
ECOG score [3]		√	√		√	√			
Vital signs [4]		√	√		√	√			
Physical examination [5]		√	√		√	√			
Blood routine test [6]		√	√	√	√	√	√		
Urine routine test [7]		√	√		√	√			
Fecal occult blood [8]		√	√		√	√			

	**Screening period**		**Treatment period**			**Withdrawal from study treatment visit [24] (+3 days)**	**Safety follow-up [25]**		**Survival follow-up (±7 days) [26]**
			**Combination chemotherapy**		**Maintenance treatment [27]**				
**Visit window**	**D-28~D-1**	**D-7~D-1**	**D1±3 days**	**D8±3 days**	**Every 3 weeks D1±3 days**		**30 days after the last treatment (±7 days)**	**60 and 90 days after the last treatment (±7 days)**	
Blood biochemistry [9]		√	√		√	√	√		
Thyroid function [10]		√	√		√	√	√		
Coagulation function [11]		√							
EBV-DNA testing [12]	√		√		√	√			
Virological examination [13]	√								
Electrocardiogram [14]		√	√		√	√			
Echocardiography [15]		√							
Pregnancy test [16]		√				√			
Tumor imaging examination [17]	√				√				
Study randomization		√							
Camrelizumab/placebo administration [18]			√		√				
Gemcitabine administration [19]			√	√					

	Screening period		Treatment period			Withdrawal from study treatment visit [24] (+3 days)	Safety follow-up [25]		Survival follow-up (±7 days) [26]
			Combination chemotherapy		Maintenance treatment [27]				
**Visit window**	**D-28~D-1**	**D-7~D-1**	**D1±3 days**	**D8±3 days**	**Every 3 weeks D1±3 days**		**30 days after the last treatment (±7 days)**	**60 and 90 days after the last treatment (±7 days)**	
Cisplatin administration [20]			√						
AE [21]	√	√	√	√	√	√	√	√	
Concomitant medication [22]	√	√	√	√	√	√	√	√	
ADA blood sampling [23]			√		√	√	√	√	

Note: In addition to the examinations and time points listed in the table above, the investigator may increase visits and other examinations at any time as actually needed, and the examination results will be filled out in the eCRF *Unscheduled Visit*.

[1] Before the start of screening, written informed consent form signed by the subject or his/her legal agent/independent witness must be obtained first.

[2] Medical history: including tumor history (tumor diagnosis, surgery, radiotherapy, drug treatment) and other history of concorrent diseases or history of drug allergy.

[3] ECOG score: within 7 days prior to the first dose, before administrtaion on Day 1 of each cycle (if the test in the screening period is completed within 7 days prior to the first dose, it is not necessary to repeat the test before the first dose), and when the subject withdraws from the study treatment.

[4] Vital signs: blood pressure, pulse, body temperature, respiratory rate, within 7 days prior to the first dose, before administrtaion on Day 1 of each cycle (if the test in the screening period is completed within 7 days prior to the first dose, it is not necessary to repeat the test before the first dose), and when the subject withdraws from the study treatment.

[5] Physical examination: within 7 days prior to the first dose and when withdrawing from the study treatment, the subject receives comprehensive physical examination (including general conditions, head and face, neck, skin, lymph nodes, eyes, ENT, mouth, respiratory system, cardiovascular system, abdomen, reproductive-urinary system, musculoskeletal, nervous system, mental state, other); before administrtaion on Day 1 of each cycle (if the test in the screening period is completed within 7 days prior to the first dose, it is not necessary to repeat the examination before the first dose) and when clinically indicated, targeted physical examination is performed.

[6] Blood routine test: red blood cell count, hemoglobin, platelet count, white blood cell count, neutrophil count and lymphocyte count. Within 7 days prior to the

first dose, before administrtaion on Days 1 and 8 of each subseqeunt cycle (if the test in the screening period is completed within 7 days prior to the first dose, it is not necessary to repeat the test before the first dose), when the subject withdraws from the study treatment, and at 30 days after the last treatment.

[7] Urine routine test: white blood cells, red blood cells, and urine protein. Within 7 days prior to the first dose, before administrtaion on Day 1 of every 2 subsequent cycles, and when the subject withdraws from the study treatment (if the test in the screening period is completed within 7 days prior to the first dose, it is not necessary to repeat the test before the first dose). If urine protein is ≥ 2+, 24-hour urine protein quantification must be performed.

[8] Fecal occult blood: 7 days prior to the first dose, on Day 1 of every 2 subsequent cycles (either before or after administration), and when the subject withdraws from the study treatment.

[9] Blood biochemistry: ALT, AST, GGT, total bilirubin, direct bilirubin, AKP, blood urea nitrogen or urea (preferably blood urea nitrogen), total protein, albumin, creatinine, blood glucose, lactate dehydrogenase , K^+, Na^+, Ca^{2+}, Mg^{2+} and Cl^-. Within 7 days prior to the first dose, before administrtaion on Day 1 of each subsequent cycle (if the test in the screening period is completed within 7 days prior to the first dose, it is not necessary to repeat the test before the first dose), when the subject withdraws from the study treatment, and at 30 days after the last treatment.

[10] Thyroid function: TSH, FT3, FT4. Within 7 days prior to the first dose, before administrtaion on Day 1 of every 2 subsequent cycles, when the subject withdraws from the study treatment, and at 30 days after the last treatment.

[11] Coagulation function: APTT, PT, FIB, INR. Within 7 days prior to the first dose.

[12] EBV-DNA testing: at baseline screening period, before administration on Day 1 of every 3 cycles, and when the subject withdraws from the study treatment.

[13] Virological examination: HBsAg (if positive, HBV-DNA testing is required), HBsAb, HBeAg, HBeAb, HBcAb, HCV-Ab (if positive, HCV-RNA testing is required), and HIV-Ab. Within 14 days prior to the first dose.

[14] Electrocardiogram: within 7 days prior to the first dose, before administration on Day 1 of each cycle (if the test in the screening period is completed within 7 days prior to the first dose, it is not necessary to repeat the test before the first dose), and when the subject withdraws from the study treatment.

[15] Echocardiography: tested as clinically suggested within 7 days prior to the first dose.

[16] Pregnancy test: for women of childbearing age, either blood or urine pregnancy test is acceptable. Within 72 hours prior to the first dose, and when the subject withdraws from the study treatment.

[17] Tumor imaging examination: CT or MRI (both enhanced, except for cases where the contrast agent is contraindicated and a plain scan can be performed instead) of the nasopharynx, neck, chest, and abdomen (including pelvis). Brain MRI is required when the subject is suspected and diagnosed with brain metastasis (in cases where MRI is contraindicated, CT can be performed instead, both enhanced, except for cases where the contrast agent is contraindicated and a plain scan can be performed instead). Bone scan is performed only when clinically indicated.

- Screening period: the imaging examination results obtained before the signing of informed consent, as long as they meet the RECIST 1.1 requirements, can be used for baseline tumor assessment. The window for baseline imaging examination is extended to within 4 weeks prior to randomization. Bone scan is required when the subject is suspected or confirmed to have bone metastasis, and the time of examination is extended to within 42 days prior to randomization.
- Treatment period: baseline imaging examination is repeated every 6 weeks, and every 12 weeks since 16 months after the first dose. Unplanned imaging examination can be performed if progressive disease is suspected. Subjects who withdraw from the study treatment for any reason should receive imaging examination promptly (±4 weeks, if the first examination is no more than 4 weeks from the withdrawal from study treatment visit, it is not necessary to perform the examination again). The imaging examination conditions should the same as baseline (including scan thickness and contrast agent, etc.).

- The allowed window period for imaging examination is ±7 days. Except for imaging-confirmed PD, subjects whose study treatment is terminated for other reasons should also receive an imaging examination every 6 weeks, and every 12 weeks (±7 days) since 16 months after the first dose until observation of progressive disease, initiation of a new anti-tumor treatment, withdrawal of informed consent , loss to follow-up or death.

[18] Camrelizumab/placebo administration: administered on Day 1 of each cycle, every 3 weeks as 1 dosing cycle.

[19] Gemcitabine administration: during the combination therapy, administered on Days 1 and 8 of each cycle, every 3 weeks as 1 dosing cycle, for 4-6 cycles, because of no less than 3 cycles in subjects with intolerable toxicity.

[20] Cisplatin administration: during the combination therapy, administered on Day 1 of each cycle, every 3 weeks as 1 dosing cycle, for 4-6 cycles, because of no less than 3 cycles in subjects with intolerable toxicity.

[21] AE: the collection time begins with the signing of informed consent form, until Day 90 after the last administration of camrelizumab/placebo or Day 30 after the last administration of gemcitabine or cisplatin (whichever occurs later). If the patient starts a new anti-tumor treatment during the AE collection period, only AEs related to the study drug are collected after the new anti-tumor treatment.

[22] Concomitant medication: record all concomitant medications from within 30 days before the signing of informed consent through to the start of a new anti-tumor tretament. Thereafter, only record concomitant medications for AEs related to the study drug.

[23] ADA blood sampling: C1D1, C2D1, C4D1, C6D1, C9D1, once before each administration of camrelizumab/placebo, once before administration every 4 cycles thereafter, when the subject withdraws from the study treatment, and once each at 30 days, 60 days and 90 days after the last dose (according to visit arrangement, if applicable).

[24] Withdrawal from study treatment visit: this is completed when it is confirmed that the subject needs to terminate the study treatment (the assessment and examination performed within 7 days prior to confirming that the subject needs to terminate the study treatment do not need to be repeated).

[25] Safety follow-up period: all subjects need to visit the study site at 30 days after the last dose (if the withdrawal from study treatment visit is completed, this visit is changed to telephone follow-up), and telephone follow-up is required at 60 and 90 days after the last dose. Telephone follow-up needs to obtain safety information (including AE outcomes, new SAEs, AEs of special interest, concomitant medications, etc.), with a window period of ±7 days.

[26] Survival follow-up: once every 3 months after the last dose, with a window period of ± 7 days.

[27] Maintenance treatment: camrelizumab/placebo maintenance treatment, administered on Day 1 of each cycle, every 3 weeks as 1 dosing cycle.

Abbreviations

Abbreviation	English full name	Chinese full name
ADA	Anti-drug antibody	抗药抗体
ADL	Activities of daily living	日常生活活动
AE	Adverse event	不良事件
AKP	Alkaline phosphatase	碱性磷酸酶
ALT	Alanine aminotransferase	谷氨酸丙氨酸氨基转移酶
ANC	Absolute neutrophil count	中性粒细胞计数
APTT	Activated partial thromboplastin time	活化部分凝血活酶时间
AST	Aspartate aminotransferase	谷氨酸天门冬氨酸氨基转移酶
AUC	Area under the curve	药时曲线下面积
BUN	Blood urea nitrogen	尿素氮
CNDA	China National Drug Administration	国家药品监督管理局
CR	Complete remission	完全缓解
Cr	Creatinine	肌酐
CRF	Case report form	病例报告表
CRO	Contract research organization	合同研究组织
D	Day	天
DoR	Duration of response	缓解持续时间
DLT	Dose-limiting toxicity	剂量限制毒性
EC	Ethics committee	伦理委员会
EDC	Electronic data collection	电子数据收集
FAS	Full Analysis Set	全分析集
FIB	Plasma fibrinogen	纤维蛋白原
FT3	Free triiodothyronine	游离三碘甲状腺原氨酸
FT4	Free thyroxine	游离甲状腺素
GC	Gastric carcinoma	胃癌
GCP	Good Clinical Practice	药物临床试验质量管理规范
GGT	Gamma Glutamyl Transpeptidase	谷氨酰转肽酶
h	Hour	小时
HCC	Hepatocellular Carcinoma	肝癌
Hb	Hemoglobin	血红蛋白
HR	Hazard ratio	风险比
IC_{50}	Half maximal inhibitory concentration	50%抑制浓度
iRECIST	Immune-related response evaluation criteria in solid tumors	免疫相关实体瘤的疗效评价标准
IRC	Independent Review Committee	独立（影像）评估委员会
IU	International unit	国际单位
LDH	Lactate dehydrogenase	乳酸脱氢酶
MMF	mycophenolate mofetil	吗替麦考酚酯
MRI	Magnetic resonance imaging	核磁共振
MTD	Maximal Tolerable Dose	最大耐受剂量
NOAEL	No Observed Adverse Effect Levl	无明显不良作用水平
NPC	Nasopharyngeal Cancer	鼻咽癌
NSCLC	Non-Small Cell Lung Cancer	非小细胞肺癌
ORR	Objective response rate	客观缓解率

Abbreviation	English full name	Chinese full name
OS	Overall survival	总生存期
PR	Partial remission	部分缓解
PD	Progressive disease	疾病进展
PK	Pharmacokinetic	药代动力学
PFS	Progression free survival	无进展生存期
PPS	Per-protocol Analysis Set	符合方案集
PT	ProthrombinTime	凝血酶原时间
PLT	Blood platelet	血小板
RBC	Red blood cell count	红细胞计数
RECIST	Response evaluation criteria in solid tumors	实体瘤疗效评价标准
SAE	Serious adverse event	严重不良事件
SAP	Statistical analysis plan	统计分析计划
SD	stable disease	疾病稳定
SS	Safety Analysis Set	安全集
T-BIL	Total bilirubin	总胆红素
TNBC	Triple Negative Breast Cancer	三阴乳腺癌
TSH	Thyroid-stimulating hormone	促甲状腺素
UA	Uric acid	尿酸
ULN	Upper limit of normal	正常值上限
WBC	White blood cell count	白细胞计数

1 Introduction: Study Background and Scientific Rationale

1.1 Study Background

1.1.1 Nasopharyngeal Carcinoma

Nasopharyngeal carcinoma is a relatively rare tumor in the world, but it is one of the high-incidence tumors in the Asian population. The age-standardized rate (ASR) is 2.3 for men and 0.9 for women. Especially in the Southeast Asian population, the ASR is 6.4 men and 2.4 for women [1]. Nasopharyngeal carcinoma in China also shows a regionally high incidence. It has the highest incidence among head and neck tumors, mainly concentrated in southern China, with an annual incidence of 80/100,000 [2].

Radiotherapy is the main method for the treatment of early nasopharyngeal carcinoma. The 5-year overall survival rates of patients with stage I and IIA are 90% and 84%, respectively, but about 20-37% of patients will have local recurrence or distant metastasis [3]. With very limited treatment means available, relapsed or metastatic nasopharyngeal carcinoma has a median survival of mostly about 20 months [4]. Platinum-containing dual-agent chemotherapy is a commonly used first-line treatment for relapsed or metastatic nasopharyngeal carcinoma. Professor Zhang Li from Sun Yat-sen University Cancer Center compared the efficacy and safety of cisplatin combined with gemcitabine (GP) and cisplatin combined with 5-FU (FP) for the first-line treatment of relapsed or metastatic nasopharyngeal carcinoma in a randomized controlled multicenter phase III clinical trial [5]. The study enrolled 362 patients. The results showed that the median progression-free survival (PFS) of GP and FP was 7.0 months and 5.6 months, respectively, and the objective response rate (ORR) was 64% and 42%, respectively. The overall survival (OS) was 29.1 months and 20.1 months, respectively. Grade 3/4 AE whose incidences were significantly different between GP and GF were leukopenia (29% vs 15%), neutropenia (23% vs 13%), thrombocytopenia (13% vs 2%) and mucositis (0 vs 14%). It can be seen from the results that the GP regimen improved efficacy compared with GF and significantly reduced the incidence of mucositis, but blood system abnormalities were its common grade 3/4 adverse events. Although the GP regimen prolonged the patients' survival compared to GF, its therapeutic effect was still not optimal, and new therapeutic drugs/scenarios are urgently needed in clinical practice to enhance the efficacy and safety of treatment of relapsed or metastatic nasopharyngeal carcinoma.

1.1.2 Camrelizumab

Camrelizumab (compound development No. SHR-1210) is a humanized monoclonal antibody developed by Hengrui Medicine Co., Ltd., in which the heavy chain is immunoglobulin G4 (IgG4) and the light chain is immunoglobulin κ (IgK), expressed in the supernatant of Chinese hamster ovary (CHO) stable cell line. Camrelizumab binds specifically to PD-1 and blocks the interaction of PD-1 with its ligand (PD-L1), allowing T cells to restore immune responses against tumors.

1.1.2.1 Preclinical Study Results

In vitro pharmacodynamic studies showed that camrelizumab had a high affinity for human, cynomolgus monkey and rhesus monkey PD-1 (2 nM, 8 nM, 4 nM, respectively), and could effectively block PD-1/PD-L1 interaction (IC_{50} was 0.70 nM). In vivo pharmacodynamic studies showed that camrelizumab had a significant antitumor effect in the mouse MC38 colorectal cancer model and the human malignant glioblastoma U87-MG mouse xenograft model.

Clinical pharmacokinetic studies in cynomolgus monkeys showed an increase in exposure (AUC and C_{max}) with increasing doses, no gender differences in PK parameters, dose-dependent clearance rates, and lower clearance rates at higher doses. There was no drug accumulation after repeated administration.

Toxicological studies showed that the maximum tolerated dose (MTD) was ≥ 800 mg/kg for single-dose administration of camrelizumab in cynomolgus monkeys, and ≥200 mg/kg for repeated-dose administration. In the 4-week and 26-week toxicity studies, the No Observed Adverse Effect Level (NOAEL) dose of carelibizumab was both 100 mg/kg.

Preclinical pharmacological studies have demonstrated that camrelizumab has good antitumor activity, safety and tolerability, and support further clinical research on camrelizumab. See the Investigator's Brochure for detailed preclinical pharmacological data.

1.1.2.2 Clinical Study Results

As of February 28, 2018, 6 Phase I clinical studies, 9 Phase II clinical studies, and 2 Phase III clinical studies had been conducted on camrelizumab.

SHR-1210-I-101 was an open, single-center, dose-escalation Phase I clinical study to assess the safety and tolerability of careliuzumab in patients with advanced solid tumors after standard antitumor therapy failure. The study consisted of 3 stages. Stage 1 was the dose escalation part. In the standard 3 + 3 dose escalation design, camrelizumab was intravenously infused at a dose of 1 mg/kg, 3 mg/kg (roughly equivalent to a fixed dose of 200 mg) and 10 mg/kg, Q2W (except for the first 4-week cycle, in which subjects only received the study drug on Day 1 for PK sampling and dose-limiting toxicity [DLT] observation); Stage 2 was the extension stage, and the study was extended in the doses selected at Stage 1, with up to 12 subjects in each group; Stage 3 was clinical extension using a fixed-dose group (200 mg/time), where subjects with nasopharyngeal carcinoma and lung cancer who may be accompanied by brain metastases were enrolled. As of February 28, 2018, a total of 123 subjects were enrolled in the study.

SHR-1210-I-102 was an open, single-center, dose-escalation Phase I study to assess the safety and tolerability of careliuzumab in patients with advanced malignant melanoma after standard antitumor therapy failure. The study consisted of 2 stages. Stage 1 was the dose escalation part. In the standard 3 + 3 dose escalation design, camrelizumab was intravenously infused at a fixed dose of 60 mg, 200 mg and 400 mg, Q2W (except for the first 4-week cycle, in which subjects only received the study drug on Day 1 for PK sampling and DLT observation); Stage 2 was the extension stage, and the study was extended in the doses selected at Stage 1, with up to 12 subjects in each group. As of February 28, 2018, the study had completed the enrollment of all 36 subjects.

SHR-1210-I-103 was an open, single-center, dose-escalation Phase I study to assess the safety and tolerability of careliuzumab in patients with advanced solid tumors after standard antitumor therapy failure. The study consisted of 3 stages. Stage 1 was the dose escalation part. In the standard 3 + 3 dose escalation design, camrelizumab was intravenously infused at a fixed dose of 60 mg, 200 mg and 400 mg, Q2W (except for the first 4-week cycle, in which subjects only received the study drug on Day 1 for PK sampling and DLT observation); Stage 2 was the extension stage, and the study was extended in the doses selected at Stage 1, with up to 9-12 subjects in each group. Stage 3 would further explore the Q2W dosage of administration and be conducted in subjects with EC, GC, HCC and TNBC (60 subjects in total). As of February 28, 2018, the study had completed the enrollment of all 99 subjects.

SHR-1210-104 was an open, single-center, one-arm, Phase I study to evaluate the safety of camrelizumab in combination with gemcitabine and cisplatin in the first-line treatment of patients with relapsed or metastatic nasopharyngeal carcinoma. Subjects received camrelizumab (200 mg/time, on D1 of each cycle) in combination with gemcitabine (1000 mg/m^2, on D1 and D8 of each cycle) and cisplatin (80 mg/m^2, on D1 of each cycle) with every 3 weeks as 1 treatment cycle, and the combination medication lasted for up to 6 cycles. Thereafter camrelizumab could be used alone for maintenance treatment until the disease progressed, the toxicity was intolerable, the subject withdrew his/her informed consent, or the investigator determined that the subject was unsuitable for continued treatment. As of February 28, 2018, the study had completed the enrollment of all 23 subjects.

1.1.2.2.1 Safety Results

Three Phase I clinical studies, SHR-1210-I-101/102/103, enrolled a total of 258 subjects, and no DLT was observed. 258 (100.0%) subjects had at least one AE, 98 (38.0%) subjects had at least one grade 3 or higher AE, and 256 (99.2%) subjects had at least one drug-related AE, 82 (31.8%) subjects had at least one grade 3 or higher drug-related AE, and 78 subjects experienced SAE which was drug-related in 61 cases. SHR-1210-related AEs were mostly CTCAE grade1-2. Common drug-related AEs were mainly skin and subcutaneous tissue diseases such as skin capillary hyperplasia (81.8%), itching (22.1%) and rash (16.3%); systemic diseases such as fatigue (37.6%) and fever (20.9%); blood and lymphatic system diseases such as anemia (27.5%); various tests: elevated aspartate aminotransferase (21.7%), elevated alanine aminotransferase (18.6%), elevated bilirubin (16.7%), decreased white blood cell count (14.7%), decreased blood sodium (14.3%), and elevated blood bilirubin (12.0%); kidney and urinary system diseases such as proteinuria (22.1%); endocrine system diseases such as hypothyroidism (19.8%); metabolic and nutritional diseases such as hypoproteinemia (19.4%); respiratory, chest and mediastinal diseases such as cough (19.0%); gastrointestinal disorders such as diarrhea (11.2%), and nausea (10.5%); infection and infectious diseases such as upper respiratory tract infection (10.1%). Except for skin capillary hyperplasia, other AEs were similar to those of similar drugs.

A total of 23 subjects were enrolled in the SHR-1210-I-104 study. All the 23 (100%) subjects had at least one AE, and 21 subjects (91.3%) had at least one grade 3 or higher AE. All the 23 (100%) subjects had at least one drug-related AE, and 22 (95.7%) subjects had at least one immune-related AE (irAE). 2 subjects(8.7%) experienced SAE, but all the SAEs were unrelated to the study drug. The AEs related to camrelizumab were mostly CTCAE grade 1-2. Common drug-related AEs were mainly various tests, such as decreased free thyroxine (56.5%), elevated blood thyroid hormone (52.2%), decreased free triiodothyronine (30.4%), elevated alanine aminotransferase (26.1%), elevated aspartate aminotransferase (26.1%), anti-thyroid antibody positive (21.7%), elevated blood triglycerides (21.7%), elevated thyroglobulin (13.0%), decreased blood thyroid hormone (13.0%), decreased blood triglyceride (8.7%); skin and subcutaneous tissue diseases such as rash (60.9%), pruritus (43.5%), skin capillary hyperplasia (26.1%), and rash itching (8.7%), systemic rash (8.7%); kidney and urinary system diseases such as proteinuria (69.6%), nervous system diseases such as hypaesthesia (60.9%); and systemic diseases and various reactions at the administration sites such as fatigue (8.7%).

Overall, camrelizumab has good safety and tolerability in patients with advanced solid tumors.

1.1.2.2.2 Effectiveness Results

In a Phase I clinical study of the safety and tolerability of camrelizumab in patients with advanced solid tumors (SHR-1210-I-101), camrelizumab demonstrated preliminary clinical effectiveness in the treatment of patients with relapsed or metastatic nasopharyngeal carcinoma after platinum-containing regimen treatment failure. A total of 92 patients with nasopharyngeal carcinoma were enrolled in the study and received treatment with camrelizumab at least once. Among them, 74 patients had completed ≥3 months of follow-up, including 9 cases in the 1 mg/kg dose group, 4 cases in the 3 mg/kg dose group, 49 cases in the 200 mg dose group and 12 cases in the 10 mg/kg dose group. The results of the efficacy evaluation using the data of the 74 patients as the total treatment analysis set were as follows: the number of patients evaluated by the investigator as CR, PR, stable disease (SD), and PD was 1, 23, 18, and 27, respectively; the overall ORR was 32.4% and the disease control rate (DCR) was 56.8%.

In the SHR-1210-I-104 study, a total of 23 subjects with primary metastatic nasopharyngeal carcinoma who had not received first-line chemotherapy or subjects with relapsed nasopharyngeal carcinoma who were unsuitable for local treatment were enrolled, and 22 of them were evaluated for efficacy. The results showed that the ORR was 91% (1 case of CR, 10 cases of PR) and the DCR was 100%. As of May 20, 2018, the median DoR had not yet been reached, 15 patients were still taking medication, and 9 cases had been treated for more than 300 days and 13 cases for over 280 days. Camrelizumab also demonstrated preliminary efficacy in subjects with primary metastatic nasopharyngeal carcinoma who had not received first-line chemotherapy or subjects with relapsed nasopharyngeal carcinoma who were unsuitable for local treatment.

1.1.2.2.3 Pharmacokinetics and Immunogenicity

Based on the pharmacokinetic statistics of 49 subjects in the first two stages of SHR-1210-101 study, after a single-dose intravenous infusion of camrelizumab in subjects with advanced solid tumors, the median time to peak (T_{max}) in different dose groups (1 mg/kg, 3 mg/kg, 200 mg/time and 10 mg/kg) was in the range of 0.58-2.50 h; the exposure in vivo and the elimination half-life ($t_{1/2}$) increased with the increase of the dose, the clearance rate (CLs) decreased slowly with the increase of the dose, and the volume of distribution (Vd) did not change substantially with the increase of the dose. After multiple doses, the serum concentration of camrelizumab in each dose group basically reached a steady state after 3-5 dosing cycles, and there was almost no steady-state accumulation. In the multiple administration stage, the overall receptor occupancy rate of camrelizumab in each dose group was maintained at about 75%, and PD-1 receptor occupancy was the theoretical premise of the anti-tumor effect of camrelizumab. This result suggests that camrelizumab administered at the dosing frequency of Q2W can fully occupy the PD-1 receptor and block the PD-1/PD-L1 signaling pathway.

A total of 117 subjects in the above-mentioned three Phase I clinical studies (SHR-1210-I-101/102/103) had immunogenic results, and 20 (17.1%) subjects had been positive for anti-camrelizumab antibody at least once. Among them, 3 (2.6%) subjects were positive for anti-camrelizumab antibody at baseline; 17 (14.5%) subjects were negative at baseline and positive after baseline, of whom 10 (8.5%) subjects were transiently positive. Among the abovementioned anti-camrelizumab antibody-positive subjects, neutralizing antibody activity was detected in only 1 (0.9%) subject (the subject was negative for anti-camrelizumab antibody at baseline, and continuously positive after baseline), and the subject was positive for neutralizing activity only once before Cycle 2 administration and negative in the following cycles. Being anti-camrelizumab antibody positive has not been observed to have a significant effect on the safety, efficacy or drug clearance rate of the subjects.

1.2 Scientific Basis

1.2.1 Basis of Study

Chemotherapeutic drugs used to be thought to directly kill tumor cells mainly through cytotoxicity. With the deepening of the research, it has been found that chemotherapeutic drugs can also exert anti-tumor effects by regulating the body's immune system, cause immunogenic death of tumor cells, increase the antigen cross-presenting ability of dendritic cells, and activate the body's anti-tumor immune effect [1]; reduce myeloid-derived suppressor cells (MDSC), and resolve the immunosuppressive effect induced by it [6]; increase the proportion of cytotoxic lymphocytes and regulatory T cells, and reduce the inhibitory effect of regulatory T cells on immunity [7]; and block STAT6 pathway, down-regulate PD-L2 expressed by dendritic cells and tumor cells, increase T cell activity and tumor cell recognizability [8]. The high expression of PD-L1 is associated with tumor invasion and chemoresistance [9]. Immunological checkpoint inhibitors acting on the PD-1/PD-L1 pathway enhance tumor immune surveillance and anti-tumor immune responses by inhibiting PD-1 signaling. Therefore, the combination of immunological checkpoint inhibitors with chemotherapy can synergistically exert an anti-tumor effect in theory.

At present, a number of multinational pharmaceutical companies are developing monoclonal antibodies against PD-1, which can maximize the patient's own immune system response by blocking the binding between PD-L1 and PD-1, thereby achieving the purpose of killing tumor cells. PD-1 monoclonal antibodies Nivolumab (BMS) and Pembrolizumab (Merck) are currently the most cutting-edge PD-1 antibody drugs, which have been approved by the FDA as monotherapies for malignant melanoma, non-small cell lung cancer, and head and neck cancer.

KEYNOTE-028 was a Phase Ib clinical trial of Pembrolizumab conducted in PD-L1+ patients with advanced solid tumors. In this study, among the 27 evaluable patients with nasopharyngeal carcinoma, there were 7 cases of PR and 14 cases of SD, and the best ORR was 25.9% [10]. PD-1/PD-L1 immunological checkpoint inhibitors currently in Phase II clinical trials for the treatment of relapsed or metastatic nasopharyngeal carcinoma after platinum-containing therapy failure include Nivolumab (PD-1 inhibitor) and PDR001 (PD-1) inhibitor) of Novartis, Pembrolizumab (MK-3475) of Merck (PD-1 inhibitor) and Avelumab (anti-PD-L1 antibody) of Merck/Pfizer. The preliminary efficacy demonstrated by these clinical studies further suggests that PD-1/PD-L1 may be a new generation of drugs for the treatment of nasopharyngeal carcinoma. PD-1 monoclonal antibody combined with chemotherapy has also been shown to be effective in some solid tumors. For example, a Phase 2 clinical study (Keynote021) found that Pembrolizumab combined with first-line chemotherapy for the treatment of advanced non-small cell lung cancer (NSCLC) had significantly improved patient response rate (55% vs 29%) and progression-free survival (13 months vs 8.9 months), but no significant differences were found in treatment interruption or treatment-related deaths [11]. The study of PD-1 monoclonal antibody combined with chemotherapy in bladder cancer and breast cancer is also underway, but no clinical study has not been carried out on PD-1 monoclonal antibody combined with chemotherapy in nasopharyngeal carcinoma. Camrelizumab is a PD-1 monoclonal antibody developed by Hengrui. Apart from clinical studies on camrelizumab as monotherapy in a variety of solid tumors, its combination with chemotherapy is also a direction for exploration.

1.2.2 Rationale for Design of the Dosing Regimen

The rationale for design of the dosing regimen in this study is described as follows.

No DLR was observed in the range of calculated doses (1~10 mg/kg) and fixed doses (200 mg) of camrelizumab, and the type and frequency of AE were similar. According to the study results of SHR-1210-I-101, the objective response rate was higher in the 200 mg dose group (33%), and the pharmacokinetic behavior was basically the same as that in the 3 mg/kg dose group. The PD-1 receptor occupancy rate at 22 days after administration remained at around 75%. Based on the preliminary data of pharmacokinetics, efficacy, safety and efficacy, considering the convenience of clinical operation, this study uses a fixed dose of 200 mg of camrelizumab, with every 3 weeks as 1 dosing cycle.

Platinum-containing dual-agent chemotherapy is a commonly used first-line treatment for relapsed or metastatic nasopharyngeal carcinoma [5], but there is currently no large-scale study to clarify the fixed dosing regimen of gemcitabine and cisplatin. In the aforementioned study by Professor Zhang [5], the dosing regimen of gemcitabine combined with cisplatin was gemcitabine 1 g/m^2 (D1, D8) and cisplatin 80 mg/m^2 (D1), with every 3 weeks as 1 dosing cycle for up to 6 cycles. In another study comparing a variety of chemotherapy regimens [12], the combination therapy regimen of these two drugs was gemcitabine 1250 mg/m^2 (D1, D8) and cisplatin 80 mg/m^2 (D1), with every 3 weeks as 1 dosing cycle. The ORR in this study was 91.2%. In another retrospective study [13], the combination therapy regimen of these two drugs was gemcitabine 1000 mg/m^2 (D1, D8) and cisplatin 20 mg/m^2 (D1 to D5), with every 4 weeks as 1 dosing cycle for 4-6 cycles. The PFS in this study was 15 months and the 3-year OS was 23 months. By referring to the above information, this study selects the doses of 1000 mg/m^2 (D1, D8) and 80 mg/m^2 (D1) for gemcitabine and cisplatin, respectively, with every 3 weeks as 1 dosing cycle for up to 6 cycles.

1.3 Potential Risks and Benefits

1.3.1 Known Potential Risks

Camrelizumab is an immunological checkpoint inhibitor, and subjects after receiving the drug will have temporary tumor outbreak, that is, false progression. In this study, subjects in the test group with tumor lesions that have met the RECIST1.1 criteria for progressive disease proven by imaging examination who are still clinically stable are allowed to continue receiving camrelizumab in combination with chemotherapy or camrelizumab monotherapy. Since imaging examination can hardly distinguish between false progression and true progression, this operation may cause subjects with true progression to fail to receive other anti-tumor treatments in a timely manner. Therefore, the investigator should fully inform the subject of the risk and consider the subject's imaging examination, biopsy results and clinical symptoms to determine whether the subject should continue to take the drug or not.

Drugs or therapies at any stage of the study may cause unforeseen or even severe side effects. Camrelizumab as an immunological checkpoint inhibitor may cause irAE. Safety data from previous clinical studies suggest that the incidence of irAE caused by camrelizumab is similar to that of similar drugs, and the irAEs are mainly immune-related thyroid dysfunction, pneumonia, hepatitis, and nephritis. In addition, the incidence of skin capillary hyperplasia will be high. Skin capillary hyperplasia is a benign skin reaction, and the skin is at risk of rupture and bleeding if the skin capillary hyperplasia occurs at areas exposed to frequent friction. Skin capillary hyperplasia that occurs at exposed parts such as the face will also affect the appearance of the subject to a certain extent. Other drug-related AEs are detailed in the Investigator's Brochure. Common drug-related AEs of gemcitabine include myelosuppression, nausea, vomiting and liver dysfunction, and common drug-related AEs of cisplatin include nephrotoxicity, ototoxicity, neurotoxicity, myelosuppression, nausea and vomiting. Other drug-related AEs of gemcitabine and cisplatin can be found in their package inserts for marketing. There is a low possibility of toxicity superposition when camrelizumab is combined with gemcitabine and cisplatin, but the probability of allergic reactions may increase. There are safety risks during the application of the study drug for treatment. Subjects need to be closely tracked throughout the clinical study process, and given timely interventions.

1.3.2 Known Possible Benefits

Gemcitabine combined with cisplatin is a first-line chemotherapy regimen commonly used in clinical practice for the treatment of advanced nasopharyngeal carcinoma. The combination of camrelizumab and chemotherapy may produce a synergistic anti-tumor effect, which may bring more clinical benefits for patients with advanced nasopharyngeal carcinoma.

2 Study Objectives and Study Endpoints

2.1 Study Objectives

2.1.1 Primary Study Objective

- To compare the progression-free survival (PFS) (evaluated by the Independent Review Committee [IRC]) of camrelizumab combined with cisplatin and gemcitabine versus placebo combined with cisplatin and gemcitabine in the treatment of locally advanced relapsed or metastatic nasopharyngeal carcinoma

2.1.2 Secondary study objectives

- To compare the PFS (evaluated by the investigator), overall survival (OS), objective response rate (ORR) and disease control rate (DCR) of camrelizumab combined with cisplatin and gemcitabine versus placebo combined with cisplatin and gemcitabine in the treatment of locally advanced relapsed or metastatic nasopharyngeal carcinoma, and evaluate the duration of response (DoR) in the two groups

- To evaluate the safety of camrelizumab combined with cisplatin and gemcitabine versus placebo combined with cisplatin and gemcitabine in the treatment of locally advanced relapsed or metastatic nasopharyngeal carcinoma

2.1.3 Exploratory study objective

To test the proportion of occurrence of camrelizumab antibodies

2.2 Study Endpoints

2.2.1 Primary study endpoint

- IRC-assessed PFS;

2.2.2 Secondary study endpoints

- Investigator-assessed PFS;
- ORR;
- DCR;
- DoR;
- OS;
- Safety: adverse event (AE), laboratory tests, vital signs, ECG, physical examination, etc.

2.2.3 Exploratory study endpoint

To test of the proportion of occurrence of camrelizumab antibodies

3 Study Design

3.1 Overall Design

The study uses a randomized, double-blind, placebo-controlled, multi-center study design, and chooses patients with unresectable locally advanced/relapsed or distantly metastatic nasopharyngeal carcinoma who have not previously received systemic anti-tumor treatment. The study plans to enroll 250 cases. Subjects meeting the inclusion criteria will be 1:1 randomized into test group or control group. Stratification factors include liver metastasis (yes vs. no), whether the subject has received radical concurrent radiochemotherapy (yes vs. no) and ECOG score (0 vs. 1).

Table 1 Treatment Grouping of Subjects

Treatment group	Combination therapy (3 weeks as 1 dosing cycle, for 4-6 cycles)	Maintenance treatment (3 weeks as 1 dosing cycle)
Test group	Camrelizumab + gemcitabine + cisplatin	Camrelizumab
Control group	Placebo + gemcitabine + cisplatin	Placebo

The test group receives camrelizumab (200 mg, D1), cisplatin (80 mg/m^2, D1) and gemcitabine (1000 mg/m^2, D1, D8). With 3 weeks as 1 dosing cycle, the combination therapy is given for 4-6 cycles, because of no less than 3 cycles in subjects with intolerable toxicity.

The control group receives placebo (D1), cisplatin (80 mg/m^2, D1) and gemcitabine (1000 mg/m^2, D1, D8). With 3 weeks as 1 dosing cycle, the combination therapy is given for 4-6 cycles, because of no less than 3 cycles in subjects with intolerable toxicity.

Thereafter camrelizumab/placebo is given for maintenance monotherapy until progressive disease (PD), intolerable toxicity, initiation of a new anti-tumor treatment, withdrawal of informed consent or the investigator judges that the subject needs to withdraw from the study. The longest medication duration of camrelizumab/placebo is 2 years.

Subjects can be unblinded after PD is confirmed by the IRC, except for the following three types of subjects who need to remain blind:

1. Subjects who are receiving study drug treatment and have not had IRC-confirmed disease progression;

2. Subjects who have stopped treatment and are still undergoing tumor progression follow-up, and have not had IRC-confirmed disease progression;;

3. Subjects who are lost to follow-up.

Because subjects may have pseudo-progression after receiving immune drug treatment, when a subject is first evaluated as PD according to the RECIST 1.1 criteria, and the investigator judges that the subject is clinically stable and can continue to have clinical benefits, after discussion with the sponsor, with consent, the subject may continue to use camrelizumab/placebo combined with chemotherapy or carellizumab/placebo monotherapy and receive imaging examination again after at least 4 weeks. If subsequent imaging examination confirms the subject as PD, the subject is required to discontinue camrelizumab/placebo combined with chemotherapy or camrelizumab/placebo monotherapy unless the investigator believes that the subject can continue to have clinical benefits. At this point, the investigator should discuss with the sponsor again, with the consent of the sponsor, and after the subject signs the informed consent form for continuing treatment after PD, the subject may continue the medication until the investigator determines that the subject cannot continue to have clinical benefits.

The screening period of the study is 28 days. Subjects who complete the screening examinations and assessments and meet the inclusion/exclusion criteria enter the treatment period to receive treatment according to the dosing frequency specified by the protocol, and complete relevant examinations and assessments before each administration. Imaging tumor evaluation is performed every 6 weeks (±7 days). Then imaging examination is performed every 12 weeks (±7 days) since 16 months after the first dose. All subjects complete safety examinations and imaging assessments when withdrawing from the treatment, and then enter the safety follow-up period for a total of 90 days. After withdrawing from the treatment, subjects start survival follow-up once every 3 months since the last dose. Subjects removed for non-PD reasons should receive tumor progression follow-up, and imaging assessment should be continued at the frequency of every 6 weeks (±7 days), and every 12 weeks (±7 days) since 16 months after the first dose until PD, initiation of a new anti-tumor treatment, withdrawal of informed consent, loss to follow-up or death.

The study design is shown in Figure 1.

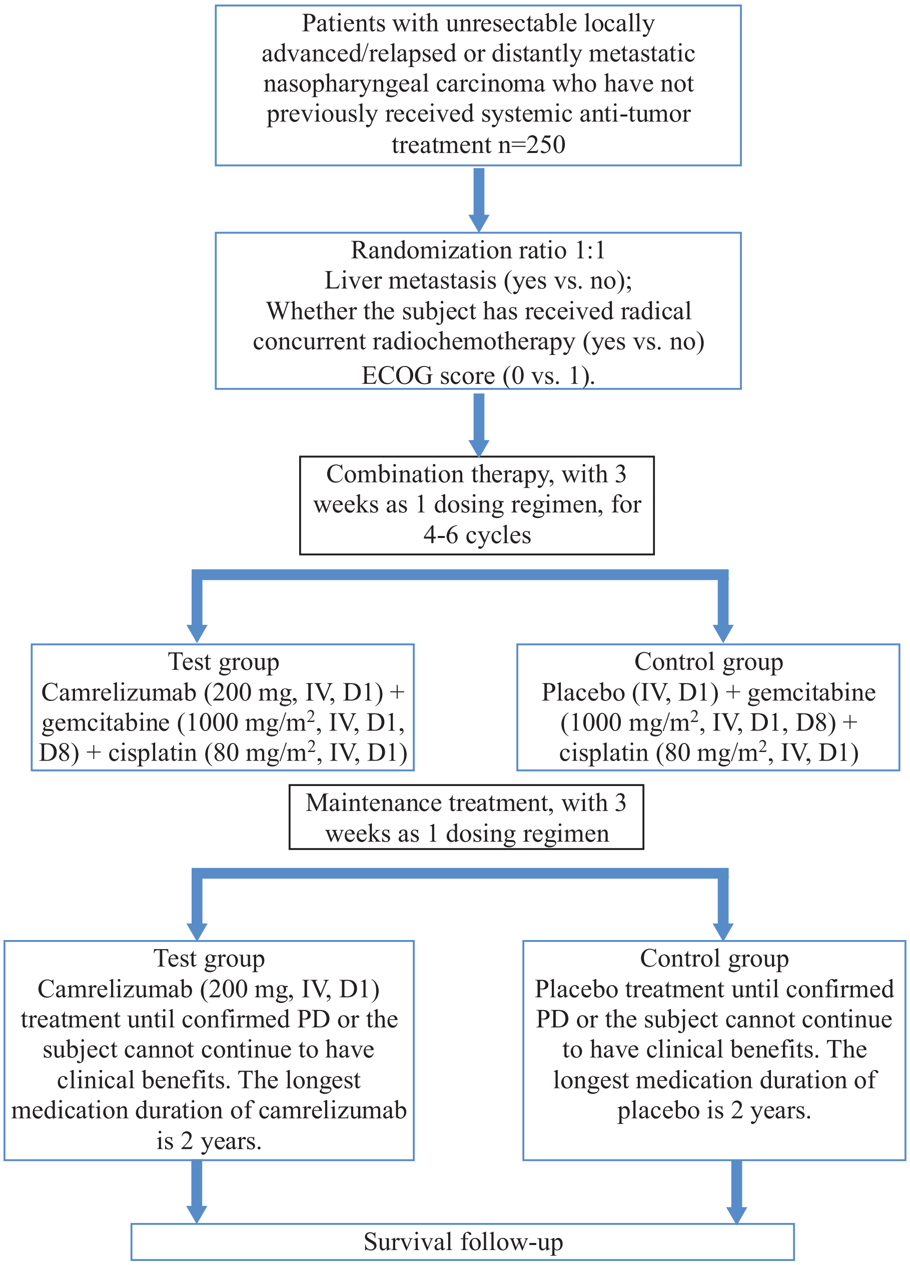

Figure 1 Study Design Diagram

3.2 Methods to Reduce Bias

3.2.1 Enrollment/Randomization/Blinding Steps

This study is a double-blind, placebo-controlled design. For subjects who have signed the informed consent form and meet the inclusion criteria, the investigator will log on the randomization system, input the subject's basic information and choose stratification factors for randomization thereof, and obtain the subject's randomization number and the assigned treatment group. The randomization ratio for the test group and the control group is 1:1. Block stratification and randomization can ensure the balance of randomization.

Camrelizumab and placebo will use the same package, and a seal label will be affixed to the opening of the packaging box to maintain blindness during drug transportation and handover processes. The double-blind technique will be used, and designated study nurses will dispense camrelizumab and placebo. Besides, during the course of the trial, dispensing nurses will not participate in the subject's administration operation as far as possible, and other study nurses will be responsible for administration. The subject, the investigator and the sponsor's staff participating in subject treatment or clinical evaluation or their designees are unaware of the grouping information.

3.2.2 Blind Evaluation

In order to reduce deviations, the imaging evaluation frequency for the test group and the control group is consistent. The imaging evaluation of tumor lesions is based on the RECIST 1.1 criteria. The primary efficacy endpoint PFS is uniformly evaluated by the Independent Imaging Review Committee (IRC) in a blind state. The final analysis strategy is determined prior to the primary effectiveness endpoint analysis database locking, including the definition of data censorship rules in advance. The effectiveness analysis will only be carried out at the time point specified in the protocol.

3.2.3 Unblinding

Identification information for treatment can only be unblinded when it is considered in the interest of the subject and deemed necessary. Subjects should not be unblinded as much as possible unless necessary.

When the investigator or his authorized person needs to confirm the drug and dosage of administration used by the subject in an emergency, the responsible investigator at the site will propose an application, and the medical director of the sponsor and the principal investigator will decide jointly whether or not to unblind. The investigator or his authorized person will use the IWRS system to unblind subjects and report the unblinding to the sponsor. Before unblinding, the investigator or his authorized person must enter the grade of toxicity of the observed adverse events, the relevance to the study drug, and the cause in the medical records and other documents.

Subjects whose treatment assignment is unblinded by the investigator/his authorized person and/or non-study doctors must stop using the study drug, but should continue to be monitored in the trial.

In addition, the investigator must log on the IWRS system and perform unblinding in the IWRS systemOnce unblinding occurs, the situation at the time of unblinding (e.g., date, reason and the person responsible for unblinding) must be recorded immediately, and the sponsor's CRA should

be notified as soon as possible. Once emergency unblinding occurs, the principal investigator, the study site's staff, and the sponsor's staff are not required to remain blind so as to provide appropriate follow-up care to the subject.

4 Subject Selection and Withdrawal

4.1 Inclusion Criteria

Subjects must meet all of the following inclusion criteria to be eligible for the study.

1. Aged ≥ 18 years and ≤ 75 years, male or female;
2. Patients pathologically diagnosed with nasopharyngeal carcinoma;
3. Patients newly diagnosed with advanced nasopharyngeal carcinoma at Stage IVb defined by the American Journal of Critical Care (AJCC) staging system (the 8th edition), or relapsed nasopharyngeal carcinoma not suitable for local treatment. Local treatment mainly refers to anti-tumor treatment-related measures, including surgery, radiofrequency ablation, transcatheter arterial chemoembolization (TACE), radiotherapy (except for radiotherapy at local appropriate doses for the relief of symptoms that does not affect blood pictures in patients with bone metastases);
4. Patients who have not received chemotherapy for relapsed or metastatic nasopharyngeal carcinoma (except for patients whose disease progressed more than 6 months after the receipt of neoadjuvant chemotherapy, adjuvant chemotherapy, or radical concurrent chemoradiotherapy);
5. The Eastern Cooperative Oncology Group (ECOG) score: 0~1 point (see Annex 1);
6. Expected survival ≥12 weeks;
7. Patients who have at least one measurable lesion according to Response Evaluation Criteria in Solid Tumors (RECIST 1.1), and the lesion should not have received radiotherapy or other local treatment;
8. The function of vital organs meet the following requirements (it is not allowed to use any blood component, cell growth factors, white blood cell-increasing drugs, platelet-increasing drugs, and drugs to correct anemia within 14 days prior to the first dose of the study drug);
    a) Absolute neutrophil count (ANC) ≥ 1.5×10^9/L;
    b) Platelet count ≥ 100×10^9/L;
    c) Hemoglobin ≥ 9 g/dL;
    d) Serum albumin ≥ 2.8 g/dL;
    e) Total bilirubin ≤ 1.5 × ULN, ALT, AST and/or AKP ≤ 2.5 × ULN; if there is liver metastasis, ALT and/or AST ≤ 5 × ULN; if there is liver metastasis or bone metastasis, AKP ≤ 5 × ULN ;
    f) Serum creatinine ≤1.5 × ULN or creatinine clearance >60 mL/min (Cockcroft-Gault, see annex II);

g) Activated partial thromboplastin time (APTT) and international normalized ratio (INR) ≤ 1.5 × ULN (patients on stable doses of anticoagulant therapy such as low molecular weight heparin or warfarin whose INR is within the expected therapeutic range of anticoagulants can be screened);

9. Female subjects with childbearing potential and male subjects whose partners are women of childbearing age should take one medically recognized contraceptive measure (IUD, birth control pills or condoms) during the study treatment, period, at least 3 months after the last dose of camrelizumab/placebo, and at least 6 months after the last use of chemotherapy;

10. Subjects who are voluntary to join the study, sign the informed consent form, have good compliance and cooperate with follow-up.

4.2 Exclusion Criteria

Patients in line with any of the following conditions may not enter the study.

1. Locally advanced patients, who can receive radical treatment such as surgery, radical radiotherapy or radical radiochemotherapy, will not be screened;

2. Patients with a past history of allergy to any component of camrelizumab, gemcitabine, cisplatin and other platinum drugs;

3. Patients with clinically symptomatic central nervous system metastases such as cerebral edema and requiring hormone intervention, or progression of brain metastases. Patients who have previously received brain or meningeal metastasis treatment, if they are stable both as shown by MRI and clinically (do not require > 10 mg/day prednisone or equivalent dose of hormone therapy), can be included;

4. Patients previously or concurrently suffering from other malignant tumors (except for malignant tumors which have been cured with cancer-free survival of more than 5 years, such as cutaneous basal cell carcinoma, cervical carcinoma in situ, papillary thyroid carcinoma, etc.);

5. Patients with uncontrolled cardiac clinical symptoms or diseases, such as: (1) NYHA grade II or higher heart failure, (2) unstable angina, (3) myocardial infarction that has occurred in the past 1 year, (4) supraventricular or ventricular arrhythmia with clinical significance and requiring clinical intervention;

6. Patients who have received any of the following treatments:

   a) Patients who have previously received anti-PD-1, anti-PD-1 antibody or anti-CTLA-4 antibody treatment;

   b) Patients who have used any investigational drugs within 4 weeks prior to the first dose of the study drug;

   c) Patients who are concurrently enrolled into another clinical study, unless the study is observational (non-interventional) clinical study or interventional clinical study follow-up;

   d) Patients who received the last dose of anti-cancer treatment (including chemotherapy, radiotherapy, targeted therapy, etc.) within ≤ 4 weeks prior to the first dose of the study drug;

   e) Subjects who need to receive systematic treatment with corticosteroids (>10 mg prednisone equivalent dose) or other immunosuppressive agents, except for the use of corticosteroids for local inflammation and prevention of allergies and nausea and vomiting. Other special cases require communication with the sponsor. In the absence of active autoimmune disease, inhaled or topical steroids and adrenal cortical hormone replacement therapy with at a dose >10 mg/day therapeutically effective dose of prednisone are allowed;

f) Patients who have received inoculation of tumor vaccines or have received live vaccines within 4 weeks prior to the first dose of the study drug;

g) Patients who have undergone major surgery or had a severe trauma within 4 weeks prior to the first dose of the study drug;

7. Patients whose toxicity due to previous anti-tumor treatment has not recovered to CTC AE ≤ grade 1 (except for hair loss, and sequelae of previous platinum treatment-related neurotoxicity) or the level prescribed by the inclusion/exclusion criteria;

8. Patients who have developed severe infection (CTC AE > grade 2) within 4 weeks prior to the first dose of the study drug, such as severe pneumonia, bacteremia and complication of infection that require hospitalization; patients whose baseline chest imaging findings suggest active pulmonary inflammation, or patients with symptoms and signs of infection within 2 weeks prior to the first dose of the study drug or need to be treated with oral or intravenous antibiotics (excluding prophylactic use of antibiotics);

9. Patients with active autoimmune diseases or a history of autoimmune diseases (e.g., interstitial pneumonia, colitis, hepatitis, pituitary inflammation, vasculitis, nephritis, hyperthyroidism and hypothyroidism, including but not limited to these diseases or syndromes); but excluding autoimmune-mediated hypothyroidism using a stable dose of thyroid replacement hormone therapy; type I diabetes using a stable dose of insulin; patients with vitiligo or cured childhood asthma/allergy that does not require any intervention after adulthood;

10. Patients with a history of immunodeficiency, including HIV positive, or other acquired or congenital immunodeficiency diseases, or a history of organ transplantation and a history of allogeneic bone marrow transplantation;

11. Patients with a history of interstitial lung disease (excluding radiation pneumonitis without hormone therapy), or history of non-infectious pneumonia;

12. Patients with active tuberculosis infection found by medical history or CT examination, or patients with a history of active tuberculosis infection within 1 year prior to enrollment, or patients with a history of active tuberculosis infection more than 1 year ago who have not received standardized treatment;

13. Subjects who have active hepatitis B (HBV DNA ≥ 2000 IU/mL or 10^4 copies/mL), hepatitis C (positive hepatitis C antibody, and HCV-RNA is higher than the lower limit of detection of the analytical method);

14. Subjects known to have a history of psychotropic substance abuse, alcohol abuse or drug abuse;

15. Women during pregnancy or lactation;

16. At the discretion of the investigator, subjects who have other factors that may lead to forced termination of the study halfway, such as other serious illnesses (including mental illness) requiring concomitant treatment, severe laboratory test abnormalities, family or social factors, or conditions that may affect subject safety or test data collection.

4.3 Randomization Criteria

Subjects meeting the inclusion/exclusion criteria will be 1:1 randomized into test group or control group by the randomization system. Randomization will be stratified by liver metastasis (yes vs. no), whether the subject has received radical concurrent radiochemotherapy (yes vs. no), and ECOG score (0 vs. 1).

4.4 Lifestyle Requirements

4.4.1 Contraception

If the investigator thinks that a female subject herself or the partner of a male subject is at risk of pregnancy, the subject must use at least 1 highly effective method for contraception from the signing of informed consent form to at least 3 months after the last use of camrelizumab/placebo, and at least 6 months after the last use of chemotherapy. After consultation with the subject, the investigator or his designee selects 1 contraceptive measure from the following contraceptive methods and confirms that the subject has known how to correctly and continuously use the contraceptive method. At the time points listed in the trial flowchart, the investigator will inform the subject of the need for continuous and correct contraception (the subject should confirm that she/she has continuously and correctly 1 selected contraceptive measure). In addition, the subject should be aware that the investigator should be notified immediately if the selected contraceptive method is discontinued or once the subject or the subject's partner has a suspected or confirmed pregnancy.

Highly effective contraceptive methods refer to contraceptive methods with an annual failure rate of less than 1% if continuously and correctly used alone or in combination with other methods, including the following types:

1. Commonly used hormonal contraceptive methods associated with inhibition of ovulation (e.g., oral, insertion, injection, implantation, transdermal), which should meet the following conditions: female subjects or the partners of male subjects have continued using the method for a period of time, and confirmed it to be effective, and plan to continuously and correctly use it throughout the study period.
2. Properly placed IUD.
3. Male/female condom in combination with topical spermicides (ie, foam, gel, film, cream or suppository).
4. Male sterilization by vasectomy.
5. Bilateral tubal ligation/bilateral tubal resection or bilateral fallopian tube occlusion surgery (occlusion surgery has been confirmed effective by the relevant instrument).

4.5 Subjects' Withdrawal from the Study or Termination of Study Treatment

4.5.1 Criteria for Withdrawal from the Study

The reasons for subjects' withdrawal from the study may include:

1. The subject withdraws the informed consent, and rejects further follow-up;
2. The subject is lost to follow-up;
3. The subject is dead;
4. The sponsor terminates the study.

4.5.2 Criteria for Termination of Study Treatment

The criteria for subjects' termination of the study treatment are as follows:

1. The subject withdraws informed consent and refuses to continue receiving the study drug treatment;
2. As judged by the investigator, the subject’s clinical symptoms deteriorate/performance status declines;
3. The subject is judged as progressive disease according to RECIST 1.1 criteria. Subjects meeting the criteria for clinically stable (see Section 0) can continue the medication until confirmed PD or the investigator judges that the subject can no longer clinically benefit;
4. Camrelizumab/placebo has been cumulatively used for up to 2 years (without imaging progression);
5. The toxicity is intolerable, including the occurrence of any clinical AE, laboratory test abnormalities or other medical conditions. If a subject cannot tolerate camrelizumab or chemotherapy toxicity, continued use of the drug with intolerable toxicity should be terminated;
6. There are other situations where the investigator thinks it necessary to terminate the study drug treatment;
7. The subject is pregnant;
8. The subject is lost to follow-up;
9. The subject is dead;
10. The sponsor terminates the study.

4.5.3 Steps for Withdrawal from or Termination of Treatment

It is required to perform effectiveness and safety examinations of subjects according to the provisions on withdrawal from the study treatment and safety follow-up visits in the protocol, and comprehensively record their AE and outcomes. The investigator may suggest or provide a new or alternative treatment method to subjects depending on their actual situation. Non-PD patients should continue follow-up and undergo imaging evaluation until the subject’s PD, the initiation of a new anti-tumor treatment, withdrawal of informed consent, loss to follow-up or death.

If the subject rejects further visits at the study site, the status of his/her survival should continue to be tracked unless the subject withdraws the consent for disclosure of further information or continued contact. In this case, no further study evaluation should be conducted and no further information should be collected. The sponsor may retain and continue to use all information before the subject withdraws his/her informed consent unless the subject has requested that the collected information be withdrawn as well.

4.6 Early Termination or Suspension of the Study

The study may be early terminated or suspended on sufficient grounds. This may be due to the decision of the regulatory body, the change of opinion of the Ethics Committee, the efficacy or safety issues of the study drug, or the judgment of the sponsor. In addition, Hengrui reserves the right to stop developing camrelizumab at any time. The party deciding to suspend/terminate the study will issue a written notice recording the reason for the termination or suspension of the study to the investigator, the sponsor and the regulatory body. The investigator should immediately inform the Ethics Committee and provide relevant reasons.

Reasons for early termination or suspension of the study may include:

- The study brings definite unexpected, major or unacceptable risks to subjects.
- Existing efficacy results support early termination of the study.
- The protocol requirements are poorly followed;
- The data are incomplete or undetectable.
- The study results have no utilization value.

Once the abovementioned drug safety, protocol compliance and data quality issues causing study suspension are solved and the consent from the sponsor, the Ethics Committee or the China National Drug Administration (CNDA) is obtained, the study can be continued.

4.7 Definition of End of Trial

End of trial is defined as follows: the trial ends when the final analysis is completed.

5 Immunogenicity Studies

5.1 Collection and Processing of Blood Samples

5.1.1 Time of Collection of Blood Samples

C1D1, C2D1, C4D1, C6D1, C9D1, once before each administration of camrelizumab/placebo, once before administration every 4 cycles thereafter, when the subject withdraws from the study treatment/once each at 30 days, 60 days and 90 days after the last dose (according to visit arrangement, if applicable).

5.1.2 Processing and Preservation of Blood Samples

4-6 mL of venous blood is collected at each of the abovementioned time points as blood samples, and stored in a serum separation tube. The collected serum is transferred to 4 cryotubes (averaged to 3 test tubes, 1 for testing the immunogenicity [ADA], 1 for testing the trough drug concentration, and 1 for testing the neutralizing activity of antibody, as well as 1 backup tube). The cryotubes are placed in a low temperature refrigerator and can be stored for 6 months at -60~-80 °C and 1 month at -20 °C until shipment to the central laboratory for testing. The specific operation details are described in the *Laboratory Manual*.

5.2 Mailing of Clinical Samples

Test tube samples will be first sent out in dry ice storage. Backup tube samples will be sent out after the bioanalytical laboratory confirms that the test tube samples have been received. The mailing frequency, mailing information and other details are described in the *Laboratory Manual*.

6 Study Medication

6.1 Overview of Test Drug and Medication for the Control Group

6.1.1 Method of Drug Acquisition

The test drug will be provided by the sponsor in uniform package, which should have proven qualified after inspection (see the corresponding inspection report).

6.1.2 Dosage Form, Appearance and Package of Drugs

Study drug: Camrelizumab for Injection (not marketed)
Manufacturer: Suzhou Shengdiya Biopharmaceutical Co., Ltd.
Dosage form: Freeze-dried powder
Strength: 200 mg, packaged in 20 mL penicillin bottles.

Batch number: See the drug inspection report
Usage: Intravenous infusion
Shelf life: The shelf life is tentatively 2 years from the date of manufacture.
Storage conditions: Store sealed in shade in a 2-8 ºC medical refrigerator. No freezing.

Study drug: Placebo
Manufacturer: Suzhou Shengdiya Biopharmaceutical Co., Ltd.
Dosage form: Freeze-dried powder
Strength: packaged in 20 mL penicillin bottles.
Batch number: See the drug inspection report
Usage: Intravenous infusion
Shelf life: The shelf life is tentatively 2 years from the date of manufacture.
Storage conditions: Store sealed in shade in a 2-8 ºC medical refrigerator. No freezing.

Study drug: Gemcitabine
Manufacturer: Jiangsu Hansoh Pharmaceutical Group Co., Ltd.
Dosage form: Freeze-dried powder
Strength: 1.0 g
Batch number: See the package insert
Usage: Intravenous infusion
Shelf life: 36 months
Storage conditions: Store sealed in a dry place

Study drug: Cisplatin
Manufacturer: Jiangsu Hansoh Pharmaceutical Group Co., Ltd.
Dosage form: Injection
Strength: 6 mL:30 mg
Batch number: See the package insert
Usage: Intravenous infusion
Shelf life: Tentatively 24 months
Storage conditions: Store sealed in shade

6.1.3 Drug Preservation and Stability

The investigator or his authorized representative (e.g., pharmacist) is responsible for ensuring that all study drugs are stored in a controlled safe area that meets the storage conditions and that their storage complies with applicable regulatory requirements.

The study drugs should be stored according to the storage conditions listed in Section 6.1.2. When the protocol is inconsistent with other information, the storage conditions on the label of camrelizumab/placebo should prevail, and the storage conditions on the package insert for marketing of gemcitabine and cisplatin should prevail.

The study site must be able to measure and record daily maximum and minimum temperatures at all storage locations (e.g., frozen, refrigerated or room temperature). The recording cycle should start from the receiving of the drugs until the last subject of the study site completes the last study treatment. Even if there is a continuous monitoring system, the study site should also have a record log to ensure the correct storage temperature. Temperature monitoring devices and storage devices (e.g. refrigerators) should be checked regularly to ensure that they are normally functioning.

Once the storage conditions deviate from what is indicated by the drug label, storage instructions or package insert, it should be promptly reported. The study site should take active measures to return the drug to the storage conditions specified in the label or package insert as soon as possible, and report the temperature deviation and the measures taken to the sponsor.

Study drugs affected by temperature deviations should be temporarily isolated until the sponsor allows them to continue to be used, and it does not fall into protocol deviations. Continued use of affected study drugs without the sponsor's permission is a protocol deviation. The sponsor will provide the study site with the specific steps for reporting of temperature deviations.

6.1.4 Drug Preparation

The drugs used in this study are all administered by intravenous infusion. Therefore, the drugs should be prepared by qualified or experienced research personnel such as study nurses. Camrelizumab/placebo is preservative-free, and should be dispensed by aseptic operation. Please refer to the drug manual for drug preparation. Both gemcitabine and cisplatin are marketed drugs, and the drug preparation is carried out according to their package insert.

The drug dispensing process in this study is blind, and designated study nurses will be responsible for dispensing. The personnel should carefully check the doctor's order during the dispensing, and use the drug of specified number for preparation according to the system's grouping requirements. Drug dispensing should be operated in a separate treatment room as far as possible and other people should not be allowed to enter to prevent unblinding. The dispensed test drug and placebo should be guaranteed to be identical in terms of appearance, packaging, label and other characteristics and then delivered to the administering nurses. The drug dispensing nurses must not communicate with other blinded personnel in this trial regarding the drug dispensing process and do not participate in the subject's administration operation as far as possible.

6.1.5 Drug Usage

Study drugs are administered by specified administering study nurses.

Test group: The method of administration is shown in Table 2 below. Camrelizumab is combined with gemcitabine and cisplatin, and the chemotherapy lasts for 4-6 cycles. If camrelizumab, gemcitabine or cisplatin has been discontinued for toxicity reasons, other study medications may continue to be used in the remaining cycles until the subject meets the criteria for withdrawal from the study treatment.

Control group: The method of administration is shown in Table 2. Placebo is combined with gemcitabine and cisplatin, and the chemotherapy lasts for 4-6 cycles. If placebo, gemcitabine or cisplatin has been discontinued for toxicity reasons, other study medications may continue to be used in the remaining cycles until the subject meets the criteria for withdrawal from the study treatment.

If chemotherapy is not given in a certain cycle, the cycle will not be counted in the cycles of combination therapy. After the completion of combination therapy, the subject will continue camrelizumab/placebo monotherapy until he/she meets the criteria for withdrawal from the study treatment.

Table 2 Method of Administration

	Camrelizumab/placebo	Gemcitabine	Cisplatin
Dosage and route of administration	200 mg/placebo IV	1000 mg/m^2, IV	80 mg/m^2, IV
Infusion rate	No less than 20 min, no more than 60 min, including the washing stage	About 30 min or according to the study site's clinical practice	About 60 min or according to the study site's clinical practice
Pretreatment before administration	No pretreatment required	Pretreatment before administration is given according to the study site's clinical practice	Pretreatment before administration is given according to the study site's clinical practice
Time of administration	D1	D1, D8	D1
Dosing sequence of combination therapy	After the infusion of camrelizumab/placebo, gemcitabine and cisplatin are successively given after an interval of at least 30 min.		
Dosing frequency of combination therapy	Every 3 weeks as 1 dosing cycle (4-6 cycles, because of no less than 3 cycles in subjects with intolerable toxicity)		
Dosing frequency of maintenance monotherapy	Every 3 weeks as 1 dosing cycle		
Total duration of administration of camrelizumab/placebo	Up to 2 years		

Some subjects may have a temporary tumor outbreak within the first few months after starting immunotherapy, followed by a disease response, so the subjects are allowed to continue camrelizumab/placebo combined with chemotherapy or camrelizumab/placebo monotherapy after the first PD (based on the RECIST 1.1 criteria).

A tumor outbreak includes any of the following conditions:

- Deterioration of the original target lesion;
- Deterioration of the original non-target lesions;
- Appearance of new lesions.

The investigator can decide whether the subject continues to receive the study treatment based on the overall clinical condition of the subject, including performance status, clinical symptoms, and laboratory test results. If the investigator determines that the subject is clinically stable and can continue to have clinical benefits, and the sponsors agrees after discussion, the subject can continue to receive treatment and undergo a tumor re-assessment at least 4 weeks later by comprehensively referring to the iRECIST and RECIST 1.1 criteria. If confirmed to be non-confirmed PD, the subject can continue the study treatment. If the subject is confirmed to be confirmed PD, the treatment will be terminated unless the investigator believes that the subject can continue to have clinical benefits. The investigator should discuss with the sponsor again. After consent, the subject can sign the informed consent form for continued treatment after PD, and continue the medication until the investigator judges that the subject cannot continue to have clinical benefits. For clinically unstable subjects, the treatment should be discontinued after the first evaluation of PD, without the need for repeated imaging examination to confirm PD. As this study is a double-blind design, continued medication after PD requires the investigator to make a cautious judgment. For subjects who cannot be clearly judged, the investigator may choose not to continue the medication after PD.

Clinically stable is defined below:

- The subject's performance status does not significantly decline, and there is no significant worsening of the tumor-related symptoms;
- Rapid PD does not occur;
- There are no progressive tumors (e.g., spinal cord compression) requiring additional emergency medical intervention at important anatomical sites.

Reference should be made to the criteria in Table 3 for confirmation of PD.

Table 3 Criteria for Confirmation of PD

	**Situations where PD can be confirmed (any of the following conditions)**	**Situations where PD cannot be confirmed (all the following conditions are met)**
Target lesion	The absolute value of tumor burden is increased by ≥5 mm compared with the first progression.	The absolute value of tumor burden is increased by <5 mm compared with the first progression.
Non-target lesion	Compared with that at the time of the first progression, the non-target lesion has persistently progressed (qualitative).	Compared with that at the time of the first progression, there is no definite progression (qualitative).
New lesion	(1) Compared with the first progression, a new lesion appears.<br>(2) If a new lesion has previously appeared, the new lesion increases, or other new lesions appear.	(1) Compared with the first progression, there are no other new lesions.<br>(2) If a new lesion has previously appeared, the new lesion is stable or shrinks.

For subjects evaluated as PD for the first time, no matter whether they continue the study treatment after PD or not, the date when the investigator evaluates initial progression will be used in all statistical analyses containing progression information.

6.1.6 Dose Adjustment and Delay

6.1.6.1 Dose Adjustment

6.1.6.1.1 Dose Adjustment of Camrelizumab/Placebo

The AE related to camrelizumab/placebo may be immunologically related and may occur shortly after the first dose or several months after the last dose. If the situation listed in Table 4 occurs, the administration of camrelizumab/placebo should be suspended. If the investigator considers the benefit/risk ratio of the subject during the clinical operation and believes that the operation listed in Table 4 cannot be performed or encounters situations not listed in Table 4, the investigator must suspend or resume the administration of camrelizumab/placebo, and discuss and decide together with the sponsor.

Table 4 Criteria for Dose Adjustment of Camrelizumab/Placebo

**Drug-related immune AE**	**Grade for suspension of administration**	**Time to restart the treatment**	**Termination of medication**
Diarrhea/colitis	2-3	The event recovers to grade 0-1 and the dose of corticosteroids is reduced to 10 mg or less of prednisone or its equivalent dose	The event is not relieved within 12 weeks after the last dose, or the dose of corticosteroids cannot be reduced to 10 mg or less of prednisone or its equivalent dose within 12 weeks
	4	Terminate the medication	Terminate the medication
Elevated AST, ALT or bilirubin	2	The event recovers to grade 0-1 and the dose of corticosteroids is reduced to 10 mg or less of prednisone or	The event is not relieved within 12 weeks after the last dose

		its equivalent dose	
	3-4	Terminate the medication	Terminate the medication
Hyperthyroidism	3	The event recovers to grade 0-1 and the dose of corticosteroids is reduced to 10 mg or less of prednisone or its equivalent dose	The event is not relieved within 12 weeks after the last dose, or the dose of corticosteroids cannot be reduced to 10 mg or less of prednisone or its equivalent dose within 12 weeks
	4	Terminate the medication	Terminate the medication
Hypothyroidism		After a thyroid hormone replacement therapy is started, the treatment can continue	After a thyroid hormone replacement therapy is started, the treatment can continue
Pneumonia	2	The event recovers to grade 0-1 and the dose of corticosteroids is reduced to 10 mg or less of prednisone or its equivalent dose	The event is not relieved within 12 weeks after the last dose, or the dose of corticosteroids cannot be reduced to 10 mg or less of prednisone or its equivalent dose within 12 weeks
	3-4	Terminate the medication	Terminate the medication
Immune-related hypophysitis*	Moderate	The event recovers to grade 0-1; the treatment with camrelizumab/placebo can continue after an endocrine replacement therapy is started	The event is not relieved within 12 weeks after the last dose, or the dose of corticosteroids cannot be reduced to 10 mg or less of prednisone or its equivalent dose within 12 weeks
	Severe	Terminate the medication	Terminate the medication
Type I diabetes (if newly developed) or hyperglycemia	Newly developed type I diabetes or grade 3-4 hyperglycemia with evidence of beta cell failure	After clinical and metabolic conditions are stable	Continue the treatment with camrelizumab/placebo
Renal failure or nephritis	2	The event recovers to grade 0-1 and the dose of corticosteroids is reduced to 10 mg or less of prednisone or its equivalent dose	The event is not relieved within 12 weeks after the last dose, or the dose of corticosteroids cannot be reduced to 10 mg or less of prednisone or its equivalent dose within 12 weeks
	3-4	Terminate the medication	Terminate the medication
Infusion reaction	2	The symptoms disappear	When the medication is resumed after the symptoms disappear, the infusion rate should be 50% of the initial rate. If no complication occurs within 30 minutes, the infusion rate can be increased to 100% of the initial rate. The subject should be closely monitored. If the symptoms recur, do not infuse camrelizumab/placebo for the current treatment.
	3-4	Terminate the medication	Terminate the medication
Other drug-related AEs	3	The event recovers to grade 0-1 and the dose of corticosteroids is reduced to 10 mg or less of prednisone or its equivalent dose	The event is not relieved within 12 weeks after the last dose, or the dose of corticosteroids cannot be reduced to 10 mg or less of prednisone or its equivalent dose within 12 weeks
	4	Terminate the medication	Terminate the medication

Note: In the event of any recurrent grade 3 drug-related AE or any life-threatening event, the medication should be terminated.

For patients with liver metastasis and grade 2 AST or ALT elevation at baseline, if the AST or ALT is increased by ≥50% from baseline and remains so for at least 1 week, the treatment should be terminated.

For subjects experiencing intolerable or persistent grade 2 drug-related AE, the investigator may suspend camrelizumab/placebo as appropriate; if the persistent grade 2 adverse reaction fails to recover to grade 0-1 within 12 weeks after the last dose, the medication should be terminated.

* Hypophysitis is divided into three grades: mild (fatigue, anorexia), moderate (headache, mood change), and severe (visual disorder, adrenal insufficiency).

6.1.6.1.2 Dose Adjustment of Gemcitabine and Cisplatin

The dose adjustment of gemcitabine and cisplatin should be performed as decided by the investigator according to routine clinical practice. This chapter is for the investigator's reference. The criteria for dose adjustment of gemcitabine and cisplatin and criteria for treatment/resumed treatment can refer to Table 5, Table 6 and Table 7. If the subject still cannot tolerate the toxicity after dose reduction, the chemotherapeutic medication should be terminated.
The dose adjustment levels for chemical drugs are shown in Table 5 below.

Table 5 Dose Adjustment Levels for Chemical Drugs

	Starting dose	Reduced by one dose level	Reduced by two dose levels
Gemcitabine	1000 mg/m^2	750 mg/m^2	500 mg/m^2
Cisplatin	80 mg/m^2	60 mg/m^2	40 mg/m^2

Before starting chemotherapy in any cycle, all the following conditions should be met. Otherwise, a delay in administration is required:

- Absolute neutrophil value (ANC) ≥1.5×10^9/L;
- Platelet count ≥100×10^9/L;
- Hemoglobin ≥90 g/L.

Gemcitabine is injected intravenously on Day 1 and Day 8 of chemotherapy. The subject can continue the injection on Day 8 if his/her ANC is ≥1.0×10^9/L and platelet count ≥75×10^9/L before chemotherapy on Day 8. Otherwise, it is necessary to delay the administration of gemcitabine, but the delay cannot exceed 5 days.
The recommended dose adjustments for chemotherapy-related hematological toxicities are shown in Table 6 below:

Table 6 Dose Adjustment of Chemical Drugs Based on the Chemotherapy-related Hematological Toxicities of the Previous Cycle

Chemotherapy-related events	Gemcitabine	Cisplatin
ANC<0.5×10^9/L for more than 5 days	Reduced by one dose level	Reduced by one dose level
Febrile neutropenia (ANC<0.5×10^9/L, body temperature ≥38.3 ℃ once or body temperature ≥38 ℃ continuously for more than 1 hour)	Reduced by one dose level	Reduced by one dose level
Platelet count<50×10^9/L, with event hemorrhage	Reduced by one dose level	Reduced by one dose level
Platelet count<25×10^9/L	Reduced by one dose level	Reduced by one dose level
Hemoglobin<65g/L	Reduced by one dose level	Reduced by one dose level

If the third above-mentioned toxic reaction still occurs after two reduction, chemotherapy should be stopped.

The recommended dose adjustments for chemotherapy-related non-hematological toxicities are shown in Table 7 below:

Table 7 Dose Adjustment of Chemical Drugs Based on the Chemotherapy-related Non-hematological Toxicities of the Previous Cycle

Adverse event	CTCAE grade	Gemcitabine	Cisplatin
Nausea or vomiting	Grade 3 or 4	Original dose	Reduced by one dose level
Diarrhea	Grade 3 or 4	Reduced by one dose level	Reduced by one dose level
Rash	Grade 3 or 4	Reduced by one dose level	Original dose
Ototoxicity	Grade 2	Original dose	Suspend the medication*
	Grade 3 or 4	Original dose	Stop the medication
Neurotoxicity	Grade 2	Original dose	Reduced by one dose level
	Grade 3 or 4	Original dose	Stop the medication
Other non-hematological toxicity	Grade 3 or 4	Reduced by one dose level	Reduced by one dose level

*If the subject experiences ototoxicity, the use of cisplatin should be suspended until the audiometric analysis shows that the hearing acuity is within the normal range.

Nephrotoxicity:
Creatinine clearance (CrCl) is calculated using the Cockcroft-Gault formula based on standard body weight (Annex 2). Before dosing in each treatment cycle, the Cockcroft-Gault formula must be used to assess the creatinine clearance. For Cycle 2 chemotherapy and chemotherapy after Cycle 2, if the creatinine clearance (Ccr) is <60 ml/min, blood creatinine should be tested after 24 hours of hydration and the Cockcroft-Gault formula will be used to calculate the creatinine clearance. The dose of cisplatin should be adjusted according to the following criteria. If the 24-hour urine is collected to estimate Ccr, the results calculated by the above formula will no longer be used, and measures will be taken according to the following methods (if the formula calculation results and the urine collection-based estimates are very different, consider using the results which are more accurate:

- If the creatinine clearance is ≥60 ml/min, maintain the original dose of cisplatin;
- If the creatinine clearance is 41-59 ml/min, the value of cisplatin dose (mg/m^2) is the same as the Ccr value (ml/min). (For example: if the Ccr is 45 ml/min, the applied dose of cisplatin is 45 mg/m^2);
- If the creatinine clearance is ≤40 ml/min, discontinue cisplatin in this chemotherapy cycle, and perform adjustments according to specific creatinine clearance data in the subsequent cycles.

6.1.6.2 Delay in Administration

The dosing window period is the scheduled dosing date± 3 days. Subsequent dosing date will be calculated according to the last actual dosing date.

If the investigator determines that the toxicity is mainly caused by one of the test drugs, a single drug reduction or suspension is acceptable; if it is unclear whether the toxicity is caused by two or more test drugs, all drugs that are considered to be related can be reduced or suspended.

During the combination therapy, if the delay is expected to be more than 2 weeks due to the toxicity of chemotherapy, only careliuzumab/placebo will be given until the toxicity returns to the criteria for chemotherapy dosing, and then the combination of chemotherapy with camrelizumab/placebo will be restarted. Chemotherapy is allowed to be suspended for 6 consecutive weeks. If the suspension exceeds 6 weeks, the chemotherapy will be terminated; if the delay is expected to be more than 2 weeks due to the toxicity of camrelizumab/placebo, then chemotherapy is only given until the toxicity returns to the criteria for camrelizumab/placebo

dosing, and then the combination of chemotherapy with camrelizumab/placebo will be restarted. Camrelizumab/placebo is allowed to be suspended for up to 12 consecutive weeks. If the suspension exceeds 12 weeks, camrelizumab/placebo will be terminated; if delay is required for toxicity reasons and it is expected that the toxicity can recover to the re-dosing criteria within 2 weeks, the three drugs need to be delayed at the same time. If the Day 8 dosing of gemcitabine is delayed for more than 7 days due to toxicity, skip the Day 8 dosing and enter the next cycle. The chemotherapy cycle and the camrelizumab/placebo cycle should be kept as synchronized as possible by adjusting the allowed window period.

6.2 Drug Management, Distribution and Recovery

The management, distribution and recovery of the study drugs shall be in the charge of specific persons. The investigator must ensure that all study drugs are only applied to subjects participating in the clinical study, their dosage and usage should follow Section 0, the remaining drugs or expired drugs should be returned to the sponsor. It is prohibited to hand over the clinical medications to any non-participant of the clinical study.
When the drug is distributed to each site and proves qualified after inspection by the study site's personnel, a drug receiving sheet must be signed by two persons in duplicate, with the study site and the sponsor each holding one copy. For the recovery of remaining drugs and empty boxes, the two parties shall sign a drug recovery sheet. The distribution and recovery of each drug should be timely recorded in the specific record sheet.
The monitor is responsible for monitoring the supply, use, storage of clinical study medications and the handling process of remaining drugs.

6.2.1 Destruction of Study Drug

The sponsor or its authorized persons will destroy the study drug, and records shall be properly made on all destructions.

6.3 Accompanying Treatments

The drugs or vaccines expressly banned in the exclusion criteria are strictly prohibited throughout the study. If a subject has a concomitant disease and must use a banned drug, the subject may need to stop the study drug treatment and accept the banned drug. The investigator needs to discuss with the sponsor, and the investigator, the sponsor and the subject will jointly decide whether the subject continues to participate in the study treatment and/or accept the banned drug.

6.3.1 Allowed Concomitant Treatments

Topical use of corticosteroids, such as the eyes, nose, inside joints and inhalation, is allowed. Corticosteroid pretreatment before chemotherapy is allowed.

Subjects should be given optimal supportive care during treatment. The original hormone replacement therapy and the use of bisphosphonates for the treatment of bone metastases are allowed.

It is allowed to perform palliative treatment of local lesions that cause significant symptoms, such as bone metastases and esophageal lesions. Local radiotherapy or surgery may be considered, but the following conditions must be met, and the investigator shall discuss with the sponsor prior to initiation of palliative local treatment.

1. For subjects in need of topical treatment due to aggravation of symptoms during the study period, the investigator must determine whether the subjects have PD; PD subjects must meet the criteria for continued treatment after progression;

2. Locally treated lesions cannot be target lesions.

All concomitant medications should be recorded in the eCRF. All concomitant medications should be recorded from within 30 days prior to the signing of informed consent to the initiation of a new anti-tumor treatment. Thereafter, only concomitant medications for adverse events that are related to the study drug need to be recorded.

6.3.2 Banned Concomitant Treatments

- Systemic anti-tumor treatment, including but not limited to chemotherapy and immunotherapy, targeted therapy, biotherapy, modern Chinese medicine preparations that have been approved by the CNDA for marketing used for anti-tumor treatment (see Annex IV), and immunomodulators with an auxiliary anti-tumor effect (e.g., thymosin, lentinan, interleukin-12, etc.) that are not specified in the protocol.
- Inoculation of live vaccines within 4 weeks prior to the first dose of the study drug and during participation in the study, including but not limited to measles, mumps, rubella, chickenpox, yellow fever, rabies, BCG and typhoid vaccines. Injectable inactivated virus vaccines for seasonal flu are allowed, but live attenuated flu vaccines for intranasal use are not allowed;
- Except for alleviating the symptoms caused by immunological causes, the long-term systemic use of glucocorticoids for any other purpose needs to be discussed with the sponsor. Short-term (<3 weeks) and low-dose (≤10 mg prednisone equivalent) use of corticosteroids to treat non-autoimmune diseases and prophylactic use of corticosteroids (e.g., to avoid allergy to contrast agents) are allowed;
- For other drugs that should be banned for gemcitabine and cisplatin, please refer to their package inserts for marketing.

6.3.3 Supportive Care

6.3.3.1 Guide for Supportive Treatment for Camrelizumab/Placebo

Subjects should receive appropriate supportive treatment measures deemed as necessary by the investigator. Supportive treatment measures for the treatment of potentially immune-related AEs are listed below, including oral or intravenous corticosteroids, and the use of other anti-inflammatory drugs in the absence of improvement in symptoms after the use of corticosteroids. Steroid tapering may take several cycles, as symptoms may worsen during the tapering process. Other causes that may require additional supportive treatment, such as metastatic disease or bacterial or viral infections, should be excluded as much as possible. When the investigator confirms that the AE is related to camrelizumab/placebo, the supportive treatments listed below may be used; if the AE is not related to camrelizumab/placebo, there is no need to use the supportive treatments listed below.

1. Capillary hyperplasia

If a subject develops capillary hyperplasia which occurs at a vulnerable site, local treatment such as surgery, laser ablation or liquid nitrogen freezing is recommended. For patients undergoing surgical resection, pathological examination reports should be collected as much as possible. Endoscopy and MRI examination are recommended for subjects with severe or long-lasting capillary hyperplasia to confirm whether the visceral mucosa is involved.

2. Diarrhea/colitis

Subjects' symptoms and signs of enterocolitis (e.g., diarrhea, abdominal pain, blood in the stool or mucous stool, with or without fever) and intestinal perforation (e.g., peritoneal endometriosis and intestinal obstruction) should be carefully monitored.

- All subjects with diarrhea/colitis should be recommended to drink a sufficient amount of fluid. If they cannot take in a sufficient amount of fluid taken orally, the fluid and electrolyte should be infused intravenously. For grade 2 or above diarrhea, gastroenterology consultation and endoscopy should be considered to confirm or exclude colitis;
- For patients with grade 2 diarrhea/colitis in whom glucocorticoid intravenous therapy for 3-5 days proves effective can switch to oral hormone therapy, and reduce the dose gradually in 8-12 weeks;
- For grade 3 or 4 diarrhea/colitis, the subject can receive intravenous steroids and then orally administer high doses of steroids; patients who have proven glucocorticoid therapy ineffective can also choose immunosuppressants (e.g., infliximab, vedolizumab, mycophenolate mofetil (MMF), etc.);
- After the symptoms are improved to grade 1 or below, the steroids should be reduced and continued for no less than 4 weeks.

3. Elevated AST, ALT or bilirubin

- For grade 2 events, serum aminotransferase and total bilirubin levels should be detected twice a week; if grade 2 elevation of serum aminotransferase and total bilirubin levels persists for more than 1-2 weeks, after the exclusion of other pathogenic factors, the subject should receive intravenous or oral corticosteroid therapy with (methyl) prednisolone at a dose of 1 mg/(kg*d) or other equivalent drugs. Once the condition is improved, the dose of corticosteroids should be gradually reduced, and then the subject can continue to use camrelizumab. If the condition deteriorates or is not improved after the use of corticosteroids, increase the dose of corticosteroids to 2 mg/(kg*d) (methyl) prednisolone or use other equivalent drugs, and permanently discontinue camrelizumab;
- For grade 3-4 events, the subject should receive intravenous corticosteroid therapy with (methyl) prednisolone at a dose of 1-2 mg/(kg*d) or other equivalent drugs. If the patient has no response to corticosteroids within 2-3 days, MMF 1000 mg twice a day should be additionally used. For refractory cases, consultation with hepatologists can be requested and liver tissue biopsy may be considered;
- After the symptoms are improved to grade 1 and below, the steroids should be reduced and continued for no less than 4 weeks.

4. Hyperthyroidism/Hypothyroidism

Thyroid disease may occur at any time during treatment. It is thus necessary to monitor changes in the patient's thyroid function (when the treatment starts, and regularly during the treatment period) and clinical symptoms and signs of thyroid disease.

- For grade 2 hyperthyroidism, it is recommended to use a non-selective beta-blocker (e.g., propranolol) as the initial treatment. If the subject presents with thyroiditis accompanied by pain, treatment with prednisolone 0.5 mg/kg should be considered. If the symptoms still are not improved, camrelizumab treatment should be interrupted until the symptoms disappear for consideration of re-medication;
- For grade 3-4 hyperthyroidism, the subject should start with intravenous corticosteroids and then receive oral corticosteroids. After the symptoms are improved to grade 1 or below, the steroids should be reduced and continued for no less than 4 weeks. Proper hormone replacement therapy may be required during the steroid tapering process;
- For grade 2-4 hypothyroidism, thyroid hormone replacement therapy (e.g., levothyroxine) can be chosen. If the patient has exhaustion or other hypothyroidism-related chief complaints, even for subclinical hypothyroidism, hormone replacement therapy (thyroxine 0.5-1.5 μg/kg) should be considered and maintained for a long term.

5. Pneumonia

- For subjects with mild to moderate symptoms (grade 2) and severe/life-treatening symptoms (grade 3-4), prednisolone 1 mg/(kg•d) and 2-4 mg(kg•d) are recommended, respectively.
- The condition should be closely assessed once the treatment starts; for grade 2 pneumonia, the clinical symptoms should be assessed once every 2-3 days, and concurrent imaging assessment is recommended; for grade 3-4 pneumonia, the clinical symptoms and imaging should be assessed after 2 days of treatment; if there is no sign of improvement, the subject should additionally use an immunosuppressive agent such as infliximab, MMF or cyclophosphamide as early as possible.
- If the subject receives long-term steroid medication, attention should be paid to supplementation of vitamin D and calcium and prophylactic use of antibiotics.

6. Immune-related hypophysitis

- Subjects with mild symptoms can continue ICPis treatment, and be given appropriate hormone replacement therapy (hydrocortisone, thyroxine);
- Subjects with moderate and above symptoms should immediately interrupt ICPis treatment, and those with moderate symptoms should orally take prednisolone 0.5~1 mg/kg;
- Subjects with severe symptoms should be treated with intravenous (methyl) prednisolone 1 mg/kg. According to the control of symptoms, the dose can be gradually reduced to 5 mg, but hormone therapy cannot be stopped;
- As hypophysitis involves the inter-regulation among a number of endocrine hormones of the body, consultation with and guidance by endocrinologists is highly recommended.

7. Type I diabetes

- For type I diabetes and grade 3-4 hyperglycemia with metabolic acidosis or ketouria, insulin replacement therapy is recommended, and the subject's blood glucose and full-set metabolic indicators, urinary ketone, glycosylated hemoglobin, and C-peptide should be evaluated.

8. Renal failure or nephritis

- For grade 2 events, corticosteroid therapy should be accepted;
- For 3-4 events, systemic administration of corticosteroids should be accepted;
- After the symptoms are improved to grade 1 or below, the steroids should be reduced and continued for no less than 4 weeks.

9. Infusion reactions

The recommendations for the treatment of infusion reactions are shown in Table 8.

Table 8 Recommendations for the Treatment ofInfusion Reactions

CTCAE grade	Clinical symptoms	Clinical treatment	Camrelizumab/placebo treatment
Grade 1	Mild transient reactions	Bedside observation, close monitoring until recovery. Prophylactic medication is recommended prior to subsequent infusion: diphenhydramine 50 mg, or equivalent and/or acetaminophen 325-1000 mg, at least 30 min before camrelizumab/placebo administration.	Continue.
Grade 2	Moderate reactions, requiring treatment or suspension of administration, which can be quickly relieved after symptomatic treatment (e.g., antihistamines, non-steroidal anti-inflammatory drugs, anesthetics, bronchodilators, intravenous infusion, etc.)	Intravenous infusion of normal saline, diphenhydramine 50 mg IV or equivalent and/or acetaminophen 325-1000 mg;<br>Bedside observation, close monitoring until recovery.<br>According to clinical needs, consider corticosteroids or bronchodilators;<br>Record the infusion amount of the study drug in the original medical records;<br>Prophylactic medication is recommended prior to subsequent infusion: at least 30 min before camrelizumab/placebo administration, diphenhydramine 50 mg, or equivalent and/or acetaminophen 325-1000 mg. If necessary, cortisol hormone (at a dose equivalent to 25 mg of hydrocortisone).	Suspension. Restart the medication at 50% of the initial infusion rate after the symptoms disappear. If there is no complication within 30 min, the infusion rate can be increased to 100% of the original infusion rate. Closely monitor. If the symptoms relapse, the currently therapeutic camrelizumab/placebo will no longer be infused.
≥ grade 3	Grade 3: severe reactions, which are not quickly relieved after treatment and/or suspension of administration; or the symptoms relapse after remission; sequelae requiring hospitalization occur.<br>Grade 4: life-threatening	Immediately stop infusion of camrelizumab/placebo;<br>Start intravenous infusion of normal saline.<br>• Recommended bronchodilators: 1: 1000 adrenaline solution 0.2-1 mg via subcutaneous injection, or 0.1-0.25 mg 1: 10000 solution adrenaline via slow intravenous injection; if necessary, and/or diphenhydramine 50 mg + methylprednisolone 100 mg or equivalent via intravenous injection;<br>• Follow the guidelines of the study organization for the treatment of allergic reactions;<br>Bedside observation, close monitoring until recovery.	Discontinue the medication.

6.3.3.2 Guide for Supportive Treatment for Gemcitabine and Cisplatin

For specifics, refer to the package inserts or the study site's clinical practice.

In this study, cisplatin is a highly emetic drug, while gemcitabine is a lowly emetic drug. By referring to the NCCN Antiemetic Guidelines, it is recommended to use a prophylactic antiemetic regimen from the start of the first treatment cycle. This study suggests a combination of NK-1 receptor antagonists, 5-HT3 receptor antagonists, and dexamethasone for prophylactic antiemetics prior to chemotherapy. NK-1 receptor antagonists include aprepitant, valsartan, nepidine, and lorabitan. If aprepitant is chosen, the subject is advised to take 125 mg orally on the first day of the chemotherapy cycle and 80 mg on the second and third days of chemotherapy. 5-HT3 receptor antagonists include dolasetron, granisetron, ondansetron, palonosetron, and tropisetron. Dexamethasone is advised to be orally administered at a dose of 6 mg (or equivalent of other hormones) on the first day of the chemotherapy cycle and 3.75 mg (or equivalent of other hormones) on the second, third and fourth days. If the patient still develops nausea and vomiting after the use of these prophylactic measures, olanzapine quadruple therapy can be additionally used. Olanzapine is advised to be orally administered at a dose of 10 mg on the first day of the chemotherapy cycle and 10 mg on the second, third and fourth days of chemotherapy.

6.3.4 Hematopoietic Growth Factors

Granulocyte colony-stimulating factor (G-CSF) is not allowed as a primary prophylactic measure. During the second or subsequent chemotherapy cycles, the previous cycle is evaluated. If the subject has experienced febrile neutropenia or dose-limiting neutropenia events during the previous chemotherapy cycle, prophylactic use of G-CSF may be considered. It is also possible to reduce the risk of recurrence by delaying the administration or reducing the dose, which will be determined by the investigator based on the clinical reality.

7 Study Steps

7.1 Screening

The screening period will start from the signing of informed consent form and end at the time of randomization or screening failure. Subjects must proceed with any screening procedure specified by the study after the signing of informed consent form. The relevant data of laboratory tests and imaging assessments performed due to the needs of clinical routine care prior to the signing of informed consent, if within the specified window period, can be used.

Unless otherwise specified, the following screening should be completed within 28 days prior to the start of the study drug treatment:

- Signing of informed consent form;
- Collection of demographic data: gender, date of birth, ethnicity, height, weight, and body mass index;
- Tumor diagnosis: date of the first pathological diagnosis, pathological staging, metastatic site, clinical staging (locally advanced or distant metastasis);
- History of tumor treatment:
    - History of tumor surgery: name of surgery, date of surgery, date of recurrence after surgery;
    - History of radiotherapy: radiotherapy site, dose, start date and end date;
    - History of oncology drug treatment: regimen, cycle, start date and end date;
- History of accompanying diseases, history of previous medication (for antibiotics, the use within 3 months prior to randomization should be recorded), and history of drug allergy;
- Virological examination (completed within 14 days prior to the first dose): HBsAg (if positive, HBV-DNA is required), HBsAb, HBeAg, HBeAb, HBcAb, HCV-Ab (if positive, HCV-RNA is required) , HIV-Ab;
- EBV-DNA testing;
- Imaging examination: CT or MRI (both enhanced, except for cases where the contrast agent is contraindicated and a plain scan can be performed instead) of the nasopharynx, neck, chest, and abdomen (including pelvis). Brain MRI is required when the subject is suspected and diagnosed with brain metastasis (in cases where MRI is contraindicated, CT can be performed instead, both enhanced, except for cases where the contrast agent is contraindicated and a plain scan can be performed instead). The imaging examination results obtained before the signing of informed consent, as long as they meet the RECIST 1.1 requirements, can be used for baseline tumor assessment. The window for baseline imaging examination is extended to within 4 weeks prior to randomization. Bone scan is required when the subject is suspected or confirmed to have bone metastasis, and the time of examination is extended to within 42 days prior to randomization;
- AE: AE should be recorded since the signing of informed consent form;
- Concomitant medication: concomitant medications within 30 days prior to the signing of informed consent should be recorded in detail.

The following screening should be completed within 7 days before the start of the study drug treatment. Pregnancy test should be completed within 72 hours before the start of study medication.

- Check of inclusion and exclusion criteria;
- ECOG score;
- Vital signs: pulse, respiratory rate, body temperature, blood pressure;
- Comprehensive physical examination: general condition, head and face, neck, skin, lymph nodes, eyes, otolaryngology, oral cavity, respiratory system, cardiovascular system, abdomen, reproductive-urinary system, musculoskeletal system, nervous system, mental state, others;
- Blood routine: red blood cell count, hemoglobin, platelet count, white blood cell count, neutrophil count, lymphocyte count;
- Urine routine: white blood cells, red blood cells, urine protein. If the urine protein is ≥ 2+, it is necessary to additionally perform the 24-hour urine protein quantification;
- Fecal occult blood;
- Blood biochemistry: ALT, AST, GGT, total bilirubin, direct bilirubin, AKP, blood urea nitrogen or urea (preferably blood urea nitrogen), total protein, albumin, creatinine, blood glucose, lactate dehydrogenase, K^+, Na^+, Ca^{2+}, Mg^{2+}, Cl^-;
- Thyroid function: TSH, FT3, FT4;
- Coagulation function: APTT, PT, FIB, INR;
- Echocardiography: including LVEF assessment. Perform the examination as clinically suggested;
- 12-lead ECG: heart rate, PR interval, QT interval, QTcF. If there is an abnormality, additionally perform other necessary examinations according to the investigator's judgment;
- Pregnancy test (applicable to women of childbearing age);
- Randomization ;
- AE: record AEs in detail;
- Concomitant medication: record the concomitant medication in detail.

7.2 Trial Period

The trial period starts from the subject’s randomization, and the first administration should be completed within 3 days after randomization.

- All examinations and assessments (except for imaging examinations) should be completed within 3 days prior to D1 and D8 administration of each cycle. The following assessments should be completed prior to D1 administration of each cycle. If a test is completed in the screening period within 7 days prior to the first administration, it is not required to repeat the test before the first administration.
    - ECOG score;
    - Weight;
    - Vital signs: pulse, respiratory rate, body temperature, blood pressure;
    - Targeted physical examination: perform a targeted physical examination when clinically indicated;

  - Blood routine: red blood cell count, hemoglobin, platelet count, white blood cell count, neutrophil count, lymphocyte count;
  - Blood biochemistry: ALT, AST, GGT, total bilirubin, direct bilirubin, AKP, blood urea nitrogen or urea (preferably blood urea nitrogen), total protein, albumin, creatinine, blood glucose, lactate dehydrogenase, K^+, Na^+, Ca^{2+}, Mg^{2+}, Cl^-;
  - ECG: heart rate, PR interval, QT interval, QTcF;
  - AE: record AEs in detail;
  - Concomitant medication: record the concomitant medication in detail.
- The following examinations should be completed prior to the administration of gemcitabine on Day 8 of each cycle:
  - Blood routine: including red blood cell count, hemoglobin, platelet count, white blood cell count, neutrophil count, lymphocyte count;
- The following related tests should be completed prior to administration every 2 cycles. If a test is completed in the screening period within 7 days prior to the first administration, it is not required to repeat the test before the first administration.
  - Urine routine: white blood cells, red blood cells, urine protein. If the urine protein is ≥ 2+, it is necessary to additionally perform the 24-hour urine protein quantification;
  - Fecal occult blood (either before or after administration);
  - Thyroid function: TSH, FT3, FT4.
- The following related tests must be completed before administration every 3 cycles:
  - EBV-DNA testing.
- Imaging examination: baseline imaging examination is repeated every 6 weeks, and every 12 weeks since 16 months after the first dose. Unplanned imaging examination can be performed if progressive disease is suspected. For bone metastasis lesions, bone scans are only required when other lesions are evaluated as CR, and it is necessary to confirm whether the bone metastasis lesions have all disappeared, or when there are clinical indications. Imaging examinations allow a window period of ±7 days, and the imaging examination conditions should be the same as those at baseline (including scan layer thickness, contrast agent, etc.). The imaging examination time shall not be adjusted due to the delay in the start of the dosing cycle.

  Subjects with PD for the first time who are yet clinically stable should be confirmed after an interval of at least 4 weeks. If the time interval from the next planned imaging examination is less than 4 weeks, the originally planned imaging examination will be skipped once, and resumed at the predetermined time for the next imaging examination.
- ADA blood sampling: C1D1, C2D1, C4D1, C6D1, C9D1, once before each administration of camrelizumab/placebo, and once before administration every 4 cycles thereafter.

7.3 Withdrawal from Study Treatment Visit

This visit should be completed when the subject is confirmed to need to terminate the study treatment. If the following assessments and examinations are not performed within 7 days prior to the withdrawal from study treatment visit, they should be completed at the withdrawal from study treatment visit.

- ECOG score;
- Vital signs: pulse, respiratory rate, body temperature, blood pressure;
- Comprehensive physical examination: general condition, head and face, neck, skin, lymph nodes, eyes, otolaryngology, oral cavity, respiratory system, cardiovascular system, abdomen, reproductive-urinary system, musculoskeletal system, nervous system, mental state, others;
- Blood routine: red blood cell count, hemoglobin, platelet count, white blood cell count, neutrophil count, lymphocyte count;
- Urine routine: white blood cells, red blood cells, urine protein. If the urine protein is ≥ 2+, it is necessary to additionally perform the 24-hour urine protein quantification;
- Fecal occult blood;
- Blood biochemistry: ALT, AST, GGT, total bilirubin, direct bilirubin, AKP, blood urea nitrogen or urea (preferably blood urea nitrogen), total protein, albumin, creatinine, blood glucose, lactate dehydrogenase, K^+, Na^+, Ca^{2+}, Mg^{2+}, Cl^-;
- Thyroid function: TSH, FT3, FT4;
- ECG: heart rate, PR interval, QT interval, QTcF;
- Pregnancy test;
- EBV-DNA testing;
- Imaging examination: if imaging examination is not performed within 4 weeks prior to the withdrawal from study treatment visit, an imaging examination should be performed at the withdrawal from study treatment visit.
- ADA blood sampling;
- AE: record AEs in detail;
- Concomitant medication: record the concomitant medication in detail.

7.4 Safety Follow-up

All subjects should visit the study site for follow-up at 30 days after the last treatment. If the withdrawal from study treatment visit is completed, this visit is changed to telephone follow-up, and telephone follow-up is required at 60 and 90 days after the last dose. Telephone follow-up needs to obtain safety information (including AE outcomes, new SAEs, AEs of special interest, concomitant medications, etc.).

- Blood routine: red blood cell count, hemoglobin, platelet count, white blood cell count, neutrophil count, lymphocyte count;
- Blood biochemistry: ALT, AST, GGT, total bilirubin, direct bilirubin, AKP, blood urea nitrogen or urea (preferably blood urea nitrogen), total protein, albumin, creatinine, blood glucose, lactate dehydrogenase, K^+, Na^+, Ca^{2+}, Mg^{2+}, Cl^-;
- Thyroid function: TSH, FT3, FT4;
- ADA blood sampling: blood is collected once each at 30 days (if the withdrawal from study treatment visit is completed, no blood is collected at this time point), 60 days and 90 days after the last dose (according to visit arrangement, if applicable).

- Adverse event (AE): record AEs in detail;
- Concomitant medication: record the concomitant medication in detail.

7.5 Unscheduled Visit

If the subject needs to undergo unscheduled visits due to AE in the course of the trial, the following items should be recorded:

- Record concomitant medication;
- Record AE;
- Record the relevant examinations (including imaging examination, if any).

7.6 Survival Follow-up

Survival follow-up starts from the last administration, and is performed every 3 months through telephone follow-up or other effective ways. Whether the subject has subsequently received any new anti-tumor treatment should be recorded. If yes, record the treatment regimen and start and end time, and fill out the survival follow-up.

7.7 Tumor Progression Follow-up

Subjects removed for "non-PD" reasons (e.g., inability to tolerate AE, etc.) are advised to receive a tumor assessment at the frequency of efficacy evaluation (every 6 weeks ±7 days) stipulated for this study, and an imaging assessment every 12 weeks (±7 days) since 16 months after the first dose until observation of PD, initiation of a new anti-tumor treatment, withdrawal of informed consent, loss to follow-up or death. The follow-up information should be recorded in the eCRF.

8 Evaluation

8.1 Effectiveness Evaluation

Progression-free survival (PFS): the time from randomization to the date of the first documented objective tumor progression or death for any cause, whichever occurs first. The Independent Imaging Review Committee (IRC) will perform independent imaging assessment of the primary endpoint. See the Independent Imaging Review Committee Charter (IRC Charter) for details. The secondary endpoint is subject to the investigator's assessment. If the PFS is not available as of the cutoff date of analysis, the data will be censored. The censoring rules are detailed in the Statistical Analysis Plan (SAP).

PFS rates at 12 months, 18 months, and 24 months: the progression-free survival rates of subjects at 12 months, 18 months, and 24 months after enrollment.

Overall survival (OS): The time from randomization to the death of the subject due to various reasons. As of the analysis cutoff date, if OS is not available, the data will be censored. Please refer to SAP for the censoring rules.

2-year OS rate: survival rate of subjects within 2 years after enrollment.

Objective response rate (ORR): defined as the proportion of subjects with the best overall response (BOR) of CR and PR in each treatment group who have used the medication at least once. BOR is defined as the best response indicator from the date of randomization to the date of objective documentation of progression or the date of subsequent anti-tumor treatment (whichever occurs first). For subjects without documented progression or subsequent anti-tumor treatment, their BOR will be determined based on all the response assessment results.

Duration of response (DoR): time from the first PR or CR to the first PD or death. If PD or death is not available as of the cutoff date of analysis, the data will be censored. The censoring rules are detailed in the SAP.

Duration of response (DoR): the time from the first PR or CR to the first PD or death. On the analysis deadline, if the PD or death is not obtained, the data will be censored. The censored rules are detailed in SAP.

Disease control rate (DCR): the proportion of subjects with CR, PR, and SD in each treatment group who have used the medication at least once. DCR is the the best response indicator from the date of randomization to the date of objective documentation of progression or the date of subsequent anti-tumor treatment (whichever occurs first). For subjects without documented progression or subsequent anti-tumor treatment, their DCR will be determined based on all the response assessment results.

The immunogenicity analysis is the proportion of subjects with anti-camrelizumab antibodies.

The reference criteria for imaging assessments in this study are RECIST1.1 criteria. The requirements and frequency of imaging examinations are detailed in the trial flowchart.

8.2 Safety Evaluation

8.2.1 Pregnancy Test

Female subjects with childbearing potential have a serum or urine pregnancy test 72 hours before the first dose. Subjects with a negative test result should take appropriate contraceptive measures. If the test proves positive, the subject will fail the screening. Already enrolled subjects should have a serum or urine pregnancy test again at the withdrawal from study treatment visit.

8.2.2 Adverse Events

The severity of AE refers to NCI-CTC AE V4.03.

8.2.3 Laboratory Safety Evaluation

See the trial flowchart for details.

8.2.4 Vital Signs and Physical Examination

See the trial flowchart for details.

8.2.5 12-lead ECG

See the trial flowchart for details.

8.3 Independent Data Monitoring Committee

This study will establish an Independent Data Monitoring Committee (IDMC) to assess the efficacy and safety of the study drug at the data review meeting. An interim analysis will be performed when approximately 121 (60%) PFS events are collected, and the IDMC will make recommendations on whether to continue or terminate the study based on the efficacy and safety data results.

The committee will include 2 independent oncologists and 1 independent statistician. The IDMC review meeting will be held at the time specified in the IDMC Charter.

The results of the interim analysis will be provided to the IDMC by the non-blind independent statistician. The IDMC, as the main reviewer of the primary efficacy analysis and safety results, will advise the sponsor whether or not to terminate the study. If the IDMC recommends terminating the study, the efficacy and safety results may be unblinded to the sponsor, so that the sponsor can take actions for the recommendation. A non-blind independent statistician will record the degree of unblinding of the results of the interim analysis to relevant personnel. See the IDMC Charter for details.

After the data is reviewed, the IDMC will provide Jiangsu Hengrui with recommendations on whether to continue the study, whether to modify the protocol or whether to suspend the study, and ultimately, Jiangsu Hengrui Medicine Co., Ltd. will decide whether to adopt the IDMC's recommendations.

9 Reporting of Adverse Events

9.1 Adverse Event (AE)

The collection of AEs in this study starts from the signing of informed consent form until Day 90 after the last dose of camrelizumab/placebo or Day 30 after the last dose of gemcitabine or cisplatin (whichever occurs later). If the patient starts a new anti-tumor treatment during the AE collection period, after the new anti-tumor treatment starts, only study drug-related AEs are collected.

9.1.1 Definition of Adverse Event

AE refers to adverse medical events that occur after a clinical trial subject receives a drug, but does not necessarily have a causal relationship with the treatment. AE can be any adverse adverse undesirable symptoms, signs, laboratory test abnormalities or diseases, at least including the following circumstances:

1) Aggravation of the original (before entering the clinical trial) medical conditions/diseases (including aggravation of symptoms, signs, and laboratory abnormalities);
2) Any new AE: any new adverse medical conditions (including symptoms, signs, newly diagnosed diseases);
3) Abnormal laboratory test values or results with clinical significance.

The researchers should record in detail any AE that occurs in the subject, including the AE term and description of all related symptoms, time of occurrence, severity, relevance to the test drug, duration, measures taken against the study medication, and final result and outcome.

9.1.2 Criteria for Judgment of AE Severity

Refer to the grading criteria for adverse drug events in NCI-CTC AE V4.03. In case of AEs not listed in the table of NCI-CTC AE V4.03, please refer to the following criteria:

Grade	Clinical description of severity
1	Mild; without clinical symptoms or with mild clinical symptoms; only clinical or laboratory test abnormalities; no treatment indicated
2	Moderate; minimal, local or noninvasive treatment indicated; limited age-appropriate instrumental Activities of Daily Living (ADL). Instrumental ADL refers to cooking, shopping, using the phone, counting money, etc.
3	Severe or of important medical significance, but not immediately life-threatening; leading to hospitalization or prolonged hospitalization; leading to disability; limited self-care ADL. Self-care ADL refers to bathing, dressing, taking off clothes, eating, using the toilet, taking medicine, etc., not bedridden.
4	Life-threatening, emergent treatment indicated
5	Deaths related to adverse events

9.1.3 Judgment of Relationship between AE and Test Drug

During the AE collection period, all AEs, no matter whether they are related to the test drug or not, no matter whether the subject is assigned to the test drug group, and even despite the use of drugs or not, should all be collected and recorded. In the course of treatment, any discomfort reactions complained of by the subject or abnormal changes in objective laboratory test indicators should be honestly recorded, and the severity, duration, treatment measures and outcome of AE manifestations should be specified. The investigator should comprehensively judge the relationship between AE and the test drug by considering the following: for example, whether the occurrence of AE has a reasonable temporal sequence with medication, characteristics of the study drug, toxicological and pharmacological action of the study drug, whether the subject has concomitantly used other drugs, the subject's underlying disease, past medical history, family history, and challenge and re-challenge reactions, and evaluate the possible correlations between AE and investigational medication by five-grade classification—"certainly related, possibly related, possibly unrelated, certainly unrelated and cannot be determined".

9.2 Serious Adverse Event (SAE)

9.2.1 Definition of SAE

SAE refers to medical events that occur in the course of the clinical trial and require hospitalization or prolonged hospitalization, lead to disability, affect working capacity, threaten life or cause death, or lead to congenital deformity, including the following medical events:

- Events leading to death;
- Life-threatening events (defined as the risk of immediate death of the subject at the time of the event);
- Events requiring hospitalization or prolonged hospitalization;
- Events leading to permanent or severe disability/dysfunction/affect working capacity;
- Congenital deformity or birth defect;
- Other important medical events (defined as events that are harmful to the subject or require interventions to prevent any of the serious consequences mentioned above).

9.2.2 Hospitalization

AE in the clinical study that lead to hospitalization (even for less than 24 hours) or prolonged hospitalization should be considered as SAE.

The following hospitalizations are excluded:

- Rehabilitation institution;
- Nursing homes;
- Regular emergency room admission;
- Day surgery (outpatient/current/non-bed surgery);
- Social reasons (for reimbursement by medical insurance).

Hospitalization or prolonged hospitalization unrelated to the worsening of AE is not SAE. For example:

- Hospitalization due to the original disease. There is no new adverse event or aggravation of the original disease (e.g., for testing of laboratory test abnormalities that have persisted from before the trial up to now);
- Hospitalization for administrative reasons (e.g., annual routine physical examination);
- Hospitalization as specified in the trial protocol during the clinical trial period (e.g., operations according to the requirements of the trial protocol);

- Elective hospitalization unrelated to the worsening of AE (e.g., elective surgery);
- Prescheduled treatment or surgery should be documented in the entire trial protocol and/or in the subject's personal baseline data;
- Admission to the hospital only for the use of blood products.

Diagnostic or therapeutic invasive (e.g., surgery) or non-invasive operations should not be reported as an AE, but when the disease condition that results in such operations is in line with the definition of AE, it should be reported. For example, acute appendicitis that occurs during the AE reporting period should be reported as an AE, while the appendectomy thus performed should be recorded as the treatment of the AE.

9.2.3 Progressive Disease and Death

PD is defined as deterioration of the subject's condition caused by the indication under study, including imaging progression and progression of clinical symptoms and signs. New metastatic lesion relative to the primary tumor and progression of the original metastatic lesions are both regarded as PD. Life-threatening events, events requiring hospitalization or prolonged hospitalization, or events leading to permanent or severe disability/dysfunction/affect working capacity, and congenital anomalies or birth defects caused by the symptoms and signs of PD are not reported as SAE. Deaths caused by the symptoms and signs of PD are reported as SAE.

In clinical trials, if a subject dies during the safety follow-up period, it must be reported as an SAE, regardless of whether the investigator assesses that it is possibly related to disease progression, or whether the subject has received other anti-tumor treatments. The word "death" should not be used as an SAE term, but as the result of an event, and the event leading to death should be recorded as an SAE. The medical condition/disease (including deterioration of symptoms and signs) that has caused or led to death is recorded in eCRF as an SAE term and reported as an SAE. If the cause of death cannot be determined at the time of reporting, the SAE term is recorded as "death of unknown cause".

If the investigator assesses that the subject dies due to disease progression, the grade 5 event caused by disease progression should be recorded in the eCRF and should be reported as an SAE; if the death caused by disease progression cannot be attributed to a specific medical event, then grade 5 "tumor progression" should be recorded in the eCRF and reported as an SAE, and the investigator should provide evidence of death caused by disease progression (for example, imaging changes suggesting tumor development or progression, and clinical deterioration related to the disease process).

9.2.4 Abnormal Liver Enzymes

If abnormalities in AST and/or ALT levels are observed for two consecutive times with abnormally elevated total bilirubin levels, the following conditions are met and there is no other cause of the abnormality, the situation should be regarded as an important medical event and reported as an SAE.

**Baseline period**	Normal (AST/ALT and total bilirubin)	Abnormal (any of AST/ALT and total bilirubin)
**Treatment period**	• ALT or AST ≥ 3× ULN<br>• with total bilirubin ≥ 2× ULN<br>• and alkaline phosphatase ≤ 2× ULN<br>• and no hemolysis	• ALT or AST ≥ 2 × baseline level, and value ≥ 3 × ULN; or ALT or AST ≥ 8 × ULN<br>• with total bilirubin increased by ≥ 1× ULN or value ≥ 3× ULN

Subjects who have abnormalities in AST and/or ALT levels during the treatment period or follow-up period with abnormally elevated total bilirubin levels should return to the study site as soon as possible (preferably within 48 hours) for evaluation after knowing the abnormal results. The evaluation should include laboratory tests, detailed inquiry of medical history and physical evaluation, and the possibility of liver tumors (primary or secondary) should be considered.

In addition to repeated testing of AST and ALT, the required laboratory tests should also include albumin, creatine kinase, total bilirubin, direct and indirect bilirubin, γ-glutamyl transferase, prothrombin time (PT)/international normalized ratio (INR) and alkaline phosphatase. Detailed medical history collection should include history of drinking, acetaminophen, soft drugs, various supplements, family history, occupational exposure, sexual history, travel history, history of contact with patients with jaundice, and history of surgery, blood transfusion, liver disease or allergic diseases. Further examinations may also include the detection of acute hepatitis A, B, C and D and liver imaging examination (e.g., biliary tract). If repeated testing still confirms consistency with the definition of the above laboratory criteria, in the absence of other causes leading to abnormal liver enzymes, the case should be reported as an SAE.

9.2.5 SAE Reporting System

The term of SAE collection should start from the subject's signing of the informed consent form until the end of the safety follow-up period or initiation of a new anti-tumor treatment (whichever occurs first). In the event of an SAE, regardless of initial report or follow-up report, the investigator must immediately fill out the *Serious Adverse Event Report Form*, sign and date the form, notify the sponsor within 24 hours after knowing it, and timely report to the relevant organization according to the regulatory requirements.

SAEs that occur after the safety follow-up period and are suspected to be related to the study drug should be collected. For SAEs, the symptoms, severity, relevance to the test drug, time of occurrence, treatment time, measures taken, follow-up time and manner, and outcome should be recorded in detail. If the investigator thinks that an SAE is unrelated to the test drug but potentially related to the study conditions (e.g., termination of the original treatment, or complications during the trial), the relationship should be described in detail in the narrative section of the SAE Report Form. If the intensity of a currently occurring SAE or its relationship with the test drug has changed, a follow-up report should be submitted immediately. If the investigator believes that the previously reported SAE has misreported information, it can be corrected, revoked or downgraded in the follow-up report, and reported in accordance with the SAE reporting procedures.

The sponsor's email to receive SAE reports in this project is: hengrui_drug_safety@hrglobe.cn

9.2.6 AE/SAE Follow-up

All AE/SAEs should be followed up until the events are cured, relieved to baseline level or grade ≤1, reach a stable state, or can be reasonably explained (e.g., loss to follow-up, death) or they are finally confirmed to be unrelated to the investigational medicinal product or research process at the end of the safety follow-up period. Every effort should be made to ensure that subjects get the best outcome and a clear causality assessment.

The investigator should, at each visit, inquire about AE/SAEs that have occurred since last visit, and require subjects to provide follow-up information according to the sponsor's query requirements. At the end of the study, reference can be made to the following principles for the collection and follow-up periods of AE/SAE occurring after the subjects' last administration.

**Classification**	**Collection/recording requirement**	**Follow-up requirement**
Non-drug-related AE	Until the end of the safety follow-up period or initiation of a new anti-tumor treatment (whichever occurs first)	Until the end of the safety follow-up period
Drug-related AE	Until the end of the safety follow-up period	Follow-up until it is cured, relieved to baseline level or grade ≤1, reach a stable state, or can be reasonably explained (e.g., loss to follow-up, death).
Non-drug-related SAE and SIE	Until the end of the safety follow-up period or initiation of a new anti-tumor treatment (whichever occurs first)	Until the end of the safety follow-up period
Drug-related SAE and SIE	No time limit	Follow-up until it is cured, relieved to baseline level or grade ≤1, reach a stable state, or can be reasonably explained (e.g., loss to follow-up, death).

AE: adverse event; SAE: serious adverse event; SIE: adverse event of special interest.
Note: Regardless of whether the subject receives other anti-tumor treatments, all deaths during the safety follow-up period must be reported as SAEs.

9.3 Pregnancy

During the clinical trial period, female subjects who are pregnant will immediately discontinue the study drug treatment. If a male subject's partner is pregnant during the clinical trial period, the subject continues the clinical trial. The investigator should fill out the *Pregnancy Report/Follow-up Form for Hengrui Clinical Trials* and report to the sponsor within 24 hours after knowing the pregnancy event.
The investigator should follow up the pregnancy outcome until 1 month after the mother's delivery, and report the result to the sponsor.
If the pregnancy outcome is stillbirth, spontaneous abortion or fetal malformation, it will be considered as an SAE, and should be reported according to the time limit requirements for SAE.
If the subject also develops an SAE during pregnancy, then the investigator should also fill out the *Serious Adverse Event Report Form*, and must also comply with the SAE reporting procedures for reporting.

9.4 AE of Special Interest

In the event of AEs of special interest listed below, the investigator should fill out the *Adverse Events of Special Interest Report Form for Hengrui Clinical Trials* within 24 hours after knowing it, and report to the sponsor. If the event is also an SAE, the investigator should also fill out the *Serious Adverse Event Report Form* and report to the relevant organization according to SAE procedures.

- Grade ≥3 infusion reactions
- Grade ≥ 3 immune-related AE.

10 Clinical Monitoring

The monitor must follow the GCP and standard operating procedure (SOP), visit the study site regularly or according to the actual situation to conduct clinical monitoring work, supervise the implementation and progress of the clinical trial work, check and confirm the correct and complete input of all data records and reports and Case Report Forms, as well as their consistency with the source data, and ensure that the clinical trial is conducted in accordance with the clinical trial protocol. The investigator should actively cooperate with the monitor's work. The monitors' specific jobs include:

- Prior to the trial, confirm that the trial undertaker has had the appropriate conditions, including staffing and training, laboratories are fully equipped and working well and have a variety of trial-related test conditions, it is estimated that a sufficient number of subjects will be involved, and the participating researchers are familiar with the requirements of the trial protocol;
- Monitor the investigators' implementation status of the trial protocol during the trial, confirm that the informed consent form has been obtained from all subjects before the trial, know the enrollment rate of subjects and the progress of the trial, and confirm that the enrolled subjects are qualified;
- Confirm that all data records and reports are correct and complete, and that all case reports are entered correctly and consistent with the original data. All errors or omissions have been corrected or specified, and signed and dated by the investigator. Each subject's dose changes, treatment changes, concomitant medication, intermittent disease, loss to follow-up, and examination omissions should be confirmed and recorded. Verify that the withdrawal and loss to follow-up of enrolled subjects have been described in the Case Report Form;
- Confirm that all AEs are documented, and SAEs are reported and documented within the specified time; verify that the investigational medicinal products are supplied, stored, distributed and recovered in accordance with relevant regulations, and the corresponding records are made;
- The monitor should clearly and faithfully record the visits that the investigator failed to do,

the tests that have not been conducted, the checks that have not been performed, and whether mistakes and omissions have been corrected;

- After each visit, a written monitoring report shall be completed. The report shall include the date and time of the monitoring, the name of the monitor and the findings of the monitoring.

The sponsor's quality assurance department may audit the trial in the clinical study institution. The audit includes drug supply, required trial documentation, records of the informed consent process, and consistency of the Case Report Forms with the original documents. The content and scope of the audit can also be increased. The investigator agrees to participate with reasonable time in a reasonable manner.

11 Data Analysis/Statistical Method

The detailed statistical analysis for this study will be included in the Statistical Analysis Plan (SAP) which will be preserved by the sponsor. For the plan specified in the protocol, appropriate changes can be made in the SAP. However, any important revision to the definition and analysis of the primary study endpoint shall be reflected in the protocoal amendment.

11.1 Sample Size

The study is a parallel controlled study. The primary study endpoint is IRC-assessed PFS. The parameters for calculation of sample size are as follows:

1. Enrollment duration=12 months, follow-up duration=24 months (the overall duration is 36 months)
2. 1:1 randomized
3. α=0.025 (one-sided)
4. Hazard ratio (HR, test group/control group)=0.63 (the median PFS in the control group is 7 months)

An interim analysis is planned for PFS. The α spending function of the Hwang-Shih-DeCani (gamma=-4) approximate O'Brien& Fleming boundary is used to allocate α. The interim analysis of PFS will be performed when 121 (60%) events are collected. The final analysis of PFS will be performed when 201 events are collected. Under the premise of ensuring that the overall type I error does not exceed one-sided 0.025, a power of about 90% can be obtained(Using East 6.5 simulation results). Given a dropout rate of 10% in the course of study, eventually 250 subjects need to be enrolled (125 cases in each group).

11.2 Analysis Data Sets

- Full Analysis Set (FAS): according to the ITT principle, all subjects who have used the study drug at least once after randomization. FAS is the primary analysis set for efficacy analysis in this study.
- Per-Protocol Set (PPS): a sub-set of subjects defined in FAS. Subjects with major protocol deviations that have important impacts on the results are excluded from this set.
- Safety Analysis Set (SS): subjects who have used the study drug at least once after randomization.

Before database locking, the principal investigator, the statistician and the sponsor will jointly negotiate to determine the final analysis data sets at the data review meeting.

11.3 Statistical Analysis

11.3.1 Basic Method

The study is a parallel controlled design. Unless otherwise specified, the appropriate statistics will be used for all data by randomization group and according to the data type: continuous variables are descriptively statistically summarized using mean, standard deviation, median, minimum and maximum, and categorical variablesusing frequency and percentage. For time-dependent data, the Kaplan-Meier method will be used, and if necessary, the survival curve will be plotted; the above analyses, if necessary, will provide the corresponding 95% confidence interval.

11.3.2 Primary Effectiveness Endpoint Analysis

The primary endpoint of this study is the IRC-assessed PFS. The primary analysis will be based on the FAS, and a stratified Log-Rank test that considers and does not consider random stratification factors to compare the survival function of PFS in the two groups. In addition, the Kaplan-Meier method is used to estimate the survival rate of PFS, plot the survival curve and estimate the median PFS and its 95% confidence interval (Brookmeyer-Crowley method).The analysis considering stratification factors is the primary analysis.

In addition, as a supportive analysis, the Cox model considering and not considering stratification factors is used to estimate the hazard ratio between the two groups and calculate its 95% confidence interval (Wald method) on the premise that the proportional hazard assumption is established.

The specific analysis method for the analysis of the PP set is the same as the analysis method for the FAS.

11.3.3 Secondary Effectiveness Endpoint Analysis

The investigator-assessed PFS and OS are statistically analyzed and described using a method similar to what is used for the primary indicators.

For objective response rate (ORR) and disease control rate (DCR), estimate the rates in both groups and their two-sided 95%CI (Clopper-Pearson method), and calculate the difference in rate between the two groups and its two-sided 95%CI (normal approximation), as well as P value for comparison between groups (Cochran-Mantel-Haenszel method). DoR is statistically described.

Other indicators will be statistically described and summarized according to general principles.

The analysis of secondary effectiveness endpoints will be based on the FAS.

11.3.4 Processing of Missing Data

In this trial, missing data of efficacy indicators will not be specifically processed. The censoring rules are detailed in the SAP. Missing values will not be estimated in the safety evaluation.

11.3.5 Safety Analysis

AEs that occur during the trial will be encoded using the MedDRA dictionary. The frequency and incidence of AEs will be described in terms of system organ class and preferred term. The relevance and severity of AEs will be further described in a tabular form. Other safety indicators will be summarized with descriptive statistics. The incidences of AEs, adverse reactions, AEs leading to withdrawal from study treatment, AEs leading to death and SAE are summarized. Severity of AEs and adverse reactions: if the same subject has experienced the same AE for many times, the most severe one will be included in the analysis; if the same subject has experienced different AEs, the most serious AE will be included in the analysis.

Laboratory test indicators: the descriptive summary of laboratory test values is primarily targeted at outliers.

Vital signs: the mean, maximum, minimum, median, and standard deviation are used to describe the measurements and changes at each visit.

Descriptive analysis is performed on physical examination and 12-lead ECG.

The baseline is defined as the most recent test data from the first dose.

11.3.6 Interim Analysis

In this study, the stratified log-rank method will be used to perform two tests on the original hypothesis of the same distribution of progression-free survival time between the two groups of subjects (interim analysis and final analysis). An interim analysis will be performed when 60% of PFS events are collected in the study. According to the plan, the termination criteria at the time of the interim analysis and final analysis of PFS are as follows:

Analysis	Value	α=0.025
Interim analysis: accounting for about 60%	Superiority boundary Z	-2.596
Number of events: 121	Nominal significance level (one-sided)	0.0047
Time: Approximately 15.3 months	Superiority boundary HR	0.627
Final analysis	Superiority boundary Z	-1.989
Number of events: 201	Nominal significance level (one-sided)	0.0234
Time: Approximately 32.5 months	Superiority boundary HR	0.758

Note: The actual nominal significance level and boundary during the interim analysis will be determined according to the actual proportion of events.
The above calculation will be obtained in EAST 6.5 using the α spending function of HSD (gamma=-4) approximate O'Brien-Fleming boundary.

The interim analysis (IA) of PFS will be performed by the Independent Data Monitoring Committee (IDMC). If the efficacy is confirmed during the interim analysis of PFS, the committee may recommend stopping the study. Otherwise, the trial will continue until the final analysis of PFS.

11.3.7 Subgroup Analysis

Subgroup analysis of the primary indicator PFS will be performed according to the following factors (at least but not limited to) and an HR forest graph.

Stratification factors:

Liver metastasis (yes vs. no);

Whether the subject has received radical concurrent radiochemotherapy (yes vs. no)

ECOG score (1 vs. 0).

11.3.8 Exploratory Analysis

Descriptive statistics will be used to analyze the following exploratory study endpoint:

- To test of the proportion of anti-camrelizumab antibodies.

12 Data Management Method

12.1 Data Recording

The study will use the electronic Case Report Form (eCRF) to collect and manage research data.

12.1.1 eCRF Filling

Clinical research data will be collected using HRTAU EDC.

Filling: The data in the eCRF are derived from the original documents such as original medical records and laboratory test reports, and should be consistent with the original documents. Any observation and test results in the trial should be timely, correctly, completely, clearly, standardizedly and truthfully filled in the eCRF, and may not be changed arbitrarily.

Modification: If necessary, if data correction is made to the eCRF, the reason for data modification should be filled out as prompted by the system. The system logic check program will check the completeness and logic of the clinical trial data entered into the EDC system, and produces errorous information prompts for problematic data. PI or data entry personnel (CRC) is/are allowed to revise or explain problematic data, and when necessary, queries can be sent repeatedly until the problemtic data are solved.

12.1.2 eCRF Review

The investigator and his designee should complete the eCRF filling, review and submission in a timely manner. The PI or CRC should respond promptly to queries from monitors, data administrators and medical auditors. After the completion of data clear-up, the investigator should sign on the completed eCRF for confirmation.

12.2 Data Monitoring

Implementer: Monitor.

Monitoring content: whether the study protocol is followed; whether all CRF filling is correct and complete, and consistent with the study medical records, laboratory test reports and other original documents, and whether the data have errors or omissions. The monitor needs to review the completeness, consistency and accuracy of the trial data in the clinical database according to the monitoring plan, and discuss with researchers about problematic data. When necessary, researchers should make supplementation or correction to ensure that the data in the eCRF are consistent with the original data. This process is also known as Source Data Verification (SDV).

12.3 Data Management

12.3.1 Establishment of EDC Database

The data administrator establishes the study data acquisition system and database according to the study protocol and provides on-line use before enrollment of the first subject. Before use, all EDC users need to be adequately trained and acquire the corresponding account to log on the system.

12.3.2 Data Entry and Verification

The investigator or CRC should enter data into the EDC system according to the visit process requirements and eCRF filling guide. After the eCRF is submitted, the monitor, the data administrator, and the medical staff should review the data one by one, and require the investigator or CRC to answer questions about problems found in review in the form of query. After the completion of clean-up, the investigator should sign on the eCRF for confirmation.

12.3.3 Data Review and Database Locking

After the clinical trial is completed, the study director, the sponsor, statistical experts and the data administrator will jointly perform data review before statistical analysis. The important content of such review is determination of the analysis data set that each case belongs to (including FAS, PPS and SS), judgment of missing values and handling of outliers. Decisions made under data review cannot be revised, and any decision shall be documented.

After the SDV of the CRA is completed, the data management personnel and medical staff will perform the final quality control of all data in the database, summarize all protocol deviations and protocol violation events that have occurred during the trial, and hold data verification meetings. Once the data in the database meets the quality requirements, the database will be locked, and the data management personnel will export the data for data analysis by the statistical department.

12.3.4 Data Archiving

Upon completion of the study, the EDC system needs to generate the subject eCRF in PDF format and store it on a non-rewritable light disk, and submit it to the sponsor and each institution respectively for preservation and archiving for future audits. The storage and management of the study data must be carried out in accordance with the GCP requirements. The investigator should notify the sponsor in advance when destroying any documents or records related to the study. After the test drug is approved for marketing, or even if the clinical study is terminated early, the sponsor should keep the clinical trial data for at least 15 years.

13 Source Data and Original Documents

According to ICH E6, relevant regulations and the study institution's requirements on the protection of personal information of the subject, each study site must properly keep the treatment and scientific research records related to this study. As part of Hengrui's funding or participation in the study, each study site should allow representatives authorized by Hengrui and regulatory agencies to inspect (to reproduce if permitted by law) its clinical records for quality review, and auditing, as well as safety, study progress and data validity evaluation. This section should also indicate who has access to these records.

Source data is all information required for the reconstruction and evaluation of clinical studies, and is the original record of clinical findings, observations or other activities. Examples of such original documents and data records include, but are not limited to, hospital records, laboratory records, memos, subject diary card, pharmacy dispensing records, sound records of counseling sessions, record data of automated instruments, copied or transcribed records that are verified as accurate and complete, microfiche, photographic plate, microfilm strip or disk, X-ray films and documents and records of subjects preserved in the pharmacies, laboratories and medico-technical departments participating in the study.

14 Quality Assurance and Quality Control

In order to ensure the quality of the trial, before the official start of the trial, the sponsor and the investigator will jointly discuss and develop a clinical study plan. GCP training is offered to all relevant research personnel who participate in the trial.

Each study site must follow the SOP for management of study medications, including receiving, preservation, distribution, recovery and destruction (if applicable).

In accordance with the GCP guidelines, necessary steps should be taken in the design and implementation phases of the study to ensure that the data collected are accurate, consistent,

complete and credible. All the observed results and abnormal findings in the clinical trial should be verified and recorded promptly to ensure data reliability. The instruments, equipment, reagents and standards used by various examinations in the clinical trial should have strict quality standards, and it should be ensured that they are working in a normal state.
The investigator will enter the information required by the protocol into the eCRF. The monitor will verify whether the filling is complete and accurate, and guide the staff of the study site to make necessary amendments and additions.

Drug supervision and administration departments, Institutional Review Board (IRB)/Independent Ethics Committee (IEC), and monitors and/or auditors of the sponsor may conduct systematic reviews of clinical trial-related activities and documents to assess whether the trial is conducted in accordance with the requirements of the trial protocol, SOP and the relevant regulations (e.g., Good Laboratory Practices [GLP] and Good Manufacturing Practices (GMP)), and whether the test data are timely, truly, accurately and completely recorded. The audits should be performed by persons who are not directly involved in the clinical trial.

15 Regulatory Ethics, Informed Consent and Subject Protection

15.1 Regulatory Considerations

As required by the relevant domestic regulations, before a new drug clinical trial is carried out, an application should be submitted to the CNDA to obtain drug clinical trial approval in order to carry out the clinical trial. The clinical approval number for the preparations of camrelizumab is 2016L01455.
The regulatory basis of the protocol design is:

- Drug Registration Regulation;
- Good Clinical Practice;
- Based on consensus on ethical principles in international ethical guidelines, including the Declaration of Helsinki and the international ethical guide of the Council for International Organizations of Medical Sciences (CIOMS);
- Other applicable laws and regulations.

15.2 Ethical Norms

The trial protocol shall be reviewed by the hospital’s Ethics Committee and obtain written approval prior to its implementation. The Ethics Committee should be provided with the study protocol, protocol amendments, informed consent form and other related documents, such as recruitment advertisement and other documents. The clinical trial must follow the Declaration of Helsinki, GCP promulgated by the CNDA and relevant regulations. Prior to initiation of the trial, the study must be approved by the hospital’s Ethics Committee before it is started.
Without the consent of both the sponsor and the investigator, neither party may unilaterally modify the trial protocol of this study. The investigator may make changes to or deviations from the study protocol prior to the approval of the Ethics Committee/Institutional Review Board only in order to eliminate direct and immediate harm to subjects. Meanwhile, the deviations or changes made and their causes, as well as the proposed amendments to the protocol should be submitted to the Ethics Committee/Institutional Review Committee for review as soon as possible. The investigator must explain and record any protocol deviation.
During the clinical study period, any modification made to the trial protocol should be submitted to the Ethics Committee and, if necessary, other study documents should be modified accordingly and submitted and/or approved as required by the Ethics Committee. The investigator has the responsibility to submit interim reports of the trial on a regular basis in accordance with the relevant requirements of the Ethics Committee. After the trial ends, the Ethics Committee should be informed that the trial has ended.

15.3 Independent Ethics Committee

The protocol, informed consent form, recruitment materials, and materials from all subjects will be submitted to the Ethics Committee for review and approval. Subjects can be enrolled only after the protocol and informed consent form have been approved. Any amendments to the protocol must be reviewed and approved by the Ethics Committee before they can be implemented. All amendments to the informed consent form must also be approved by the Ethics Committee, and the Ethics Committee will decide whether subjects who have signed the previous version of informed consent form needs to sign the new version again.

15.4 Informed Consent

15.4.1 Informed Consent Form and Other Written Information Required for Subjects

The informed consent form describes the study medication and study process in detail, and fully explains the study risks to subjects. Before a subject proceeds with any study-related process, the written document of the subject's informed consent must be obtained in advance. The following informed consent materials will be submitted together with the protocol:

Informed consent form;

Recruitment advertisement.

15.4.2 Informed Consent Process and Record

Informed consent starts before any individual agrees to participate in the clinical study and continues throughout the clinical study. The risks and possible benefits of participating in the study will be thoroughly and sufficiently discussed with the subjects or their legal agents. Subjects will be required to read and review the informed consent form that has been approved by the Ethics Committee. The investigator will explain the clinical study to subjects and answer any question that subjects may raise. Subjects can start participating in the study only after they sign the informed consent form. Throughout the clinical study, subjects may withdraw their consent at any time. A copy of the informed consent form will be kept by subjects. Even the inquired patients refuse to participate in the study, their benefits will be fully protected, and the quality of their medical care will not be affected in any way.

15.5 Confidentiality of Subject Information

The confidentiality of subject information is strictly enforced by the investigator, study participants, the sponsor and its agent. Confidentiality also covers biological samples and genetic testing in addition to the subject's clinical information. As a result, the study protocol, files, data, and all other information generated therefrom will be kept strictly confidential. No relevant research or data information shall be disclosed to any unauthorized third party without the prior written approval of the sponsor.

Other authorized representatives of the sponsor, IRB or regulatory authorities can inspect all documents and records that the investigator is required to maintain, including but not limited to medical records and medication records of subjects. The study site should allow access to these records.

The contact information of subjects will be securely stored at each study site and will only be used internally in the course of the study. At the end of the study, all records will continue to be kept in a safe place according to the time limit set by the local IRB and regulations.

The research data of subjects collected for statistical analysis and scientific reporting will be uploaded and stored at Sun Yat-sen University Cancer Center, which should not include subjects contact information or identification information. In contrast, individual subjects and their research data will all have a separate study identification number. The study data entry and study management systems used by the research personnel at each clinical study site and Sun Yat-sen University Cancer Center are confidential and password-protected. At the end of the study, the identification information in all research databases will be eliminated, and archived at Sun Yat-sen University Cancer Center.

15.5.1 Use of Samples, Specimens or Data for Research Purposes

- Scheduled use: samples and data collected according to the protocol will be used for exploratory studies to detect the proportion of anti-camrelizumab antibodies, and will not be used for any other irrelevant purposes.
  - Preservation: samples and data, when preserved, will be numbered by the study. Data in computers will also be password-protected. Only researchers have access to such samples and data.

15.5.2 Future Use of Preserved Specimens

The study will not preserve specimens for purposes other than those specified in the protocol.

16 Publication of Study Results

Study results are owned by Jiangsu Hengrui Medicine Co., Ltd. Hengrui does not restrict the publication of any information collected or obtained from the study by the investigator, no matter whether the results are in favor of the study drug or not. However, in order to prevent inadvertent disclosure of confidential information or unprotected inventions, the investigator should allow the sponsor to have the opportunity to review any proposed publication or other form of publicity before the document is submitted or published. The investigator should provide the original text, abstract or full text of all planned publications (posters, invited presentations or guest presentations) to Hengrui at least 30 days prior to the document submission or other form of publication. If a patent needs to be obtained in order to protect intellectual property rights, the investigator should agree to postpone the release, and the postponement should not exceed 60 days. Prior to publication, Hengrui may require the investigator to delete any previously unpublished confidential information (other than the results of the study). If the study is part of a multi-center study, the investigator should agree that the first publication is a composite result of all study sites. However, if the original text of a comprehensive analysis has not been submitted for publication within 12 months after the completion or termination of the study at all study sites, the investigator may independently publish the results according to other requirements of this section.

17 Key Functions and Roles

Principal investigator of the group leader organization	Zhang Li, Professor<br>Sun Yat-sen University Cancer Center<br>No. 651, East Dongfeng Road, Guangzhou<br>Tel:<br>Email:
Sponsor’s medical director	Yang Qing, MD, Clinical Medical Director<br>Jiangsu Hengrui Medicine Co., Ltd.<br>No. 7, Kunlunshan Road, Lianyungang Economic and Technological Development Zone, Jiangsu Province<br>Tel: 021-50118400<br>Email: yangqing@hrglobe.cn
Sponsor’s statistician	Zhang Ben<br>Jiangsu Hengrui Medicine Co., Ltd.<br>No. 7, Kunlunshan Road, Lianyungang Economic and Technological Development Zone, Jiangsu Province<br>Tel: 021-50118400<br>Email: zhangben@hrglobe.cn

18 Clinical Study Progress

Expected enrollment time of the first subject: November 2018

Expected enrollment time of the last subject: November 2019

Expected time of end of study: 2 years after the last enrolled subject’s first dose

19 References

[1] Wang Qing, et al., Chinese Journal of Cellular and Molecular Immunology, 2013, 29 (1): 109.

[2] Zibing Wang, et al, Oncoimmunology, 2017, 6(7): e1331807.

[3] Yang L, Hong S, Wang Y, et al., Scientific Report 2015; 15638

[4] Wei WI, Sham JS. Nasopharyngeal carcinoma. Lancet 2005; 365:2041-54

[5] Li Zhang, Yan Huang, Shaodong Hong, et al., Lancet 2016; 388; 1883-92

[6] Zibing Wang, et al, Oncoimmunology, 2017, 6(7): e1331807.

[7] Mario Roselli, et al, OncoImmunology, 2:10, e27025.

[8] W. Joost Lesterhuis, et al, The Journal of Clinical Investigation, 2011, 128(1):3100-3108.

[9] Tomokazu Tanaka, et al. Expert Opinion on Biological Therapy, 2017, 723-733

[10] C. Hsu, S. Lee, S. Ejadi, et al., Annals of Oncology 26 (Supplement 9): ix93–ix102,

2015.

[11] C. Langer, S.M. Gaddgeel, H. Borghaei, et al., ESMO 2016, LBA46.

[12] T.K. Tau, Anne W.M.Lee, Dominique H.M. Wong et al., Internaltional Journal of Radiation Oncology. 2006; 66, (4), 1004–10

[13] Yin Li, Ke Pan, Li-zhi Liu et al., PLOS One, 2015, 22; 10(6):e0130620

Annex I ECOG Performance Status Scoring Criteria

Grade	Performance status
0	Fully active, able to carry on all pre-disease performance without restriction;
1	Fully able to walk and able to carry out work of a light or sedentary nature, e.g., light house work, office work, but restricted in physically strenuous activity;
2	Able to walk, capable of all self-care but unable to carry out any work activities, spending <50% of daytime in bed;
3	Capable of only limited self-care, confined to bed or chair more than 50% of waking hours.
4	Totally confined to bed, cannot carry on any self-care.
5	Dead.

Annex II Calculation of Creatinine Clearance

Calculation of creatinine clearance with Cockroft-Gault formula

Serum creatinine concentration (mg/dL):

Male creatinine clearance rate (mL/min)= $\frac{(140-\text{age}) \times (\text{weight})\ \text{a}}{72 \times \text{serum creatinine}}$

Female creatinine clearance rate (mL/min)=$\frac{0.85\times(140-\text{age}) \times (\text{weight})\ \text{a}}{72 \times \text{serum creatinine}}$

Serum creatinine concentration (μmol/L):

Male creatinine clearance rate (mL/min)=$\frac{(140-\text{age}) \times (\text{weight})\ \text{a}}{0.818\times \text{serum creatinine}}$

Female creatinine clearance rate (mL/min)=$\frac{0.85\times(140-\text{age}) \times (\text{weight})\ \text{a}}{0.818\times \text{serum creatinine}}$

a Age unit is year, while weight unit is KG.

Annex III AJCC Cancer Staging System: 8th Edition (Excerpt) *

T staging

Tx: Primary tumor cannot be assessed

T0: No tumor found, but EBV positive and with cervical metastatic lymph nodes

T1: Tumor is confined to the nasopharynx, or has grown into the oropharynx and/or nasal cavity, without parapharyngeal space involvement

T2: Tumor has grown into the parapharyngeal space, and/or with adjacent soft tissue involvement (ptereri muscle, pterygoid muscle, anterior vertebral muscle)

T3: Tumor has grown into the skull base bone structure, cervical vertebrae and winged structure, and/or paranasal sinus

T4: Tumor has grown into the skull, with cranial nerves, hypopharyngeal, orbital and parotid gland involvement, and/or with extensive soft tissue invasion beyond the lateral margin of the pterygoid muscle

N staging

Nx: Regional lymph nodes cannot be assessed

N0: No regional lymph node metastasis

N1: Unilateral cervical and/or posterior pharyngeal lymph node metastasis (regardless of the number of sides): maximum diameter ≤ 6 cm, and located above the lower edge of the annular cartilage

N2: Bilateral cervical lymph node metastasis: maximum diameter ≤ 6 cm, located above the lower edge of the annular cartilage

N3: Cervical lymph node metastasis (regardless of the number of sides): maximum diameter >6 cm and (or) located below the lower edge of the annular cartilage

M staging

M0: Without distant metastasis

M1: With distant metastasis

Clinical staging

Stage 0: TisN0M0

Stage I: TiN0M0

Stage II: T0-1N1M0, T2N0-1M0

Stage III: T0-2N2M0, T3N0-2M0

Stage IVA: T0-3N3M0 or T4N0-3M0

Stage IVB: Any T, N and M1

*The Chinese version is quoted from: China Nasopharyngeal Carcinoma Staging 2017 Edition (2008 Revision of Expert Consensus on Nasopharyngeal Carcinoma Staging) *Chinese Journal of Radiation Oncology*, October 2017, Vol. 26, No. 10 p1119-25

Annex IV Response Evaluation Criteria in Solid Tumors

Response Evaluation Criteria in Solid Tumors: Revised Version 1.1 (Excerpt)

Note: This annex is only used as internal translation data for reference. In actual operation, the English version shall prevail.

1 Background

Omitted

2 Objective

Omitted

3 Measurability of tumor at baseline

3.1 Definitions

At baseline, tumor lesions/lymph nodes will be categorized measurable and non-measurable as follows:

3.1.1 Measurable lesions

Tumor lesions: Must be accurately measured in at least one dimension (longest diameter in the plane of measurement is to be recorded) with a minimum size of:

- 10 mm by CT scan (CT scan slice thickness no greater than 5 mm).
- 10 mm caliper measurement by clinical exam (lesions which cannot be accurately measured with calipers should be recorded as non-measurable).
- 20 mm by chest X-ray.
- Malignant lymph nodes: To be considered pathologically enlarged and measurable, a lymph node must be ≥15 mm in short axis when assessed by CT scan (CT scan slice thickness recommended to be no greater than 5 mm). At baseline and in follow-up, only the short axis will be measured and followed.

3.1.2 Non-measurable lesions

All other lesions, including small lesions (longest diameter <10 mm or pathological lymph nodes with ≥10 mm to <15 mm short axis) as well as non-measurable lesions. Lesions considered non-measurable include: leptomeningeal disease, ascites, pleural or pericardial effusion, inflammatory breast disease, carcinomatous lymphangitic of skin or lung, abdominal masses that cannot be diagnosed and followed up by imaging techniques, and cystic lesions.

3.1.3 Special considerations regarding lesion measurability

Bone lesions, cystic lesions, and lesions previously treated with local therapy require particular comment:

Bone lesions:

- Bone scan, PET scan or plain films are not considered adequate imaging techniques to measure bone lesions. However, these techniques can be used to confirm the presence or disappearance of bone lesions;
- Osteolytic lesions or mixed osteolytic/osteogenic lesions, with identifiable soft tissue components, that can be evaluated by cross sectional imaging techniques such as CT or MRI can be considered as measurable lesions if the soft tissue component meets the definition of measurability described above;
- Osteogenic lesions are non-measurable.

Cystic lesions:

- Lesions that meet the criteria for radiographically defined simple cysts should not be considered as malignant lesions (neither measurable nor non-measurable) since they are, by definition, simple cysts;
- 'Cystic lesions' thought to represent cystic metastases can be considered as measurable lesions, if they meet the definition of measurability described above. However, if non-cystic lesions are present in the same subject, these are preferred for selection as target lesions.

Lesions with prior local treatment:

- Tumor lesions situated in a previously irradiated area, or in an area subjected to other loco-regional therapy, are usually not considered measurable unless there has been demonstrated progression in the lesion. Study protocols should detail the conditions under which such lesions would be considered measurable.

3.2 Specifications by methods of measurements

3.2.1 Measurement of lesions

All measurements should be recorded in metric notation during clinical assessment. All baseline evaluations should be performed as close as possible to the treatment start and never more than 28 days (4 weeks) before the beginning of the treatment.

3.2.2 Method of assessment

The same method of assessment and the same technique should be used to characterize each identified and reported lesion at baseline and during follow-up. Imaging based evaluation should always be done rather than clinical examination unless the lesion(s) being followed cannot be imaged but are assessable by clinical exam.

Clinical lesions: Clinical lesions will only be considered measurable when they are superficial and ≥10 mm diameter as assessed using calipers (e.g. skin nodules). For the case of skin lesions, documentation by color photography including a ruler to estimate the size of the lesion is suggested. When lesions can be evaluated by both imaging and clinical exam, imaging evaluation should be undertaken since it is more objective and may also be reviewed at the end of the study.

Chest X-ray: Chest CT is preferred over chest X-ray, particularly when progression is an important endpoint, since CT is more sensitive than X-ray, particularly in identifying new lesions. However, lesions on chest X-ray may be considered measurable if they are clearly defined and surrounded by aerated lung.

CT, MRI: CT is the best currently available and reproducible method to measure lesions for response assessment. This guideline has defined measurability of lesions on CT scan based on the assumption that CT slice thickness is ≤5 mm. When CT scans have slice thickness greater than 5 mm, the minimum size for a measurable lesion should be twice the slice thickness. MRI is also acceptable in certain situations (e.g. for body scans).

Ultrasound: Ultrasound should not be used as a method of measurement for the measurement of lesion size. Ultrasound examinations cannot be reproduced in their entirety at the end of measurement, because they are operator dependent, it cannot be guaranteed that the same technique and measurements will be taken from one assessment to the next. If new lesions are identified by ultrasound in the course of the study, confirmation by CT or MRI is advised. If there is concern about radiation exposure at CT, MRI may be used instead of CT in selected instances.

Endoscopy, laparoscopy: The utilization of these techniques for objective tumor evaluation is not advised. However, they can be useful to confirm CR when biopsies are obtained or to determine relapse in trials where recurrence following CR or surgical resection is an endpoint.

Tumor markers: Tumor markers alone cannot be used to assess objective tumor response. If markers are above the upper normal limit at baseline, they must normalize for the assessment of complete response. Because tumor markers are disease specific, instructions for their measurement should be incorporated into protocols on a disease specific basis. Specific guidelines for both CA125 response (in recurrent ovarian cancer) and PSA response (in recurrent prostate cancer) have been published. In addition, the Gynecologic Cancer Intergroup has developed CA125 progression criteria which are to be integrated into the objective tumor assessment criteria for first-line treatment regimens of ovarian cancer.

Cytology/histology: These techniques can be used to differentiate between PR and CR in rare cases if required by protocol (for example, residual lesions in tumor types such as germ cell tumors, where known residual benign tumors can remain). When effusions are known to be a potential adverse effect of treatment (e.g. with certain taxane compounds or angiogenesis inhibitors), the cytological confirmation of the neoplastic origin of any effusion that appears or worsens during treatment can be considered if the measurable tumor has met criteria for response or stable disease in order to differentiate between response (or stable disease) and progressive disease.

4 Tumor response criteria

4.1 Assessment of overall tumor burden and measurable disease

To assess objective response or future progression, it is necessary to estimate the overall tumor burden at baseline and use this as a comparator for subsequent measurements. Only subjects with measurable disease at baseline should be included in clinical protocols where objective tumor response is the primary endpoint. Measurable disease is defined by the presence of at least one measurable lesion. In studies where the primary endpoint is tumor progression (either time to progression or degree of progression at a fixed date), the protocol must specify if entry is restricted to those with measurable disease or whether subjects having non-measurable disease only are also eligible.

4.2 Baseline documentation of target and non-target lesions

When more than one measurable lesion is present at baseline evaluation, all lesions should be recorded and measured. Up to a maximum of 5 lesions in total (and a maximum of 2 lesions per organ) representative of all involved organs should be identified as target lesions (this means in instances where subjects have only one or two organ sites involved, a maximum of two and four lesions respectively will be recorded).

Target lesions should be selected on the basis of their size (lesions with the longest diameter), be representative of all involved organs, but in addition should be those that lend themselves to reproducible repeated measurements. It may be the case that, on occasion, the largest lesion does not lend itself to reproducible measurement in which circumstance the next largest lesion which can be measured reproducibly should be selected.

Lymph nodes merit special mention since they are normal anatomical structures which may be visible by imaging even if not involved by tumor. Pathological nodes which are defined as measurable and may be identified as target lesions must meet the criterion of a short axis of ≥15 mm by CT scan. Only the short axis of these nodes will contribute to the baseline sum. The short axis of the node is the diameter normally used by radiologists to judge if a node is involved by solid tumor. Nodal size is normally reported as two dimensions in the plane in which the image is obtained (for CT scan this is almost always the axial plane; for MRI the plane of acquisition may be axial, saggital or coronal). The smaller of these measures is the short axis. For example, an abdominal node which is reported as being 20 mm × 30 mm has a short axis of 20 mm and qualifies as a malignant, measurable node. In this example, 20 mm should be recorded as the node measurement. All other pathological nodes (those with short axis ≥10 mm but <15 mm) should be considered non-target lesions. Nodes that have a short axis <10 mm are considered non-pathological and should not be recorded or followed.

A sum of the diameters (longest for non-nodal lesions, short axis for nodal lesions) for all target lesions will be calculated and reported as the baseline sum diameters. If lymph nodes are to be included in the sum, then as noted above, only the short axis is added into the sum. The baseline sum diameters will be used as reference for baseline levels of the disease.

All other lesions including pathological lymph nodes should be identified as non-target lesions and should also be recorded at baseline. Measurements are not required and these lesions should be followed as ‘present’, ‘absent’, or in rare cases ‘unequivocal progression’. In addition, it is possible to record multiple non-target lesions involving the same organ as a single item (e.g. ‘multiple enlarged pelvic lymph nodes’ or ‘multiple liver metastases’).

4.3 Response criteria

4.3.1 Evaluation of target lesions

Complete Response (CR): Disappearance of all target lesions. Any pathological lymph nodes (whether target or non-target) must have reduction in short axis to <10 mm.

Partial Response (PR): At least a 30% decrease from baseline in the sum of diameters of target lesions.

Progressive Disease (PD): At least a 20% increase in the sum of diameters of target lesions, taking as reference the smallest sum on study (this includes the baseline sum if that is the smallest on study). In addition, the sum of diameters must also demonstrate an absolute increase of at least 5 mm (the appearance of one or more new lesions is also considered progression).

Stable Disease (SD): Neither sufficient shrinkage to qualify for PR nor sufficient increase to qualify for PD, taking as reference the smallest sum diameters while on study.

4.3.2 Special notes on the assessment of target lesions

Lymph nodes. Lymph nodes identified as target lesions should always have the actual short axis measurement recorded (measured in the same anatomical plane as the baseline examination), even if the nodes regress to below 10 mm on study. This means that when lymph nodes are included as target lesions, it cannot be considered that the lesions have disappeared completely even if complete response criteria are met, since abnormal lymph node is defined as having a short axis of <10 mm. Case Report Forms (CRFs) or other data collection methods may therefore be designed to have target nodal lesions recorded in a separate section where, in order to qualify for CR, each node must achieve a short axis <10 mm. For PR, SD and PD, the actual short axis measurement of the nodes is to be included in the sum of target lesions.

Target lesions that become too small to measure: while in the clinical study, all lesions (nodal and non-nodal) recorded at baseline should have their actual measurements recorded at each subsequent evaluation, even when very small (e.g. 2 mm). However, sometimes lesions or lymph nodes which are recorded as target lesions at baseline become so faint on CT scan that the radiologist may not feel comfortable assigning an exact measure and may report them as being ‘too small to measure’. When this occurs, it is important that a value be recorded on the CRF. If it is the opinion of the radiologist that the lesion has likely disappeared, the measurement should be recorded as 0 mm. If the lesion is believed to be present and is faintly seen but too small to measure, a default value of 5 mm should be assigned (Note: It is less likely that this rule will be used for lymph nodes since they usually have a definable size when normal and are frequently surrounded by fat such as in the retroperitoneum; however, if a lymph node is believed to be present and is faintly seen but too small to measure, a default value of 5 mm should be assigned in

this circumstance as well). This default value is derived from the 5 mm CT slice thickness (but should not be changed with varying CT slice thickness). The measurement of these lesions is potentially non-reproducible, therefore providing this default value will prevent false responses or progressions based upon measurement error. To reiterate, however, if the radiologist is able to provide an actual measure, that should be recorded, even if it is below 5 mm.

Lesions that split or coalesce on treatment: when non-nodal lesions fragment, the longest diameters of the fragmented portions should be added together to calculate the target lesion sum. Similarly, as lesions coalesce, a plane between them may be maintained that would aid in obtaining maximal diameter measurements of each individual lesion. If the lesions have truly coalesced such that they are no longer separable, the vector of the longest diameter in this instance should be the maximal longest diameter for the coalesced lesion.

4.3.3 Evaluation of non-target lesions

This section provides the definitions of the criteria used to determine the tumor response for the group of non-target lesions. While some non-target lesions may actually be measurable, they need not be measured and instead should be assessed only qualitatively at the time points specified in the protocol.

Complete Response (CR): Disappearance of all non-target lesions and normalization of tumor marker level. All lymph nodes must be non-pathological in size (<10 mm short axis).

Non-CR/Non-PD: Persistence of one or more non-target lesion(s) and/or maintenance of tumor marker level above the normal limits.

Progressive Disease (PD): Unequivocal progression of existing non-target lesions. Note: the appearance of one or more new lesions is also considered progression.

4.3.4 Special notes on assessment of progression of non-target disease

The concept of progression of non-target disease requires additional explanation as follows: When the subject also has measurable disease. In this setting, to achieve unequivocal progression on the basis of the non-target disease, there must be an overall level of substantial worsening in non-target disease such that, even in presence of SD or PR in target disease, the overall tumor burden has increased sufficiently to merit discontinuation of therapy. A modest increase in the size of one or more non-target lesions is usually not sufficient to quality for unequivocal progression status. The designation of overall progression solely on the basis of change in non-target disease in the face of SD or PR of target disease will therefore be extremely rare.

When the subject has only non-measurable disease: this circumstance arises in some phase III trials when it is not a criterion of study entry to have measurable disease. The same general concepts apply here as noted above, however, in this instance there is no measurable disease assessment to factor into the interpretation of an increase in non-measurable disease burden. Because worsening in non-target disease cannot be easily quantified (by definition: if all lesions are truly non-measurable) a useful test that can be applied when assessing subjects for unequivocal progression is to consider if the increase in overall disease burden based on the change in non-measurable disease is comparable in magnitude to the increase that would be required to declare PD for measurable disease: i.e. an increase in tumor burden representing an additional 73% increase in "volume" (which is equivalent to a 20% increase diameter in a measurable lesion). Examples include an increase in a pleural effusion from 'trace' to 'large', an increase in lymphangitic disease from localized to widespread, or may be described in protocols as 'sufficient to require a change in therapy'. If unequivocal progression is seen, the subject should be considered to have had overall PD at that point. While it would be ideal to have objective criteria to apply to non-measurable disease, the very nature of that disease makes it impossible to do so, therefore the increase must be substantial.

4.3.5 New lesions

The appearance of new malignant lesions denotes disease progression; therefore, some comments on detection of new lesions are important. There are no specific criteria for the identification of new radiographic lesions; however, the finding of a new lesion should be unequivocal: i.e. not attributable to differences in scanning technique, change in imaging modality or findings thought to represent something other than tumor (for example, some new bone lesions may be simply healing or flare of pre-existing lesions). This is particularly important when the subject's baseline lesions show partial or complete response. For example, necrosis of a liver lesion may be reported on a CT scan report as a new cystic lesion, which it is not.

A lesion identified on a follow-up study in an anatomical location that was not scanned at baseline is considered a new lesion and will indicate disease progression. An example of this is the subject who has visceral disease at baseline and while on study has a CT or MRI brain ordered which reveals metastases. The subject's brain metastases are considered to be evidence of progressive disease even if he/she did not have brain imaging at baseline.

If a new lesion is equivocal, for example because of its small size, continued therapy and follow-up evaluation will clarify if it represents truly new disease. If repeat scans confirm there is definitely a new lesion, then progression should be declared using the date of the initial scan.

While FDG-PET response assessments need additional study, it is sometimes reasonable to incorporate the use of FDG-PET scanning to complement CT scanning in assessment of progression (particularly possible new disease). New lesions on the basis of FDG-PET imaging can be identified according to the following algorithm:

Negative FDG-PET at baseline, with a positive FDG-PET at follow-up is a sign of PD based on a new lesion.

No FDG-PET at baseline and a positive FDG-PET at follow-up:

If the positive FDG-PET at follow-up corresponds to a new site of disease confirmed by CT, this is PD.

If the positive FDG-PET at follow-up is not confirmed as a new site of disease on CT, additional follow-up CT scans are needed to determine if there is truly progression occurring at that site (if so, the date of PD will be the date of the initial abnormal FDG-PET scan).

If the positive FDG-PET at follow-up corresponds to a pre-existing site of disease on CT that is not progressing on the basis of the anatomic images, this is not PD.

4.4 Evaluation of best overall response

The best overall response is the best response recorded from the start of the study treatment until the end of treatment taking into account any requirement for confirmation. On occasion a response may not be documented until after the end of therapy so protocols should be clear if post-treatment assessments are to be considered in determination of best overall response. Protocols must specify how any new therapy introduced before progression will affect best response designation. The subject's best overall response assignment will depend on the findings of both target and non-target disease and will also take into consideration the appearance of new lesions. Furthermore, it also depends on the nature of the study, the protocol requirements, and criteria for result measurement. Specifically, in non-randomized trials where response is the primary endpoint, confirmation of PR or CR is needed to deem either one the best overall response.

4.4.1 Time point response

It is assumed that at each protocol specified time point, a response assessment occurs. Table 1 provides a summary of the overall response status calculation at each time point for subjects who have measurable disease at baseline.

Table 1 Time point reaction: subjects with target (+/- non-target) disease

Target lesions	Non-target lesions	New lesions	Overall response
CR	CR	No	CR
CR	Non-CR/non-PD	No	PR
CR	Not evaluated	No	PR
PR	Non-PD or not all evaluated	No	PR
SD	Non-PD or not all evaluated	No	SD
Not all evaluated	Non-PD	No	NE
PD	Any	Yes or No	PD
Any	PD	Yes or No	PD
Any	Any	Yes	PD

Note:CR=complete response, PR= partial response, SD=stable disease, PD=progressive disease, and NE=non-evaluable.

When subjects have non-measurable (non-target) disease only, Table 2 is to be used.

Table 2 Time point response: subjects with non-target disease only

Non-target lesions	New lesions	Overall response
CR	No	CR
Non-CR/non-PD	No	Non-CR/Non-PD[a]
Not fully evaluated	No	NE
Unequivocal PD	Yes or No	PD
Any	Yes	PD

Note:a 'Non-CR/non-PD' is preferred over 'stable disease' for non-target disease. Since SD is increasingly used as endpoint for assessment of efficacy in some trials, non-CR/non-PD efficacy has been developed so to assign this category when no lesions can be measured is not advised.

4.4.2 Missing assessments and inevaluable designation

When no imaging/measurement is done at all at a particular time point, the subject is not evaluable (NE) at that time point. If only a subset of lesion measurements are made at an assessment, usually the case is also considered NE at that time point, unless a convincing argument can be made that the contribution of the individual missing lesion(s) would not change the assigned time point response. This would be most likely to happen in the case of PD. For example, if a subject had a baseline sum of 50 mm with 3 measured lesions and at follow-up only two lesions were assessed, but those gave a sum of 80 mm, the subject will have achieved PD status, regardless of the contribution of the missing lesion.

4.4.3 Best overall response: all time points

The best overall response is determined once all the data for the subject is known.

Best response determination in trials where confirmation of complete or partial response IS NOT required: Best response in these trials is defined as the best response across all time points (for example, a subject who has SD at first assessment, PR at second assessment, and PD on last assessment has a best overall response of PR). When SD is believed to be best response, it must also meet the protocol specified minimum time from baseline. If the minimum time is not met when SD is otherwise the best time point response, the subject's best response depends on the subsequent assessments. For example, a subject who has SD at first assessment, PD at second and does not meet minimum duration for SD, will have a best response of PD. The same subject lost to follow-up after the first SD assessment would be considered inevaluable.

Best response determination in trials where confirmation of complete or partial response IS required: Complete or partial responses may be claimed only if the criteria for each are met at a subsequent time point as specified in the protocol (generally four weeks later). In this circumstance, the best overall response can be interpreted as in Table 3.

Table 3 Best overall response when confirmation of CR and PR required.

Overall response First time point	Overall response Subsequent time point	BEST overall response
CR	CR	CR
CR	PR	SD, PD or PR[a]
CR	SD	SD provided minimum criteria for SD duration met, otherwise, PD
CR	PD	SD provided minimum criteria for SD duration met, otherwise, PD
CR	NE	SD provided minimum criteria for SD duration met, otherwise NE
PR	CR	PR
PR	PR	PR
PR	SD	SD
PR	PD	SD provided minimum criteria for SD duration met, otherwise, PD
PR	NE	SD provided minimum criteria for SD duration met, otherwise NE
NE	NE	NE

Note: CR = complete response, PR = partial response, SD = stable disease, PD = progressive disease, and NE = non-evaluable.

Superscript "a": If a CR is truly met at first time point, then any disease seen at a subsequent time point, even disease meeting PR criteria relative to baseline, makes the disease PD at that point (since disease must have reappeared after CR Best response would depend on whether minimum duration for SD was met. However, sometimes 'CR' may be claimed when subsequent scans suggest small lesions were likely still present and in fact the subject had PR, not CR at the first time point. Under these circumstances, the original CR should be changed to PR and the best response is PR.

4.4.4 Special notes on response assessment

When nodal disease is included in the sum of target lesions and the nodes decrease to 'normal' size (<10 mm), they may still have a measurement reported on scans. This measurement should be recorded even though the nodes are normal in order not To overstate progression should it based on increase in size of the nodes. As noted earlier, this means that subjects with CR may not have a total sum of 0 on the case report form (CRF).

In trials where confirmation of response is required, repeated 'NE' time point assessments may complicate best response determination. The analysis plan for the trial must address how missing data/assessments will be addressed in determination of response and progression. For example, in most Trials it is reasonable to consider a subject with time point responses of PR-NE-PR as a confirmed response.

Subjects with a global deterioration of health status requiring discontinuation of treatment without objective evidence of disease progression at that time should be reported as symptom deterioration. Every effort should be made to document objective progression even after discontinuation of treatment. Symptom deterioration is not a descriptor of The objective response status of such subjects is determined by evaluation of target and non-target disease as shown in Tables 1-3.

Conditions that define early progression, early death and inevaluable are study specific and should be clearly described in each protocol (depending on treatment duration, treatment cycle).

In some circumstances, it may be difficult to distinguish residual disease from normal tissue. When the evaluation of complete response depends upon this determination, it is recommended that the residual lesion be investigated before assigning a status of complete response. FDG-PET may be used To upgrade a response to a CR in a manner similar to a biopsy in cases where a residual radiographic abnormality is thought to represent fibrosis or scarring. The use of FDG-PET in this circumstance should be prospectively described in the protocol and supported by disease specific However, it must be acknowledged that both approaches may lead to false positive CR due to limitations of FDG-PET and biopsy itself (including resolution/sensitivity of the two).

For equivocal findings of progression (eg very small and uncertain new lesions; cystic changes or necrosis in existing lesions), treatment may continue until the next scheduled assessment, if at the next scheduled assessment, progression is confirmed, the date of progression should be the Earlier date when progression was suspected.

4.5 Frequency of tumor re-evaluation

Frequency of tumor re-evaluation while on treatment should be protocol specific and adapted to the type and schedule of treatment. However, in the context of phase II studies where the beneficial effect of therapy is not known, follow-up every 6–8 weeks (timed to coincide with the end of a cycle) is reasonable. Smaller or greater time intervals than these could be justified in specific regimens or circumstances. The protocol should specify which organ sites are to be evaluated at baseline (usually those most likely to be involved with metastatic disease for the tumor type under study) and how often evaluations are repeated. Normally, all target and non-target sites are evaluated at each assessment. In selected circumstances certain non-target organs may be evaluated less frequently. For example, bone scans may need to be repeated only when complete response is identified in target disease or when progression in bone is suspected.

After the end of the treatment, the need for repetitive tumor evaluations depends on whether the trial has as a goal the response rate or the time to an event (progression/death). If time to an event (e.g. TTP/DFS/PFS) is the main endpoint of the study, then routine scheduled re-evaluation of protocol specified sites of disease is warranted. In randomized comparative trials in particular, the scheduled assessments should be performed as identified on a calendar schedule (for example: every 6–8 weeks on treatment or every 3–4 months after treatment) and should not be affected by delays in therapy, drug holidays or any other events that might lead to imbalance in a treatment arm in the timing of disease assessment.

4.6 Response evaluation/confirmation of duration of response

4.6.1 Confirmation

In non-randomized trials where response is the primary endpoint, confirmation of PR and CR is required to ensure responses identified are not the result of measurement error. This will also permit appropriate interpretation of results in the context of historical data where response has traditionally required confirmation in such trials. However, in all other circumstances, e.g. in randomized trials (phase II or III) or studies where stable disease or progression are the primary endpoints, confirmation of response is not required since it will not add value to the interpretation of trial results. However, elimination of the requirement for response confirmation may increase the importance of central review to protect against bias, in particular in studies which are not blinded.

In the case of SD, measurements must have met the SD criteria at least once after study entry at a minimum interval (in general not less than 6–8 weeks) that is defined in the study protocol.

4.6.2 Duration of overall response

The duration of overall response is measured from the time measurement criteria are first met for CR/PR (whichever is first recorded) until the first date that recurrent or progressive disease is objectively documented (taking as reference for progressive disease the smallest measurements recorded on study). The duration of overall complete response is measured from the time measurement criteria are first met for CR until the first date that recurrent disease is objectively documented.

4.6.3 Duration of stable disease

Stable disease is measured from the start of the treatment (in randomized trials, from date of randomization) until the criteria for progression are met, taking as reference the smallest sum on study (if the baseline sum is the smallest, this is the reference for calculation of PD). The clinical relevance of the duration of stable disease varies in different studies and diseases. If the proportion of subjects achieving stable disease for a minimum period of time is an endpoint of importance in a particular trial, the protocol should specify the minimal time interval required between two measurements for determination of stable disease.

Note: The duration of response and stable disease as well as the progression-free survival are influenced by the frequency of follow-up after baseline evaluation. It is not in the scope of this guideline to define a standard follow-up frequency. The frequency should take into account many parameters including disease types and stages, treatment cycle and standard practice. However, these limitations of the precision of the measured endpoint should be taken into account if comparisons between trials are to be made.

4.7 PFS/TTP

4.7.1 Phase II clinical trials

This guideline is focused primarily on the use of objective response endpoints for phase II trials. In some circumstances, ‘response rate’ may not be the optimal method to assess the potential anticancer activity of new agents/regimens. In such cases ‘progression-free survival’ (PFS) or the ‘proportion progression-free’ (PPF) at landmark time points, might be considered appropriate alternatives to provide an initial signal of biologic effect of new agents. It is clear, however, that in an uncontrolled trial, these measures are subject to criticism since an apparently promising observation may be related to biological factors such as subjects election and not the impact of the intervention. Thus, phase II screening trials utilizing these endpoints are best designed with a randomized control. Exceptions may exist where the behavior patterns of certain cancers are so consistent (and usually consistently poor), that a non-randomized trial is justifiable. However, in these cases it will be essential to document with care the basis for estimating the expected PFS or PPF in the absence of positive controls.

Annex V Chinese Medicines Prohibited in the Trial Period

Chinese medicines prohibited	
Huatanhuisheng Tablets	Kang'aiping Pills
Brucea Javanica Oil Soft Capsules	Fukang Capsules
Tuomu Syrup	Xiaoaiping
Cantharidis	Pingxiao Capsules
Cinobufotalin	Pingxiao Tablets
Senso	Shendansanjie Capsules
Kang'an Injection	Ankangxin Capsules
Kangleite	Bosheng'aining
Zhongjiefeng Injection	Zedoary Turmeric Oil and Glucose Injection
Aidi Injection	Kanglixin Capsules
Awei Huapi Plaster	Cidan Capsules
