# Supplementary material for: Five-Year Outcome of Camrelizumab Plus Chemotherapy in Recurrent or Metastatic Nasopharyngeal Carcinoma: A Secondary Analysis of the CAPTAIN-1st Randomized Clinical Trial
Source: JAMA Oncol. 2026 Jan 29;12(3):295–302. doi: 10.1001/jamaoncol.2025.6245 (PMC12856745; doi:10.1001/jamaoncol.2025.6245)
Supplement: Supplement 2. — eTable 1. Patient disposition by site eTable 2. Summary of study drug exposure at data cutoff (Dec 5, 2024) eTable 3. Subsequent anti-cancer therapies in intent-to-treat population after the study treatment eTable 4. Subsequent anti-cancer therapies in EBV DNA negative population after the study treatment eTable 5. Baseline demographics and disease characteristics of ≥ 5-year and < 5-year survivors eFigure 1. The examination of Schoenfeld residuals from stratified Cox proportional regression model for overall survival eFigure 2. Overall survival by age in the camrelizumab and placebo groups eFigure 3. Dynamic changes in plasma EBV DNA copy numbers during two years of treatment eFigure 4. Overall survival by plasma EBV DNA clearance in the placebo group eFigure 5. Overall survival by plasma EBV DNA clearance between camrelizumab and placebo groups eFigure 6. Overall survival by completion or discontinuation of 2 years of camrelizumab treatment [file jamaoncol-e256245-s002.pdf]

Supplemental Online Content

Huang Y, Sun D, Zhou H, et al. 5-Year outcome of camrelizumab plus chemotherapy in recurrent or metastatic nasopharyngeal carcinoma: the CAPTAIN-1st randomized clinical trial. *JAMA Oncol*. Published online January 29, 2026. doi:10.1001/jamaoncol.2025.6245

eTable 1. Patient disposition by site
eTable 2. Summary of study drug exposure at data cutoff (Dec 5, 2024)
eTable 3. Subsequent anti-cancer therapies in intent-to-treat population after the study treatment
eTable 4. Subsequent anti-cancer therapies in EBV DNA negative population after the study treatment
eTable 5. Baseline demographics and disease characteristics of ≥ 5-year and < 5-year survivors
eFigure 1. The examination of Schoenfeld residuals from stratified Cox proportional regression model for overall survival
eFigure 2. Overall survival by age in the camrelizumab and placebo groups
eFigure 3. Dynamic changes in plasma EBV DNA copy numbers during two years of treatment
eFigure 4. Overall survival by plasma EBV DNA clearance in the placebo group
eFigure 5. Overall survival by plasma EBV DNA clearance between camrelizumab and placebo groups
eFigure 6. Overall survival by completion or discontinuation of 2 years of camrelizumab treatment

This supplemental material has been provided by the authors to give readers additional information about their work.

© Huang et al, *JAMA Oncology*.

Supplementary Tables and Figures

Name of study site	Location	Enrolled patients, n (%)
Sun Yat-sen University Cancer Center	Guangzhou	93 (35.4)
Guangxi Medical University Affiliated Tumor Hospital	Nanning	17 (6.5)
Jiangxi Cancer Hospital of Nanchang University	Nanchang	14 (5.3)
Fudan University Shanghai Cancer Center	Shanghai	13 (4.9)
First Affiliated Hospital of Gannan Medical University	Ganzhou	10 (3.8)
Guangzhou Medical University Affiliated Oncology Hospital	Guangzhou	10 (3.8)
Xiangya Hospital Central South University	Changsha	9 (3.4)
Affiliated Cancer Hospital of Zhengzhou University	Zhengzhou	8 (3.0)
Sichuan Cancer Hospital & Institute, Sichuan Cancer Center	Chengdu	8 (3.0)
Tongji Hospital of Tongji Medical College	Wuhan	7 (2.7)
Liuzhou People's Hospital	Liuzhou	7 (2.7)
900th Hospital of the Joint Logistics Team	Fuzhou	7 (2.7)
Guangxi Zhuang Autonomous Region People's Hospital	Nanning	6 (2.3)
The First People's Hospital of Foshan	Foshan	6 (2.3)
Zhejiang Cancer Hospital	Hangzhou	6 (2.3)
Fujian Medical University Cancer Hospital	Fuzhou	5 (1.9)
General Hospital of Southern Theatre Command	Guangzhou	5 (1.9)
Yunnan Cancer Hospital	Kunming	5 (1.9)
Beijing Cancer Hospital	Beijing	5 (1.9)
Cancer Hospital of Shantou University Medical College	Shantou	5 (1.9)
The First Affiliated Hospital of Xiamen University	Xiamen	4 (1.5)
Zhongshan People's Hospital Feng Lei	Zhongshan	4 (1.5)
The First Affiliated Hospital of Sun Yat-sen University	Guangzhou	2 (0.8)
The First Affiliated Hospital of Fujian Medical University	Fuzhou	2 (0.8)
Sun Yat-sen Memorial Hospital of Sun Yat-sen University	Guangzhou	2 (0.8)
Jiujiang No.1 People's Hospital of Nanchang University	Jiujiang	1 (0.4)
Tianjin Medical University Cancer Institute and Hospital	Tianjin	1 (0.4)
Cancer Hospital Chinese Academy of Medical Sciences	Beijing	1 (0.4)

eTable 1: Patient disposition by site.

© Huang et al, *JAMA Oncology*.

	Camrelizumab group (n=134)	Placebo group (n=129)
The number of camrelizumab/placebo cycles		
Median (IQR), cycles	16.0 (9.0–30.0)	10.0 (8.0–17.0)
Treatment duration for camrelizumab/placebo		
Median (IQR), months	12.8 (6.9–22.8)	8.2 (5.9–12.6)
The number of cisplatin cycles		
Median (IQR), cycles	6.0 (5.0-6.0)	6.0 (5.0-6.0)
< 4 cycles	12 (9.0%)	9 (7.0%)
4 cycles	15 (11.2%)	22 (17.1%)
5 cycles	16 (11.9%)	15 (11.6%)
6 cycles	91 (67.9%)	83 (64.3%)
The number of gemcitabine cycles		
Median (IQR), cycles	6.0 (5.0-6.0)	6.0 (5.0-6.0)
< 4 cycles	12 (9.0%)	9 (7.0%)
4 cycles	15 (11.2%)	20 (15.5%)
5 cycles	15 (11.2%)	15 (11.6%)
6 cycles	92 (68.7%)	85 (65.9%)
Reasons for treatment discontinuation		
Disease progression or death	76 (56.7%)	99 (76.7%)
Completion of 2 years exposure	32 (23.9%)	7 (5.4%)
Patient withdrawal	16 (11.9%)	19 (14.7%)
Adverse event	9 (6.7%)	1 (0.8%)
Physical decline	1 (0.7%)	1 (0.8%)
Investigator decision	0 (0%)	2 (1.6%)

eTable 2: Summary of study drug exposure at data cutoff (Dec 5, 2024).

© Huang et al, *JAMA Oncology*.

**Subsequent treatment**	**Camrelizumab group (n=134)**	**Placebo group (n=129)**
**Any**	82 (61.2%)	97 (75.2%)
**Radiotherapy**	32 (23.9%)	29 (22.5%)
**Surgery**	2 (1.5%)	7 (5.4%)
**Systemic therapy**	79 (59.0%)	94 (72.9%)
**Chemotherapy**	68 (50.7%)	90 (69.8%)
**PD-1/PD-L1 inhibitor**	40 (29.9%)	42 (32.6%)
**VEGF/VEGFR inhibitor**	30 (22.4%)	32 (24.8%)
**EGFR inhibitor**	14 (10.4%)	13 (10.1%)
**Others**	7 (5.2%) [*]	3 (2.3%) [#]

eTable 3. Subsequent anti-cancer therapies in intent-to-treat population after the study treatment. [*]4 patients received antibody–drug conjugate (ADC), 1 patient received poly ADP ribose polymerase (PARP) inhibitor, 1 patient received oncolytic virus, 1 patient received bispecific antibody targeting epidermal growth factor receptor (EGFR) and human epidermal growth factor receptor 3 (HER3). [#]1 patient received ADC, 1 patient received src homology region 2-containing protein tyrosine phosphatase-2 (SHP-2) inhibitor, 1 patient received cyclin-dependent kinase 4 and 6 (CDK4/6) inhibitor. EGFR=epidermal growth factor receptor. PD-1=programmed death-1. PD-L1=programmed death ligand 1. VEGF=vascular endothelial growth factor. VEGFR=VEGF receptor.

© Huang et al, *JAMA Oncology*.

**Subsequent treatment**	**Camrelizumab group (n=39)**	**Placebo group (n=43)**
**Any**	18 (46.2%)	29 (67.4%)
**Radiotherapy**	4 (10.3%)	10 (23.3%)
**Surgery**	0 (0.0%)	3 (7.0%)
**Systemic therapy**	17 (43.6%)	27 (62.8%)
**Chemotherapy**	14 (35.9%)	26 (60.5%)
**PD-1/PD-L1 inhibitor**	6 (15.4%)	14 (32.6%)
**VEGF/VEGFR inhibitor**	3 (7.7%)	10 (23.3%)
**EGFR antibody**	3 (7.7%)	4 (9.3%)
**Others**	1 (2.6%) *	1 (2.3%) #

eTable 4: Subsequent anti-cancer therapies in EBV DNA negative population after the study treatment. *1 patient received antibody–drug conjugate (ADC). #1 patient received cyclin-dependent kinase 4 and 6 (CDK4/6) inhibitor. PD-1=programmed death-1. PD-L1=programmed death ligand 1. VEGF=vascular endothelial growth factor. VEGFR=VEGF receptor.

© Huang et al, *JAMA Oncology*.

	Camrelizumab group (n=134)			Placebo group (n=129)		
	≥5-year survivors (n=43)	<5-year survivors (n=91)	P value	≥5-year survivors (n=22)	<5-year survivors (n=107)	P value
**Age**						
<50 years	17 (39.5%)	58 (63.7%)	0.01	5 (22.7%)	51 (47.7%)	0.06
≥50 years	26 (60.5%)	33 (36.3%)		17 (77.3%)	56 (52.3%)	
**Sex**						
Male	37 (86.0%)	76 (83.5%)	0.90	18 (81.8%)	87 (81.3%)	1.00
**ECOG performance status**						
0	20 (46.5%)	27 (29.7%)	0.09	15 (68.2%)	29 (27.1%)	<0.01
1	23 (53.5%)	64 (70.3%)		7 (31.8%)	78 (72.9%)	
**Baseline plasma EBV DNA level**						
Positive	28 (65.1%)	67 (73.6%)	0.42	12 (54.5%)	74 (69.2%)	0.28
Negative	15 (34.9%)	24 (26.4%)		10 (45.5%)	33 (30.8%)	
**Clearance of EBV DNA***						
Rapid EBV clearance*	27 (96.4%)	42 (62.7%)	<0.01	11/12 (91.7%)	42 (56.8%)	0.07
Without rapid clearance	1 (3.6%)	19 (28.4%)		1/12 (8.3%)	22 (29.7%)	
Unknown	0 (0%)	6 (9.0%)		0 (0%)	10 (13.5%)	
**WHO classification**						
Non-keratinizing differentiated	7 (16.3%)	14 (15.4%)	0.99	2 (9.1%)	19 (17.8%)	0.47
Non-keratinizing undifferentiated	35 (81.4%)	75 (82.4%)		20 (90.9%)	86 (80.4%)	
Others	1 (2.3%)	2 (2.2%)		0 (0%)	2 (1.9%)	
**Baseline metastatic sites**						
Liver	19 (44.2%)	51 (56.0%)	0.27	10 (45.5%)	56 (52.3%)	0.72
Lung	22 (51.2%)	44 (48.4%)	0.91	11 (50.0%)	50 (46.7%)	0.96
**Concurrent chemoradiotherapy history**						
Yes	28 (65.1%)	58 (63.7%)	1.00	15 (68.2%)	68 (63.6%)	0.87
No	15 (34.9%)	33 (36.3%)		7 (31.8%)	39 (36.4%)	
**Number of metastatic organs**						
1	15 (34.9%)	29 (31.9%)	0.91	12 (54.5%)	12 (33.6%)	0.03
2	18 (41.9%)	38 (41.8%)		37 (34.6%)	5 (34.6%)	
≥ 3	10 (23.2%)	24 (26.4%)		34 (31.8%)	5 (31.8%)	

eTable 5: Baseline demographics and disease characteristics of ≥ 5-year and < 5-year survivors.

*Rapid EBV DNA clearance was defined as EBV changes from positive to negative after the first three treatment cycles. ECOG=Eastern Cooperative Oncology Group. EBV=Epstein-Barr virus.

© Huang et al, *JAMA Oncology*.

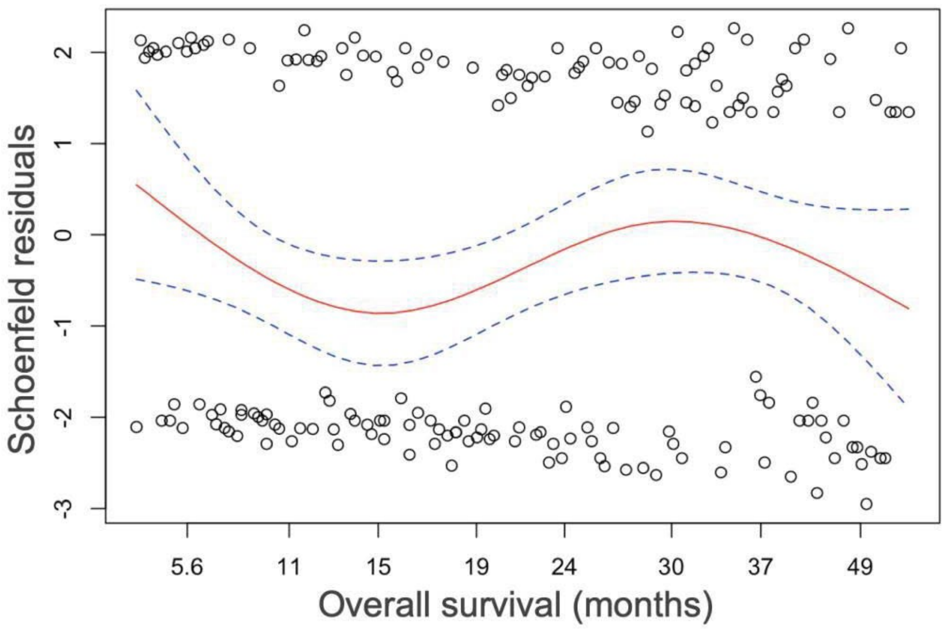

eFigure 1. The examination of Schoenfeld residuals from stratified Cox proportional regression model for overall survival. Stratification factors included liver metastasis, previous radical chemoradiotherapy, and ECOG performance status.

© Huang et al, *JAMA Oncology*.

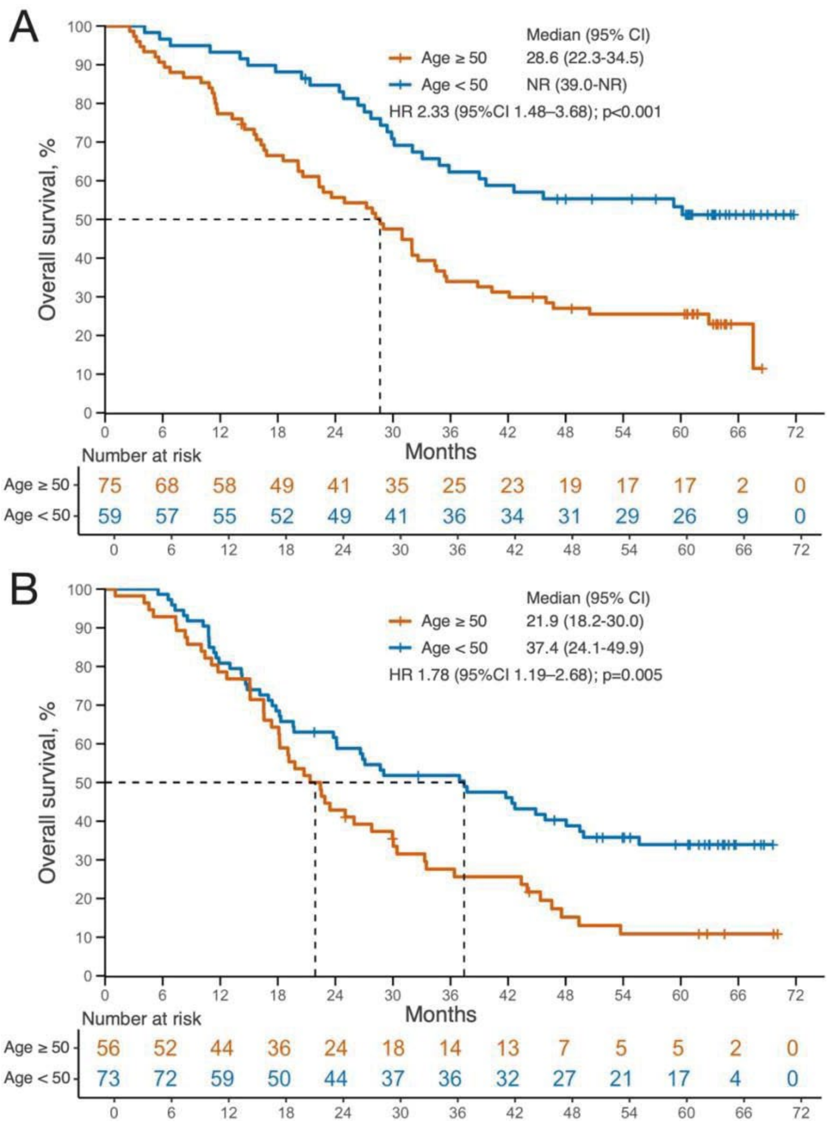

eFigure 2: Overall survival by age in the camrelizumab and placebo groups. Kaplan–Meier curves for overall survival based on age (≥50 versus < 50 years) in the (A) camrelizumab group and (B) placebo group. HR=hazard ratio. NR=not reached.

© Huang et al, *JAMA Oncology*.

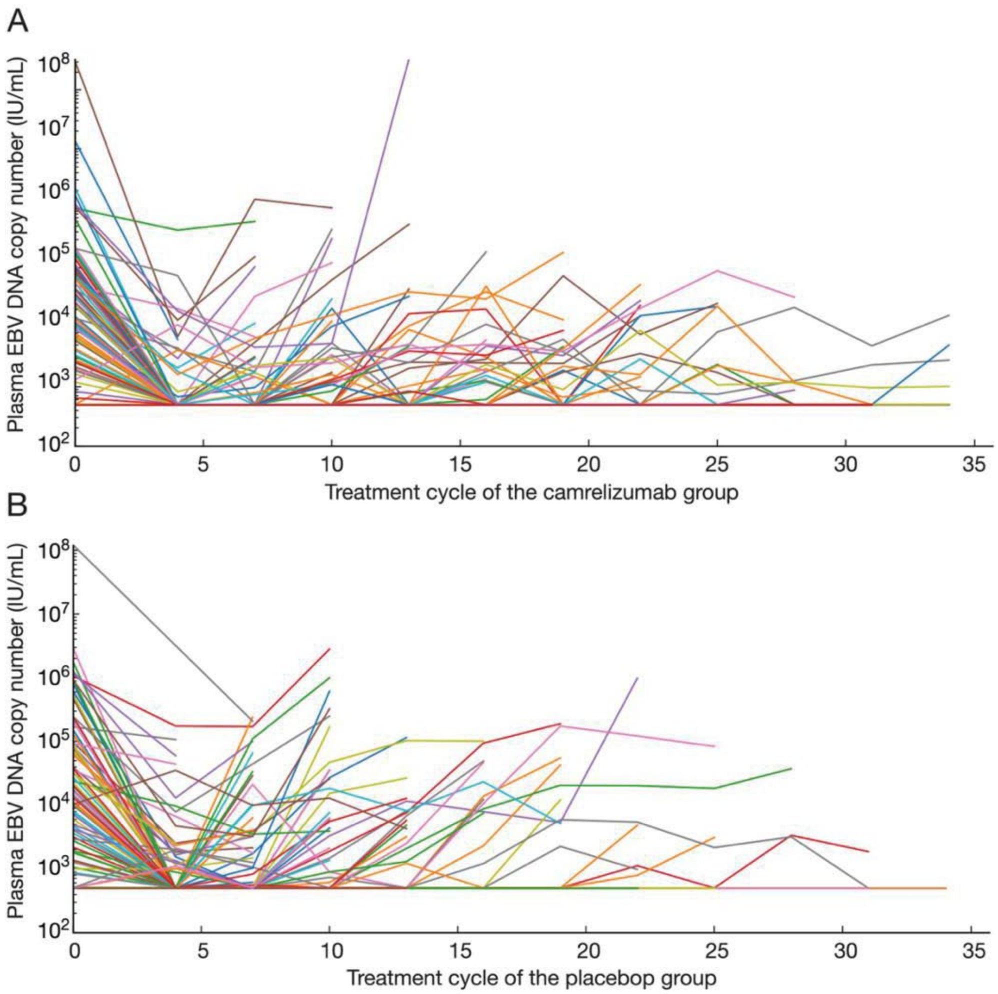

eFigure 3: Dynamic changes in plasma EBV DNA copy numbers during two years of treatment. Longitudinal plasma samples were collected at baseline and every three treatment cycles thereafter for EBV DNA copy number assessment by quantitative PCR. The cutoff was 500 IU/mL. Dynamic EBV DNA copy number trends are shown for (A) camrelizumab group and (B) placebo group. EBV=Epstein-Barr virus.

© Huang et al, *JAMA Oncology*.

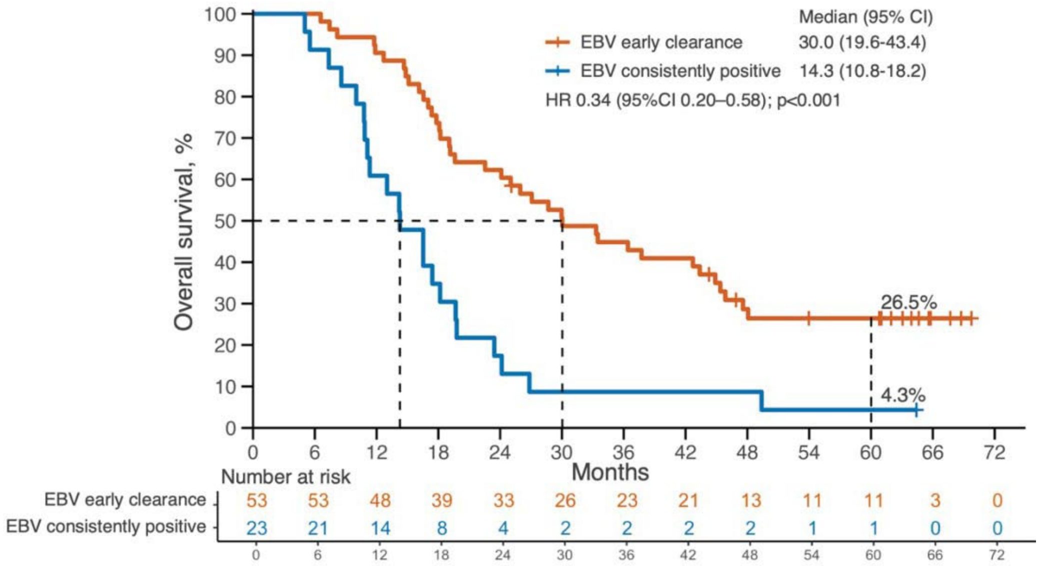

eFigure 4: Overall survival by plasma EBV DNA clearance in the placebo group. Kaplan–Meier curves for overall survival in the placebo group comparing patients who achieved plasma EBV DNA clearance within the first three treatment cycles versus those with persistently positive EBV DNA. EBV=Epstein-Barr virus. HR indicates hazard ratio.

© Huang et al, *JAMA Oncology*.

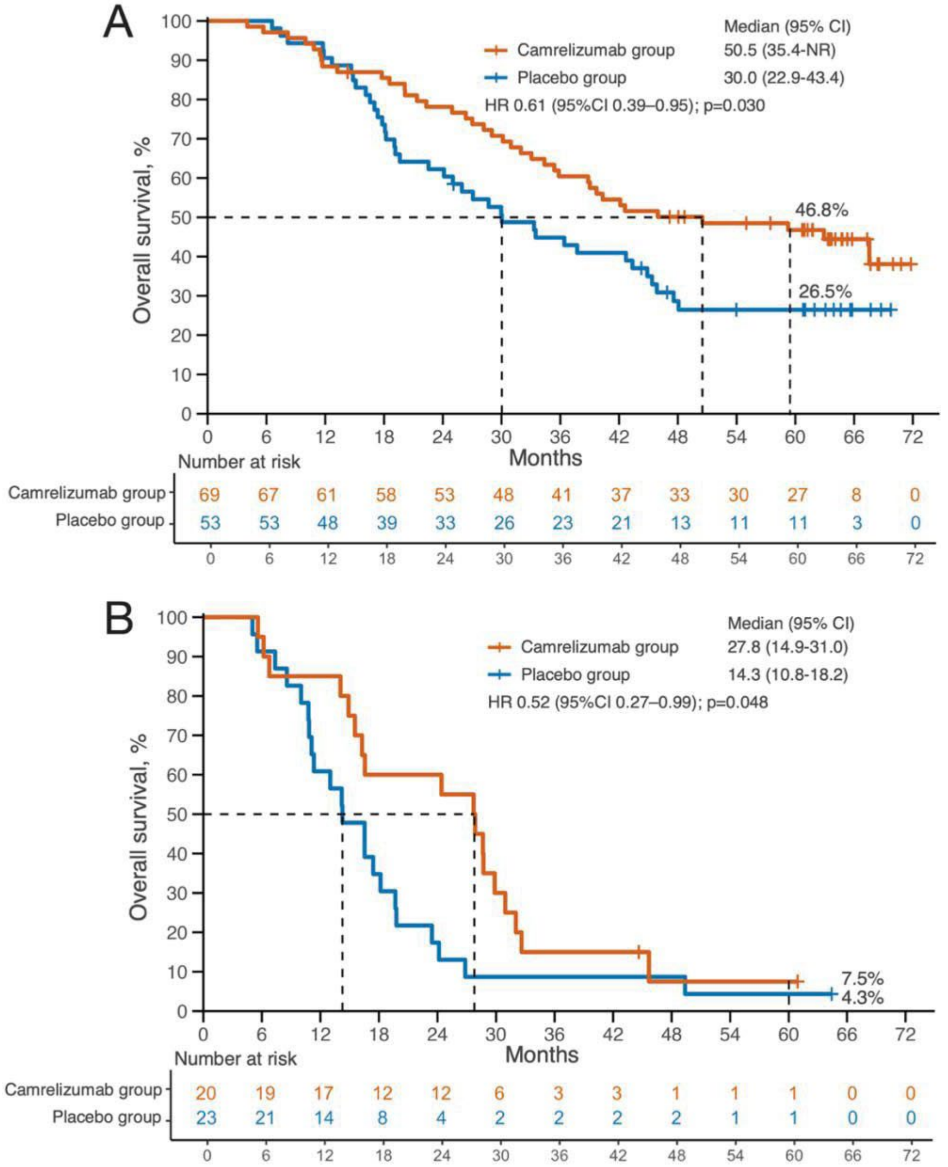

eFigure 5: Overall survival by plasma EBV DNA clearance between camrelizumab and placebo groups. Kaplan–Meier curves for overall survival in (A) patients with plasma EBV DNA clearance within the first three treatment cycles and (B) patients with persistently positive EBV DNA. EBV=Epstein-Barr virus. HR=hazard ratio. NR=not reached.

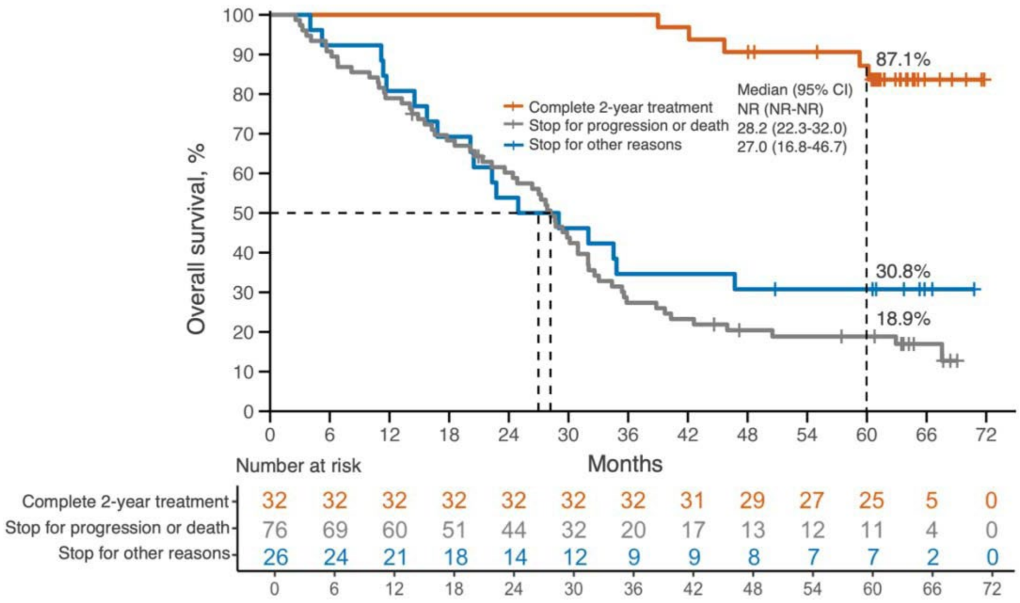

eFigure 6: Overall survival by completion or discontinuation of 2 years of camrelizumab treatment. Kaplan–Meier curves for overall survival among patients who (1) completed the planned 2-year course of camrelizumab, (2) discontinued treatment due to disease progression or death, and (3) discontinued for other reasons such as adverse events, withdrawal, or physical decline. EBV=Epstein-Barr virus. HR=hazard ratio. NR=not reached.
