# Supplementary material for: Five-Year Outcome of Camrelizumab Plus Chemotherapy in Recurrent or Metastatic Nasopharyngeal Carcinoma: A Secondary Analysis of the CAPTAIN-1st Randomized Clinical Trial
Source: JAMA Oncol. 2026 Jan 29;12(3):295–302. doi: 10.1001/jamaoncol.2025.6245 (PMC12856745; doi:10.1001/jamaoncol.2025.6245)
Supplement: Supplement 3. — Data Sharing Statement [file jamaoncol-e256245-s003.pdf]

Data Sharing Statement

Huang. 5-Year Outcome of Camrelizumab Plus Chemotherapy in Recurrent or Metastatic Nasopharyngeal Carcinoma. *JAMA Oncol*. Published January 29, 2026.
doi:10.1001/jamaoncol.2025.6245

Data
Additional Information: NCT03707509
Data available: Yes
Data types: Deidentified participant data
How to access data: Data will be available upon request to the corresponding author.
When available: With publication

Supporting Documents
Document types: None

Additional Information
Who can access the data: Researchers whose proposed use of the data has been approved.
Types of analyses: For scientific purpose.
Mechanisms of data availability: After approval of a proposal.
